# Two novel venom proteins underlie divergent parasitic strategies between a generalist and a specialist parasite

**DOI:** 10.1038/s41467-020-20332-8

**Published:** 2021-01-11

**Authors:** Jianhua Huang, Jiani Chen, Gangqi Fang, Lan Pang, Sicong Zhou, Yuenan Zhou, Zhongqiu Pan, Qichao Zhang, Yifeng Sheng, Yueqi Lu, Zhiguo Liu, Yixiang Zhang, Guiyun Li, Min Shi, Xuexin Chen, Shuai Zhan

**Affiliations:** 1 Institute of Insect Sciences, Ministry of Agriculture Key Lab of Molecular Biology of Crop Pathogens and Insect Pests, College of Agriculture and Biotechnology, Zhejiang University, 310058 Hangzhou, China; 2 Key Laboratory of Biology of Crop Pathogens and Insects of Zhejiang Province, Zhejiang University, 310058 Hangzhou, China; 3 CAS Key Laboratory of Insect Developmental and Evolutionary Biology, CAS Center for Excellence in Molecular Plant Sciences, Chinese Academy of Sciences, Shanghai, China; 4 CAS Center for Excellence in Biotic Interactions, University of Chinese Academy of Sciences, Beijing, China; 5 State Key Lab of Rice Biology, Zhejiang University, 310058 Hangzhou, China

**Keywords:** Behavioural ecology, Evolutionary genetics, RNAi, Entomology

## Abstract

Parasitoids are ubiquitous in natural ecosystems. Parasitic strategies are highly diverse among parasitoid species, yet their underlying genetic bases are poorly understood. Here, we focus on the divergent adaptation of a specialist and a generalist drosophilid parasitoids. We find that a novel protein (Lar) enables active immune suppression by lysing the host lymph glands, eventually leading to successful parasitism by the generalist. Meanwhile, another novel protein (Warm) contributes to a passive strategy by attaching the laid eggs to the gut and other organs of the host, leading to incomplete encapsulation and helping the specialist escape the host immune response. We find that these diverse parasitic strategies both originated from lateral gene transfer, followed with duplication and specialization, and that they might contribute to the shift in host ranges between parasitoids. Our results increase our understanding of how novel gene functions originate and how they contribute to host adaptation.

## Abstract (web summary)

Parasitism is a widespread evolutionary strategy. A study that spans functional and evolutionary genomics identifies the molecular basis and history underlying two genes that have mediated divergent parasitic strategies (specialist vs generalist) between two sister species of parasitoid wasp.

## Introduction

Parasitism is an ecological process in which two species interact in such a way that one organism, the parasite, lives at the expense of another organism, the host. Parasite–host interaction can be an arms race: whereas the host tends to improve its resistance, the parasite is permanently under selection for increased success^1–3^. Although the counterstrategies that parasites have evolved to subvert host defenses are well characterized, the molecular bases of the adaptations of parasites to their hosts are just beginning to emerge^4–6^.

**Fig. 1:**
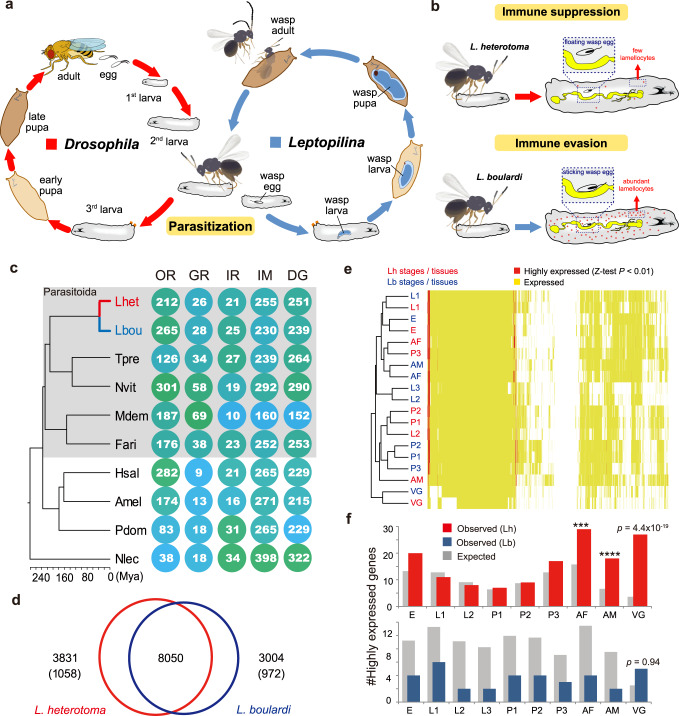
Comparison between Lh and Lb in ecological and genomic contexts. **a** The life cycle of *Leptopilina* (parasitoid) and *Drosophila* (host). Both *L. heterotoma* (Lh) and *L. boulardi* (Lb) are known as larval–pupal parasitoids that deposit their eggs into second instar *Drosophila* larvae; the growing adults emerge from the host pupa shell approximately 3 weeks later. **b** Different strategies by which Lh and Lb avoid host immune responses. **c** Phylogenetic relationships and estimated divergence times among hymenopteran species based on 2401 single-copy orthologous genes. The calibration time was based on a previous study^17^. Right: gene family evolution of representative systems with important roles in parasitoid biology. Circles with a green background indicate gene family expansion, while those with a blue background indicate contraction. OR olfactory receptors, GR gustatory receptors, IR ionotropic receptors, IM immune systems, DG digestive systems, Tpre *T. pretiosum*, Nvit *N. vitripennis*, Mdem *M. demolitor*, Fari *F. arisanus*, Hsal *H. saltator*, Amel *A. mellifera*, Pdom *P. dominula*, Nlec *N. lecontei*. **d** Gene repertoire comparison between Lh and Lb. Numbers in brackets indicate species-specific genes. **e** Heatmap of expression profiles across developmental stages and in venom glands. Each column indicates an orthologous gene pair between Lh and Lb; yellow indicates expression in the corresponding stage (tissue), while red indicates high expression. E eggs, L1 days 1–3 larvae, L2 days 4–9 larvae for Lh while days 4–6 larvae for Lb, L3, days 7–9 larvae for Lb, P1 days 1–3 pupae, P2 days 4–7 pupae, P3 days 8–10 pupae, AF female adults, AM male adults, VG venom glands. **f** Enrichment analysis of highly expressed species-specific genes. Hypergeometric test (higher tail): Bonferroni-adjusted ***P < 0.005 (AF, *P* = 0.0043; AM, *P* = 0.0003), ****P < 0.001. Source data are provided as a Source data file.

Parasitoid wasps are a large group of hymenopteran insects that develop in (endoparasitoids) or on (ectoparasitoids) the bodies of their hosts^7^. They have been widely used as biological control agents for insect pests. In addition, parasitoid wasps are highly diverse and show substantial shifts in hosts or parasitic strategies even between closely related species, making them excellent models to study both rapid speciation and parasite–host coevolution^8–10^. One of the most representative systems is the genus *Leptopilina* (Figitidae), which is well known for parasitism of *Drosophila*. *Leptopilina heterotoma* (Lh) and *L. boulardi* (Lb) are two endoparasitoids that prefer to infect second instar *Drosophila melanogaster* larvae, and adult wasps emerge when the hosts are in the pupal stage^11^ (Fig. 1a). These wasps have similar morphology, size, and generation time (Supplementary Fig. 1) but differ greatly in host range. Lb is specially adapted to *D. melanogaster* and its close relatives, whereas Lh can successfully parasitize a number of species across the *Drosophila* genus, causing these species to be labeled specialist and generalist parasitoid wasps, respectively^12^. Previous studies have revealed that these two wasps have developed different strategies to defeat the immune defense of *Drosophila*^13–16^. Briefly, the numbers of circulating hemocytes and particularly of lamellocytes in the host larvae increase following wasp oviposition^13^. Most lamellocytes are produced by a specialized hematopoietic organ, the lymph gland, which mediates wasp egg encapsulation and can prevent the hatching of wasp larvae^14^. Accumulating evidence suggests that Lh can destroy lamellocytes to inhibit host encapsulation and allow successful parasitism^15^ (Fig. 1b). In contrast to the active immune suppression strategy of Lh, Lb adopts a passive immune evasion strategy, whereby Lb eggs typically become attached to host internal tissues, which provide physical protection against complete encapsulation by host hemocytes^16^ (Fig. 1b). However, the genetic bases underlying these divergent parasitic strategies and how the host shift occurred remain largely unknown.

By combining multi-omics approaches and large-scale in vivo functional studies, we here successfully characterize two key genes that confer the active and passive parasitic strategies of Lh and Lb, respectively, and surprisingly find that they both originated from lateral gene transfer (LGT) and underwent subsequent gene duplication and expression specialization in the venom glands (VGs). We also uncover their adaptive roles in driving the shifts in host range between parasitoids.

## Results

### Overall similar genomic and functional repertoires of specialist and generalist wasps

We first sequenced and de novo assembled the reference genomes of Lh and Lb. The generalist species Lh was fully sequenced with PacBio long reads (Supplementary Table 1), which yielded a 487 Mb genome assembly with high continuity (N50 = 2.18 Mb; Supplementary Table 2). The specialist species Lb was sequenced and assembled with 170-fold Illumina read coverage and paired-end sequencing data from five long-insert libraries (up to 13 Kb; Supplementary Table 3). Although the assembled Lb genome had a lower N50 size (480 Kb) and smaller genome size (324 Mb), both the Lh and Lb genomes showed high completeness based on BUSCO and CEGMA assessments (Supplementary Table 2). The difference in genome sizes between Lh and Lb was likely determined by their repeat contents, as 51.47% of the Lh genome was annotated as repeat content in comparison with a 33.79% ratio in the Lb genome (Supplementary Table 4). The average GC contents of the Lh and Lb genomes were 27 and 26%, respectively, indicating that *Leptopilina* genomes are remarkably AT-rich. Interestingly, unlike the uniform distribution in Lb, the Lh genome shows a secondary peak enriched with scattered genomic windows of remarkably low GC content (16%; Supplementary Fig. 2).

We identified 11,881 and 11,054 protein-coding genes as the official gene sets for Lh and Lb, respectively (Supplementary Table 5). These gene sets were mainly generated by an integrated pipeline, and those retained had evidence from either a full-length transcriptome of pooled developmental stages or high-confidence homology with Insecta genes (see “Methods”). Ortholog analyses across 10 hymenopteran genomes suggested that the genomes of both these parasitoid species maintained a typical hymenopteran gene repertoire (Supplementary Fig. 3). Compared with those of other sequenced insects, the gene numbers of these two *Leptopilina* species are relatively small, largely due to their low proportions of patchy genes that were not vertically inherited along speciation (Supplementary Fig. 3). We manually annotated several representative gene families or pathways that are strongly associated with the biology of parasitoid wasps (Fig. 1c). The genomes of *Leptopilina* encode more olfactory receptor (OR) genes than those of other parasitoids except *Nasonia vitripennis*, while they encode the fewest gustatory receptor (GR) genes. Genes associated with metabolic and immune pathways in *Leptopilina* were shown to be more or less the same as those in other hymenopteran species. Because consistent patterns within a phylogenetic context were not available, we did not gain informative insights into the gene family variations underlying the divergence between *Leptopilina* and other Parasitoida species.

The divergence time between the two *Leptopilina* species was estimated to be approximately 40 Mya, and those between the sublineages of Parasitoida (e.g., Cynipoidea and Chalcidoidea) were estimated to be as distant as hundreds of millions of years (Fig. 1c), consistent with the tremendous diversification of parasitoids^17,18^. Thus we focused on making detailed comparisons within *Leptopilina*. The gene repertoires of Lh and Lb shared 8050 (~75%) orthologous pairs (Fig. 1d) with an average sequence identity of 85.6% at the amino acid (aa) level. We calculated the ratio of synonymous-to-nonsynonymous substitutions (*d*N/*d*S) to characterize the genes and functional modules associated with the divergence between Lh and Lb. Only four pathways were found to have significantly elevated *d*N/*d*S values (*Z*-test *P* < 0.01), including the bacterial secretion system as the most extreme outlier and, unexpectedly, the circadian rhythm pathway (Supplementary Fig. 4). By contrast, we identified 25 pathways presenting *d*N/*d*S values of approximately zero, indicating strong purifying selection. Among these highly conserved pathways between Lh and Lb, we found several sensory systems, including taste transduction as the most conserved pathway and those associated with photoreception and olfaction (Supplementary Fig. 4 and Supplementary Data 1). Interestingly, we noted that the genome of the specialist Lb encoded more chemoreception genes, including ORs, GRs, and ionotropic receptors (IRs), than that of the generalist Lh (Fig. 1c). These genomic signatures did not provide evidence to support a role of environment-sensing modules, e.g., host seeking, in driving the change of host ranges in *Leptopilina*.

We further compared the gene repertoires of Lh and Lb at the level of expression. To profile a dynamic pattern, we sampled developmental stages across the entire lifecycle and subjected each sample to RNAseq (Supplementary Table 6). The VGs were independently sequenced, given their tiny volumes but remarkable roles in the biology of parasitoid wasps^19^. Hierarchical clustering analyses revealed that one-third of the 8050 orthologous genes were expressed across all developmental stages in both species, that most genes were co-expressed in both species at one or more corresponding stages, and that highly expressed genes were co-opted by all developmental stages of both species, except in the VGs (Fig. 1e). By clustering the stages based on their overall expression patterns, we found that the early developmental stages (e.g., the egg and larval periods) of Lh and Lb were intercrossed together, whereas the later stages, particularly the late pupal stage (P3), differed between the species (Fig. 1e and Supplementary Fig. 5). The VGs showed a different expression pattern with all developmental stages and a large amount of species-specific expression, indicative of a unique regulatory system and rapid turnover between species (Fig. 1e and Supplementary Fig. 5). Our results suggest that the lifecycles of Lh and Lb were synchronized at the beginning of development, followed by a shift ahead in the late pupal stage of Lh.

### A particular role of VGs in the divergence between the two species

Of the approximately 3000 non-orthologous genes, Lh and Lb encoded 1058 and 972 species-specific genes, respectively, most of which lacked clear homology outside *Leptopilina* (Supplementary Data 2 and 3). We inferred their potential roles based on the transcriptome and paid close attention to those with evident expression. A total of 740 Lh-specific genes (70%) were expressed in at least one stage, including 88 at significantly high levels, whereas 529 Lb-specific genes (54%) were expressed, and only 12 were highly expressed. Generally, highly expressed genes are skewed toward a high proportion of universal genes^20^. In comparison with the whole set, highly expressed genes were indeed underrepresented in the Lb-specific set across all developmental stages, though not in the VG. By contrast, we did not find evident underrepresentation in the Lh-specific set but were surprised to find significant overrepresentations in the adult stage and particularly in the VGs (Fig. 1f).

Based on the transcriptome analyses, 4274 and 4538 genes were found to be expressed in Lh VGs and Lb VGs, respectively, accounting for approximately one-third of the whole gene set. Of the 50 genes with significantly high expression in Lh VG (*Z*-test, *P* < 0.05), a majority (78%) were exclusively expressed in the late pupal stage (P3) and female adults (AF), when VGs initially formed and developed, respectively (Supplementary Fig. 6). These expression signals were very likely to be contributed by the inclusion of VGs, given that the proportion of genes with exclusively concurrent expression in P3 and AF was extremely low (0.83%) in the whole set (hypergeometric test, false discovery rate (FDR)-adjusted *P* < 0.001; Supplementary Fig. 7). By contrast, many (60%) highly expressed genes in Lb VGs were found to have broad expression across development (Supplementary Fig. 6); none of them were found to have specialized expression in P3 and AF (Supplementary Fig. 7). Furthermore, we found that a majority (62%) of highly expressed genes in the Lh VG were species specific, whereas many of the highly expressed genes in the Lb VG had well-annotated insect homology (Supplementary Figs. 8 and 9). All the described distinct patterns between Lh and Lb concordantly suggest remarkable expression specialization of novel venom genes in the unique adaptation of Lh.

**Fig. 2:**
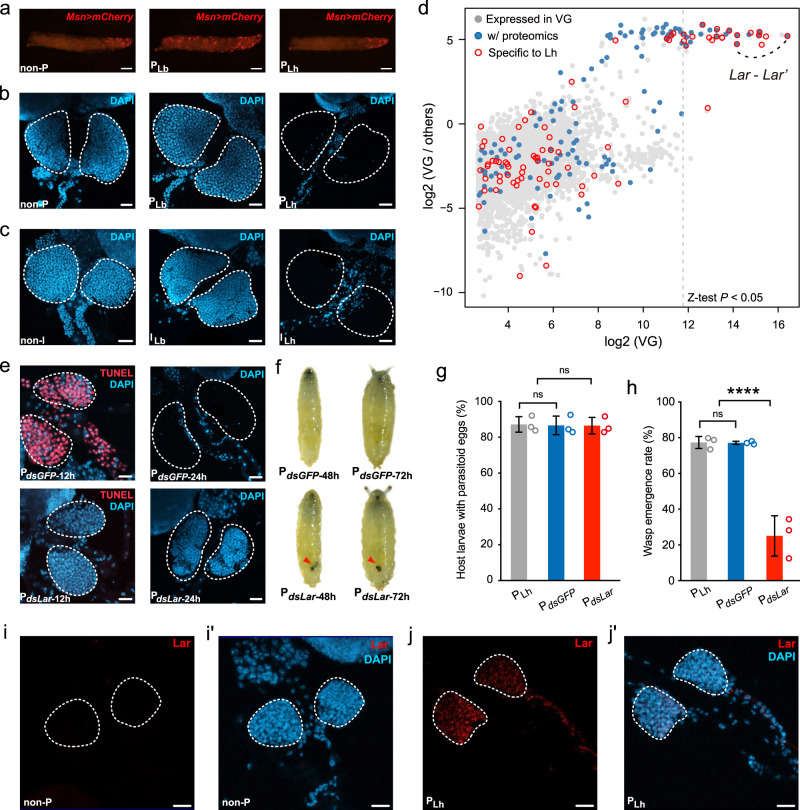
An Lh-specific venom protein enables the suppression of host immunity. **a** Lamellocytes labeled by the Msn>mCherry marker (red)^13^ appeared in vast quantities in the host circulation 48 h after parasitization by Lb (P_Lb_). However, lamellocytes were rarely found in nonparasitized (non-P) and Lh-parasitized (P_Lh_) host larvae (*n* = 3 replicates, at least 50 *Drosophila* larvae were examined for each individual). Scale bars: 1 mm. **b** Lymph glands of nonparasitized and parasitized host larvae 24 h post-infection by Lb and Lh (*n* = 3 replicates, at least 30 lymph glands were examined for each individual). The nuclei were labeled with DAPI (blue). Dashed lines mark the outline of the lymph glands. Scale bars: 20 µm. **c** Lymph glands of *Drosophila* larvae 24 h after injection with venoms derived from Lb (I_Lb_) and Lh (I_Lh_) (*n* = 3 replicates, at least 30 lymph glands were examined for each individual). non-I uninjected control. The nuclei were labeled with DAPI (blue). Scale bars: 20 µm. Dashed lines mark the outline of the lymph glands. **d** Identification of venom proteins (VPs). The expression values in the VGs and the specialized level of expression in the VGs are presented on the *X*-axis and *Y*-axis, respectively. Gray dot, Lh gene with expression in VGs; blue dot, VG-expressed gene with proteome evidence; dot with red border, Lh-specific gene. The pair linked with dashes indicates *Lar* and *Lar’*. See full list in Supplementary Data 4. **e** Lymph glands of host larvae parasitized by *dsGFP*-treated Lh (P_*dsGFP*_) and *dsLar*-treated Lh (P_*dsLar*_). The lymph glands were dissected at 12 and 24 h post-infection (*n* = 3 replicates, at least 50 lymph glands were examined for each individual). Cell apoptosis was stained with TUNEL (red), and nuclei were labeled with DAPI (blue). Dashed lines mark the outline of the lymph glands. Scale bars: 20 µm. **f** *Drosophila* third instar larvae and pupae 48 and 72 h after parasitization by *dsGFP*-treated Lh (P_*dsGFP*_) and *dsLar*-treated Lh (P_*dsLar*_). Red arrowheads represent melanotic encapsulated wasp eggs. **g** Percentages of host larvae exposed to Lh (P_*Lh*_, *n* = 122), *dsGFP*-treated Lh (P_*dsGFP*_, *n* = 170), and *dsLar*-treated Lh (P_*dsLar*_, *n* = 156) that contain wasp eggs. Three biological replicates were performed. Data represent the mean ± SD. Significance was analyzed by two-tailed unpaired Student’s *t* test (P_*dsGFP*_: *P* = 0.9077; P_*dsLar*_: *P* = 0.8857; ns: not significant). **h** Wasp emergence rate after parasitization by Lh (P_*Lh*_, *n* = 582), *dsGFP*-treated Lh (P_*dsGFP*_, *n* = 601), and *dsLar*-treated Lh (P_*dsLar*_, *n* = 693). Three biological replicates were performed and shown as side dots. Data represent the mean ± SD. Significance was analyzed by two-tailed unpaired Student’s *t* test (P_*dsGFP*_: *P* = 0.9761; P_*dsLar*_: *P* = 0.0007; ****P < 0.001; ns: not significant). **i** and **j** Representative images of Lar (red) immunolocalization in nonparasitized (**i**) and parasitized (**j**) host lymph glands 12 h after parasitization by Lh (*n* = 3 replicates, at least 50 lymph glands were examined for each individual). *i’* and *j’* show the merged images of Lar staining (red) and nuclei stained with DAPI (blue). Dashed lines mark the outline of the lymph glands. Scale bars: 20 µm. Source data are provided as a Source data file.

Indeed, the need to adapt to overcoming host immune responses and changing host ranges drives parasitoid venom repertoires to evolve quickly and to acquire novel functions^21^. In Lh and Lb, venom is produced in the VG, mostly packaged in virus-like-particles (VLPs), and stored in the venom reservoir (VR)^22,23^. These VLPs are then injected along with the wasp egg during oviposition. We further studied the relevant functions of Lh and Lb venoms, which participate in reproductive success. Similar to previous reports^13^, we confirmed the absence of lamellocytes in unchallenged *D. melanogaster* larvae (Fig. 2a). In contrast, large quantities of lamellocytes were produced 48 h after attack by Lb (Fig. 2a). As expected, oviposition by Lh led to no induction of lamellocytes in parasitized larvae, which appeared comparable to unchallenged *Drosophila* larvae (Fig. 2a). It is well known that lamellocytes are mostly produced by the lymph gland (the hematopoietic organ) to encapsulate large foreign bodies^14^. Thus we dissected the lymph glands from host larvae that were parasitized by the two *Leptopilina* wasp species. We found that Lh parasitization caused rapid lysis of the host lymph gland 24 h after wasp egg laying, while the lymph gland was intact in Lb-parasitized larvae 24 h post infection (Fig. 2b). To determine whether the Lh venom was responsible for the lysis of the host lymph gland, we isolated venom fluid from the VRs of the two wasps and injected each of them directly into a host. After 24 h, we found that the injection of Lh venom caused loss of the host lymph gland, while the venom of Lb showed no effect (Fig. 2c). In combination, these results indicate that the venom of Lh has evolved much greater virulence than that of Lb and subsequently leads to a nearly complete lack of circulating lamellocytes in the attacked hosts by inducing apoptosis in the lymph gland (Fig. 2e).

### A novel venom protein allows Lh to overcome host immunity

Thus the venom of Lh is very likely to be responsible for its immune suppression strategy. Since the genes expressed in the VG are not necessarily translated and secreted into the reservoir, we further generated proteomes from both Lh and Lb venom samples and integrated them with the venom transcriptomes to characterize reliable venom proteins. As expected, only a small set of VG-expressed genes were found to have solid proteome evidence (Fig. 2d), and these were determined to be venom protein-coding genes (VPs; see “Methods” and Supplementary Data 4). Of all the identified VPs (135) in Lh, a pair of closely related paralogs (LhOGS04147 and LhOGS20123) attracted our attention due to their extremely high expression in the venom (the first and sixth highest of all VPs, respectively), their marked expression specialization, the signature of rapid evolution (the between-sample *d*N/*d*S is 0.8392, higher than 99.9% of Lh–Lb orthologs; Supplementary Fig. 10), and the absence of homologs outside Lh (Fig. 2d).

We did not characterize any known functions or conserved domains for these two genes (see “Methods”), while the three-dimensional (3D) modeling predicted a hydrolase-like structure (Supplementary Fig. 11). We explored their potential functions in vivo via RNA interference (RNAi) experiments. Despite a lower expression value, LhOGS20123 showed a more marked signature than LhOGS04147 in the proteome (Supplementary Data 4). The specialized expression of LhOGS20123 in VGs was also confirmed by immunohistochemistry and western blot analysis (Supplementary Fig. 12). Double-stranded RNAs (dsRNA) of *LhOGS20123* were injected into fifth instar larvae of Lh, and quantitative real-time PCR (qRT-PCR) showed that gene expression decreased by 85% in emerged wasp adults (Supplementary Fig. 13a). In comparison to *dsGFP*-injected wasps, we found a significant suppression of apoptosis in the host lymph gland and a correspondingly large population of lamellocytes in host larvae when parasitized by *LhOGS20123*-knockdown wasps (Fig. 2e, f and Supplementary Fig. 14). Knocking down this gene in female Lh also largely resulted in the predomination of melanotic capsules in infected hosts and widespread encapsulation of wasp eggs, which was rarely observed in the hosts parasitized by *dsGFP*-injected Lh (Fig. 2f and Supplementary Fig. 14). Given its substantial role in the lysis of lymph glands, we named this gene *Lar* (lymph gland apoptosis-related protein). We then performed an assay to detect whether parasitic efficiency was impaired in *dsLar*-injected female wasps due to a lack of the ability to repress the host encapsulation response. We found that the oviposition ability of Lh was unaffected by *dsLar* treatment (Fig. 2g), whereas the parasitism rate was dramatically decreased, with only 27% of wasp offspring successfully emerging in *Drosophila* hosts compared to 77% when parasitized by either normal Lh or *dsGFP*-treated Lh females (Fig. 2h). The results of antibody staining showed that Lar was readily detected in host lymph glands 12 h after Lh parasitization but not in other tested tissues (Fig. 2i, j’ and Supplementary Fig. 15). Though it is still not clear how Lar is specifically delivered into the host lymph gland in such a short time, it is possible that VLPs that are present in the Lh female venom apparatus may help to facilitate its entrance. Interestingly, VLPs have been renamed mixed strategy extracellular vesicles (MSEVs) or venosomes because there is no solid evidence of a viral origin^22,24^. It will be both interesting and urgently necessary to identify the key elements that are in charge of delivering VPs into specific host tissues rapidly and precisely.

Unlike *Lar*, its paralog, *LhOGS04147* (referred to as *Lar’*), was not effective with respect to suppressing host encapsulation response. As with previous approaches, silencing *Lar’* did not rescue the parasitization-induced apoptosis of the host lymph gland or lead to the development of obviously melanotic capsules in parasitized *Drosophila* larvae (Supplementary Fig. 13). Most importantly, the parasitism rate and wasp offspring emergence rate were not changed when *Drosophila* larvae were parasitized by *Lar’*-silenced Lh female wasps compared to control wasps with *dsGFP* treatment (Supplementary Fig. 13). We additionally knocked down the other seven genes out of the ten VP genes of highest expression (i.e., *LhOGS06609*, *LhOGS10118*, *LhOGS01638*, *LhOGS01639*, *LhOGS01180*, *LhOGS02019*, and *LhOGS00546*; see Supplementary Data 4) and two VP genes annotated with the highest peptide number (i.e., *LhOGS20077* and *LhOGS08557*; see Supplementary Data 4). Although qRT-PCR showed that the expression levels of these nine genes were all significantly reduced upon the injection of corresponding dsRNAs (Supplementary Fig. 16a), the parasitism rate was not found impacted in the host larvae parasitized by eight *dsRNA*-treated lines of wasps, with the only exception in the *dsLhOGS06609*-treated line (Supplementary Fig. 16b). In comparison to the control, the parasitism rate was significantly but marginally reduced upon the parasitization of *dsLhOGS06609*-treated Lh (Supplementary Fig. 16b). However, we found that knockdown of *LhOGS06609* did not relieve the apoptosis of the host lymph glands (Supplementary Fig. 16c).

Taken together, these results suggest that Lar plays an important and unique role in provoking cell death in the lymph gland and hence suppress the host encapsulation response, leading to successful parasitism, and that some other VPs have minor effect on the parasitic efficiency of Lh.

### Recruitment of novel VPs arose from LGT followed by rapid evolution

We next traced the origin and genomic evolution of this functionally important Lh-specific gene. Microbial contamination may cause false positive results when identifying species-specific genes. However, *Lar* was unlikely to be identified as part of a chimeric scaffold, given that the genome of Lh was continuously assembled using long reads. In addition, two full-length transcripts revealed that *Lar* bears an 8.6 kb intron (Supplementary Fig. 17), whereas bacterial or phage genes are generally intronless^25,26^. In fact, the complete structure of *Lar* was fully embedded in the first intron of another reverse-strand-located gene, *RRP8* (Supplementary Fig. 17). *RRP8* is widespread across insect species and occurs in a short microsynteny block within *Leptopilina* (Lh, Lb, and *L. clavipes*, Lc; Supplementary Fig. 18)^27^. Despite its conserved exon–intron structure, the first intron of *RRP8* showed diverse architectures among the three *Leptopilina* species (Supplementary Fig. 17): (1) the copy in Lc was the simplest; (2) the copy in Lb shared two homologous segments with that in Lc, but one was disrupted by both a segmental duplication and an unfilled gap (see the figure legend); (3) the copy in Lh was replaced with the complete structure of *Lar* and its flanking, highly repeated, highly AT-rich (91%) sequences. On the other hand, the paralog of *Lar*, *Lar’*, which has 73% sequence identity at the nucleotide level (Supplementary Fig. 10), was located on a different scaffold. The orthologs of the two genes adjacent to *Lar’* were located on different scaffolds in Lb and Lc, but they were adjacent in a more distantly related wasp species (*N. vitripennis*, Chalcidoidea) (Supplementary Fig. 19); that is, the local synteny was of ancient origin (prior to the divergence between the two lineages of parasitoids, 160 Mya (Fig. 1c)) and then was broken during recent evolution within *Leptopilina*. *Lar’* showed both the same exon–intron structure and the same expression pattern as *Lar* (Supplementary Fig. 10) and, more importantly, was also surrounded by dispersed repetitive sequence blocks. Repetitive sequences increase the potential for duplication, deletion, and transfer across genomes. Indeed, Lh greatly differed from Lb in having a larger genome size and a unique enrichment of AT-rich genomic regions (Supplementary Fig. 2), suggesting more rapid turnover in the genome of Lh (Supplementary Table 4). Hence, we infer that *Lar* and *Lar’* originated recently from a common ancestor and were translocated into two separate repeat-rich genomic loci.

**Fig. 3:**
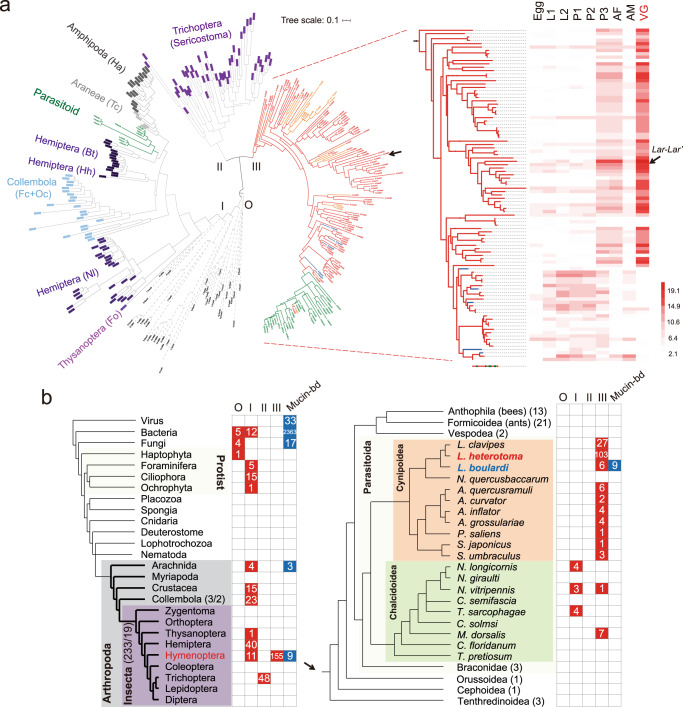
Genomic origin and evolution of *Lar*. **a** Phylogenetic tree of all identified *Lar* homologs. Genes from Lh, Lb, Lc, and other parasitoid species are shown by red, blue, orange, and green branches, respectively. Nodes with colored backgrounds indicate genes from other animal species, while those in gray indicate microorganism genes. Fo *Frankliniella occidentalis*, Nl *Nilaparvata lugens*, Fc *Folsomia candida*, Oc *Orchesella cincta*, Hh *Halyomorpha halys*, Bt *Bemisia tabaci*, Tc *Trichonephila clavipes*, Ha *Hyalella azteca*. A local phylogeny containing the Lh and Lb genes is independently shown with an expression heatmap. The stage names correspond to those in Fig. 1. **b** Distribution of identified genes in each taxon group. The phylogenetic topology was based on previous studies^97,98^. Note that only representative taxa are listed in the tree; for insects, a total of 233 species from 20 orders were investigated (see “Methods”), while all the animal taxa listed in the NR database were subject to screening. Information within Hymenoptera (51 species) is shown with details in the context of hypothetical phylogeny^17,99–101^ on the right. The numbers in brackets indicate the number of species in the corresponding family; the latter number after a slash indicates the number of species presenting homologs. O, I, II, and III correspond to the four main clades as shown in **a**. See detailed information of mucin-bd-related statistics (in blue) in later sections.

Where did the ancestral copies of these two novel genes come from? We searched for putative homologs in the NCBI nonredundant (NR) protein database and found only a few hypothetical proteins with low aa identities (20–30%). Beyond their scattered distribution in a few arthropods, all these hits belonged to microbial and protist species (Fig. 3a). This absence in all metazoans outside the arthropods indicates that these arthropod genes originated from one or more LGT events. Their patchy distribution across arthropods raised the possibility that homology might have been overlooked by protein-coding gene prediction, which might miss genes due to either degraded structures or a deficiency of strong homology. To characterize the traces of *Lar* as completely as possible, we directly searched for its potential homologs in the genomic sequences of Lh and Lb (see “Methods”). A total of 94 and 6 genomic loci were identified in the Lh and Lb genomes, respectively (Supplementary Data 5). Indeed, most of these loci were not predicted in our official gene sets due to a lack of sufficient evidence, although they shared a relatively conserved blocks with G1-motif signature (Supplementary Fig. 20)^22^. Despite the uncertainty that these sequences were true protein-coding genes, the presence of such widespread G1 motifs suggests a more ancient origin and broader distribution than we expected. We further performed the same iterative searches to determine whether these motifs were underrepresented across the deposited gene sets of other species. Out of the 236 Hexapoda genomes we examined (including three *Leptopilina* species; see “Methods”), two Collembola species and 19 insect species from four orders were found to harbor at least one gene encoding the G1 motif (Fig. 3a and Supplementary Fig. 21). 3D modeling further predicted a Lar-like structure between distantly related sequences (Supplementary Fig. 11).

We reconstructed a maximum-likelihood (ML) phylogeny using all the recovered sequences and representative hits from NR, with prokaryotic sequences as outgroups. The phylogeny resolved three main clades (Fig. 3b), of which one (clade III) contained most of the wasp sequences except 11 sequences from three Chalcidoidea species, one (clade II) contained all the sequences from a Trichoptera species, and one (clade I) contained all the remaining arthropod sequences. Of the 269 recovered genes, 58% were from parasitoid wasp species, with only a minor proportion, all from Chalcidoidea, being placed together with the other Arthropod ones in clade I (Fig. 3). Interestingly, the Chalcidoidea parasitoid, *N. vitripennis*, is the only species that simultaneously retains the copies of clades I and III. However, phylogenetic analysis separated these copies by non-Metazoan copies (Supplementary Fig. 21). The deep split between these clades and the phylogenetic conflict between gene and species tree topologies both strongly suggest that the scattered presence of these Arthropod homologs were likely to originate from multiple, independent LGTs and experience distinct fates. LGTs of clade I probably has an ancient origin of transferring to the common ancestor of Arthropods and underwent massive losses independently, whereas the clade III, which arose *Lar*, likely took place prior to the divergence between Cynipoidea and Chalcidoidea wasps (>200 Mya; Fig. 1c) and was largely retained in Cynipoidea lineage (Fig. 3b). Due to the substantial divergence, we cannot pinpoint a precise donor as the source of these foreign DNA.

According to the phylogeny within clade III, we found that the Lh genes with specialized expression in venom were remarkably separated from those with broad expression and that the sequences from all other parasitoid wasps were clustered with the latter (Fig. 3a and Supplementary Fig. 22). A possible explanation is that the LGT underwent massive duplication along with its regulatory elements as a whole; evolved variations might give rise to changes in expression pattern and hence result in a functional split. The sublineage with VG specialization was retained or even further expanded in Lh but completely lost in all other species, whereas the sublineage with broad expression had the potential for neofunctionalization and hence was modestly retained in most Cynipoidea wasps. Due to the lack of definitive support in the phylogeny, it was difficult to infer the chronological order of the duplication and functional split. From the expanded group of genes with specialized VG expression, we chose *LhOGS20047*, the most highly expressed member after *Lar’* and *Lar* (Supplementary Data 5), to test whether it had a similar function to that of *Lar*. Despite the proteome evidence, RNAi of this gene, similar to that of *Lar’*, did not relieve the lysis of the host lymph gland or affect the rate of parasitism and wasp offspring emergence (Supplementary Fig. 13). Thus it seems that most members of the expanded gene family did not evolve with venom-related functions despite their specialized expression in the VGs, which might partially explain why they were completely lost in other species. Further specific functional studies would help understand how the unique sequence evolution in Lar contributes to the functionalization regarding immune suppression.

### Acquisition of a set of mucin-binding domain-containing genes helps Lb evade host immunity

**Fig. 4:**
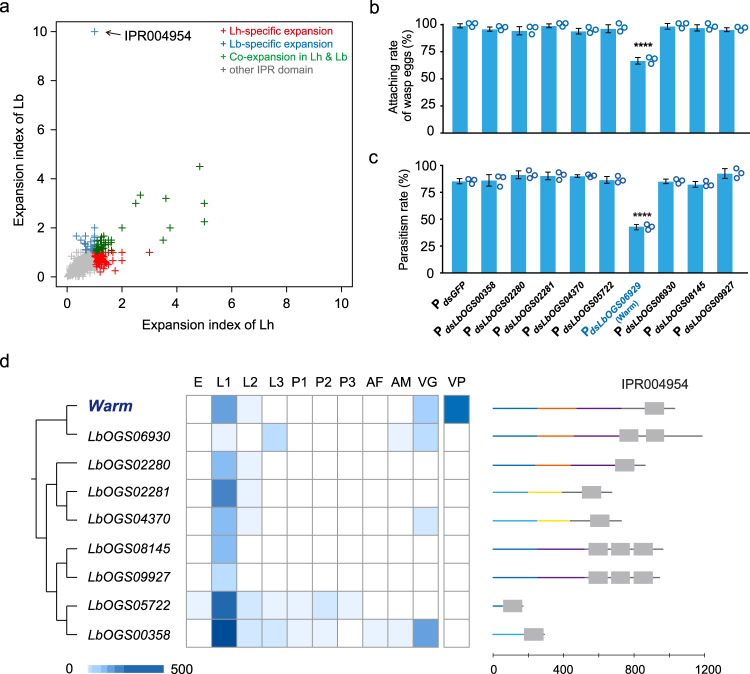
Unique expansion of mucin-binding domain-containing genes in Lb. **a** Expansion and contraction of all annotated InterPro domains (IPRs) between the Lh and Lb genomes. The expansion index indicates the ratio of identified IPRs in the corresponding species to the highest number in all other hymenopteran species. See detailed information in Supplementary Data 6. **b** The attachment rate of wasp eggs in host larvae that were parasitized by Lb depleted of each of the nine mucin-binding domain-containing genes via RNAi treatment. *dsGFP*-treated Lb (P_*dsGFP*_) was used as a control. Left to right: *n* = 81, 99, 99, 99, 86, 75, 76, 83, 102, and 86 biologically independent host larvae. Three biological replicates were performed and shown as side dots. Data represent the mean ± SD. Significance was determined by two-tailed unpaired Student’s *t* test (P_*dsLbOGS00358*_: *P* = 0.1196; P_*dsLbOGS02280*_: *P* = 0.1387; P_*dsLbOGS02281*_: *P* = 0.9661; P_*dsLbOGS04370*_: *P* = 0.0569; P_*dsLbOGS05722*_: *P* = 0.3264; P_*dsLbOGS06929*_: *P* = 0.0001; P_*dsLbOGS06930*_: *P* = 0.8246; P_*dsLbOGS08145*_: *P* = 0.3972; P_*dsLbOGS09927*_: *P* = 0.077; ****P < 0.001). **c** Parasitism rates in *D. melanogaster* host larvae attacked by the above dsRNA-treated Lb wasps. Left to right: *n* = 76, 62, 78, 86, 99, 83, 58, 79, 152, and 86 biologically independent host larvae. Three biological replicates were performed and shown as side dots. Data represent the mean ± SD. Significance was determined by two-tailed unpaired Student’s *t* test (P_*dsLbOGS00358*_: *P* = 0.8317; P_*dsLbOGS02280*_: *P* = 0.0791; P_*dsLbOGS02281*_: *P* = 0.1182; P_*dsLbOGS04370*_: *P* = 0.0365; P_*dsLbOGS05722*_: *P* = 0.6201; P_*dsLbOGS06929*_: *P* = 2.9e-5; P_*dsLbOGS06930*_: *P* = 0.927; P_*dsLbOGS08145*_: *P* = 0.2255; P_*dsLbOGS09927*_: *P* = 0.0721; ****P < 0.001). **d** Expression heatmap of nine mucin-bd-containing genes. Samples correspond to those in Fig. 1. Gene architectures are shown on the right. Lines represent coding sequences; those in the same color indicate homologous segments across genes (peptide identity to *Warm* >50%), while those in lighter color indicate potential homologous segments of poorer identity (<50% to *Warm*). Gray boxes represent IPR004954. Source data are provided as a Source data file.

It is intriguing that Lh exclusively recruited an LGT to perform a novel venom function that confers a new strategy for parasitic success. We next explored whether the specialist, Lb, had evolved alternative strategies for parasitic success through the acquisition of novel genes. The massive expansion of *Lar* across the genome suggests a particular role for gene duplication associated with the acquisition of novel genes. A small set (126) of Lb-specific genes were multiple copy, and we noticed that 11 of these genes formed the largest paralogous group (Supplementary Data 3) and that 9 of them encoded the putative mucin-binding domain (mucin-bd, IPR004954). This domain was also the most extreme outlier in an expansion/contraction analysis across all annotated InterPro domains between Lh/Lb and other hymenopterans; it was present in nine Lb genes but absent in all ten of the other hymenopteran gene sets we examined (Fig. 4a and Supplementary Data 6). The mucin-bd was documented in 260 sequences from 85 species in the Pfam database (PF03272) and in 2444 sequences from 553 species in the InterPro database (as of Feb. 29, 2020) (Supplementary Data 7 and 8). Of these sequences, 97.9% were bacterial, 2% were from viruses or fungi, and only 3 sequences belonged to a metazoan species (*Parasteatoda tepidariorum*, Arachnida) (Supplementary Fig. 23). Domain alignment did not show any homologous segments (*E* < 1e−5) between the nine Lb genes and those from metazoan or fungi species. Correspondingly, BLAST searches against NR identified only bacterial hits, mainly as viral enhancing proteins or hypothetical proteins, with modest conservation, for these nine genes (as of Feb. 29, 2020; Fig. 3b and Supplementary Table 7). Unlike the scattered distribution of *Lar* across arthropods, the above patterns suggest a more straightforward LGT that resulted in the widespread presence of the mucin-bd domain across the Lb genome. Correspondingly, several genera of bacteria enriched with mucin-bd domains were able to be recovered at the genus level from the microbiota sequencing of Lb guts and female whole bodies (Supplementary Fig. 23 and Supplementary Data 9).

We hypothesized that this bacterial domain was recruited as a parasitic strategy for Lb to circumvent host immunity. This microbial domain represents a putative binding domain for the substrates of enhancin, which is involved in the disruption of the peritrophic membrane and the fusion of nucleocapsids with midgut cells^28^. To test this hypothesis, we used the RNAi technique to knock down each of the nine mucin-bd domain genes individually. The qRT-PCR results showed that the expression levels of these nine genes were significantly reduced in Lb adults after injection of the corresponding dsRNA into fifth instar Lb larvae as previously described (Supplementary Fig. 24). We next used 3-day-old dsRNA-treated Lb female wasps to parasitize *Drosophila* host larvae, with *dsGFP*-injected wasps as a control. Similar to our previous findings, >95% of wasp eggs were gradually attached to the host internal tissues (mainly host gut) by 4 h after parasitization by *dsGFP*-injected Lb (Fig. 4b); this strategy provides passive, physical protection against complete encapsulation by host hemocytes, and thus the wasp larvae can escape from the attachment site during hatching. The attachment rate of Lb eggs showed a significant reduction in *Drosophila* hosts when parasitized by LbOGS06929 knockdown female wasps (Fig. 4b). Thus LbOGS06929 was named Warm (wasp egg adhesion-related protein with a mucin-bd domain). However, the attachment rates of Lb eggs laid by female wasps with silencing of the other eight mucin-bd domain-containing genes were not impaired and were comparable to those of the *dsGFP*-treated control (Fig. 4b). Meanwhile, a significantly decreased parasitism rate was found when the hosts were parasitized by *dsWarm*-injected Lb but not by other treated wasps (Fig. 4c). Examining the Lb venom proteome, we found that Warm was also a venom protein that could be injected into the host body along with the wasp eggs (Fig. 4d). In contrast to the findings that injected venoms often induce cytotoxic effects on hemocytes, our results suggest that Warm is responsible for the adhesion of parasitoid eggs to host tissues and helps Lb wasps adopt an immune evasion strategy to overcome the host encapsulation reaction for successful parasitism.

Why does only one of these mucin-bd-containing genes show functional importance? Similar to previously documented genes in Pfam, the nine mucin-bd-containing genes in Lb had diverse domain architectures and were highly variable (Fig. 4d), indicating rapid evolution. Two genes (LbOGS00358 and LbOGS05722) showed substantial sequence degradation (Fig. 4d). Of the seven remaining genes, five showed specialized expression in the early larval period (L1), and two others, including *Warm*, had strong expression in the venom, despite their higher values in L1 (Fig. 4d). However, only *Warm* was present in the proteome. Two of the five specialized genes, LbOGS02281 and LbOGS04370, retained the most complete architecture compared to their bacterial homologs, with orders of magnitude higher level of possibility than those of the other genes (Supplementary Table 7). Thus we propose a scenario in which the LGT was basally expressed specifically in early larvae and then underwent gene co-option by the venom, resulting in novel venom functions.

### The diverse parasitic strategies between Lh and Lb may contribute to the shift in host range

**Fig. 5:**
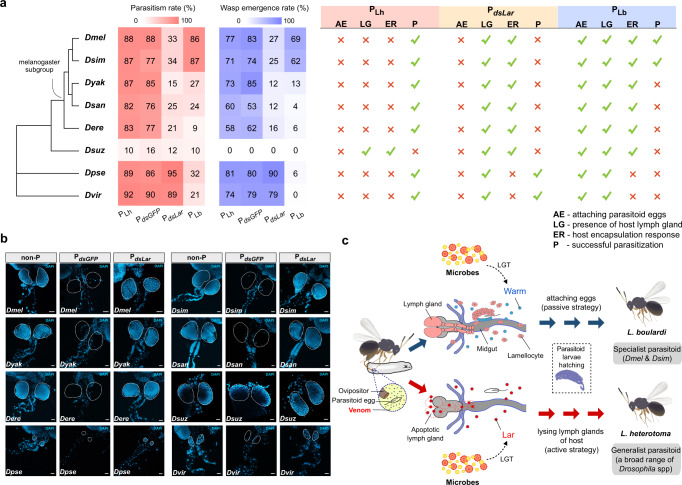
The adaptive roles of Lar and Warm in driving the shift in host range. **a** Parasitism rates and wasp emergence rates in eight *Drosophila* species parasitized by *L. heterotoma* (P_*Lh*_), *dsGFP*-treated *L. heterotoma* (P_*dsGFP*_), *dsLar*-treated *L. heterotoma* (P_*dsLar*_), and *L. boulardi* (P_*Lb*_). The fate of attaching wasp eggs, the presence of host lymph gland 24-h post-infection, and the encapsulation of wasp eggs were also shown on the right. The green checkmark represents present or yes, and the red X represents absent or no. **b** Lymph glands of nonparasitized *Drosophila* species (non-P) and of the host larvae that were parasitized by *L. heterotoma* (P_*Lh*_) and *dsLar*-treated *L. heterotoma* (P_*dsLar*_) (*n* = 3 replicates, at least 50 lymph glands were examined for each individual). The nuclei were labeled with DAPI (blue). Dashed lines mark the outline of the lymph glands. Scale bars: 20 µm. **c** The proposed model of how *Leptopilina* wasps have evolved diverse parasitic strategies to combat with their *Drosophila* hosts, leading to a specialist parasitoid (Lb) and a generalist parasitoid (Lh). Dmel *D. melanogaster*, Dsim *D. simulans*, Dyak *D. yakuba*, Dsan *D. santomea*, Dere *D. erecta*, Dsuz *D. suzukii*, Dpse *D. pseudoobscura*, Dvir *D. virilis*.

Lh and Lb are closely sympatric species. Lb is an obligate parasitoid of *D. melanogaster*, whereas Lh is a generalist parasitoid with a wide host range within Drosophilidae^12^. We have characterized *Lar* and *Warm* that enable Lh and Lb to overcome the immune response of *D. melanogaster* in dramatically different ways. We next tried to address whether these diverse parasitic strategies might contribute to their different host ranges. We tested the parasitic efficiencies of Lh and Lb in eight *Drosophila* species, including five species in the melanogaster subgroup (*D. melanogaster*, *D. simulans*, *D. yakuba*, *D. santomea*, and *D. erecta*) and three species outside the melanogaster subgroup (*D. suzukii*, *D. pseudoobscura*, and *D. virilis*). We found that both Lh and Lb female wasps oviposited in the larvae of all eight species, regardless of whether parasitoid development succeeded. As expected, Lh could successfully parasitize almost all the species we tested, with a parasitism rate ranging from 82 to 92% and an offspring emergence rate of >58% (Fig. 5a). The only exception was *D. suzukii*, in which Lh was unable to complete development (Fig. 5a), consistent with previously described results^12,29^. As a specialist, Lb showed high parasitism rates (86 and 87%, respectively) and offspring emergence rates (69 and 62%, respectively) only in *D. melanogaster* and its close relative, *D. simulans*. Lb failed to develop in the other six *Drosophila* species, resulting in few Lb wasps, if any, emerging from these hosts (Fig. 5a).

Host immune responses can result in encapsulation of parasitoid eggs, which leads to the death of the parasitoid offspring^14^. Fly larvae generate the lamellocytes used for encapsulation after wasp infection. We demonstrated that Lh used an active strategy to suppress the host immune response by inducing apoptosis in the lymph glands of parasitized *D. melanogaster*. Accordingly, we found that the lymph glands disappeared in the Lh-parasitized larvae of all the other *Drosophila* species at 24 h after parasitization, except *D. suzukii* (Fig. 5b). We next used RNAi to test the role of Lar in the lysis of lymph glands in these other *Drosophila* species. Knocking down *Lar* resulted in a significant rescue of the loss of lymph glands, which subsequently resulted in strong encapsulation responses in parasitized species of the melanogaster subgroup (Fig. 5a, b). Therefore, silencing *Lar* in the VG resulted in a failure to parasitize these *Drosophila* hosts. Interestingly, no encapsulation responses were found in *D. pseudoobscura* and *D. virilis*, although their lymph glands were intact after parasitization by *dsLar*-treated Lh (Fig. 5a, b). Thus the high parasitic efficiency in *D. pseudoobscura* and *D. virilis* is probably because the lymph glands lacked the ability to differentiate lamellocytes. Indeed, we found that the lymph glands in *D. pseudoobscura* and *D. virilis* were small compared to those in the other species (Fig. 5b). Some studies have revealed that most *Drosophila* species outside the *melanogaster* subgroup usually do not differentiate lamellocytes upon wasp infection^30^. Collectively, these results suggest an evolutionary scenario in which the emergence of encapsulation responses in a common ancestor of *melanogaster* subgroup flies might drive novel adaptive strategies in which the effective suppression of host cellular immune responses by Lh might contribute to its colonization of a wide range of hosts.

In contrast to Lh, the specialist species Lb has evolved a relatively passive strategy to parasitize *D. melanogaster*, i.e., through attaching the eggs to host tissues to escape encapsulation with the help of Warm. Our assay showed that both the parasitism rates and offspring emergence rates of Lb were as high as those of Lh when parasitizing *D. melanogaster* and *D. simulans* (Fig. 5a). When Lb parasitized other *Drosophila* species, we found that the encapsulation responses of the hosts were widely activated, except those of *D. pseudoobscura* and *D. virilis*, and that the eggs of Lb also attached to host tissues in all species (Fig. 5a). It is apparent that other factors hindered the further development of Lb wasp eggs in these species; these factors may be related to physiological unsuitability^31^. These observations suggest that the passive immune evasion strategy might enable parasitoids to adapt to a specialized host as successfully as the aggressive strategy but might not support extending the host range.

## Discussion

Parasitoid wasps are highly ubiquitous and diverse on the planet, with estimates of >150,000 species including almost 20% of all insect species^7^. Evolutionary arms race promotes the wasp to evolve numerous strategies to circumvent the host immune defenses^32,33^. Previous studies have made significant progresses in characterizing parasitic strategies at the organismal and cellular levels^34^, yet much remains to be understood of the genetic bases. In this study, we performed comprehensive comparisons in genomes, transcriptome, and proteome that enabled us to identify *Lar* and *Warm* as the most extremely outlier underlying the genetic differences between Lh and Lb. We verified their functions in vivo via RNAi experiments, which clearly showed that *Lar* and *Warm* are responsible for an active immune suppression strategy of Lh and a passive immune evasion strategy of Lb, respectively. Specifically, *Lar* triggers the lysis of the *Drosophila* lymph glands, eventually leading to the inhibition of host encapsulation response, whereas *Warm* contributes to the attachment of wasp eggs to the internal organs of the host, providing physical protection against the complete encapsulation that helps parasites escape from the host cellular immune response.

Venomous animals use venoms to prey capture and defend or manipulate their hosts in numerous ways. Venoms are generally complex cocktails of bioactive compounds and under highly rapid evolution^35^. The venoms of both Lh and Lb are packaged in MSEVs or venosomes, which are responsible for the delivery of venom proteins^22,24^. The Lh VP, Lar, prevents the release of lamellocytes by apoptosing the host lymph glands, leading to a near complete lack of circulating lamellocytes in parasitized hosts (Fig. 2e). By contrast, the Lb venom does not cause the lysis of host lymph glands, resulting in the massive differentiation of lamellocytes in parasitized hosts (Fig. 2a). In response to the encapsulation by host hemocytes, the Lb eggs are attached to host tissues with the help of the Lb VP, Warm (Fig. 4b). These findings are likely to be exploited more widely in other parasite–host systems, since many parasites that cause serious diseases in humans also secrete functional proteins in EVs that contribute to the suppression of host immune responses and the parasite adherence to host tissues^36–38^.

In addition, we found that the transcripts of Lh VGs were dominated by genes specific to the species and expressed exclusively in VGs (like *Lar*). Such a pattern would allow rapid turnover of venom repertoires and acquisition of novel functions, supporting the expectation that the Lh venom is more virulent than that of Lb^12^. LGT is a potentially important source of evolutionary innovation^39–41^. Examples of LGT in insects have been found to be involved in the aspects of detoxification, protection, and metabolism^42–44^. LGTs were also documented in parasitic microbial eukaryotes^45–47^. As one of a handful of examples in parasitic insects, an LGT of Glycoside hydrolase family 19 (GH19) has been reported in the VG of chalcidoid wasps, but whether it involves in successful parasitization was unclear^48^. Our study presents two novel LGTs, *Lar* and *Warm*, which directly confer the active and passive parasitic strategies, respectively, providing solid evidence for the role of LGTs in adaptive evolution. Remarkably, the extraordinary evolutionary paths of *Lar* and *Warm* add to our understanding of evolution following LGT. Both LGTs were found associated with subsequent regulatory element evolution that resulted in changes in expression profiles, i.e., the expression specialization of *Lar* in VGs and the shift from larvae to venom of *Warm* (Figs. 3a and 4d). The co-option of single-copy genes has shown an important role in the evolution of new gene functions in chalcidoid wasps^21^. Our findings further highlight the importance of gene co-option in shaping the functional repertoire of VGs. Previous studies reported that the presence of introns increases gene expression in eukaryotes^49^ and that gene duplication might provide new enhancer modules through mutations that alter the expression pattern and result in neofunctionalization^50^. Thus we hypothesize that intron gain and massive duplication (Supplementary Figs. 17 and 20) might promote changes in expression pattern of the ancestral copies of *Lar* and *Warm* that eventually helps a copy of them become functional, while the other non-functional copies are becoming or have been silenced.

There is also considerable interest in addressing the shift in host range and host preference, which addresses fundamental evolutionary questions such as sympatric speciation and parasite–host and plant–herbivore coevolution^51,52^. In general, the shift in host range can be influenced by many ecological and physiological indicators, including host location and host adaptation (e.g., host physiological suitability or host immune responses)^53^. In the well-studied system of *Drosophila*, taste perception, encoded by two odorant-binding proteins, was shown to drive the host specialization of *D. sechellia* from its generalist relatives^54^. Parasitoid wasps show extremely diverse host ranges across species^55^, but related studies have been mainly done in the system of *Nasonia*, including the annotation of genes involved in host finding and the identification of a 16-Mb major locus underlying the host preference between the generalist *N. vitripennis* and the specialist *N. giraulti*^10,56,57^. In our research system, Lh is a generalist parasitoid with a wide host range within *Drosophilidae*, whereas Lb is a specialist that often parasitizes flies in the *D. melanogaster*/*D. simulans* clade in nature. We unexpectedly found that the genome of the specialist Lb encodes more chemoreception genes, including ORs, GRs, and IRs, than that of the generalist Lh (Fig. 1c). We also showed that the genetic modules related to environment perception are remarkably conserved between Lh and Lb (Supplementary Fig. 4). These genome-wide signatures do not suggest a role of host-seeking phase in driving the shift in host range between Lh and Lb. Instead, our results suggest that the Lh-specific gene, *Lar*, contributed to the apoptosis in the host lymph glands that eventually allowed Lh to colonize a wide range of hosts, while the mucin-bd-containing gene specific to Lb, *Warm*, contributed to the attachment of wasp eggs to host internal tissues, but not to extend host ranges (Fig. 5c). Thus the host adaptation phase was likely to play an important role in the evolution of host range in parasitoid wasps.

Due to the challenge in adapting to different hosts, generalism was ever considered as a transitory evolutionary event or evolutionary improbability^58^. The genome-wide investigation of *Lar* homologs suggests that the immune suppressive strategy, adopted by the generalist and contributed by *Lar*, was inclined to be deprecated by most related species. Despite the widespread presence in cynipoid parasitoids, all putative homologs of *Lar* outside Lh were evidently diverged with those specialized expressed in the VGs (Fig. 3a). The fate awaiting these non-functional duplicates appears to be silencing^59^. Furthermore, the Lh genome encodes a total of 66 VG-specialized copies, out of which only 1 copy, *Lar*, was shown functional evidence of lysing the host lymph glands (the highest 3 copies were tested, see Supplementary Fig. 13). The fitness tradeoff between the virulent VPs and energetic cost to produce massive transcripts might partially explain why *Lar* and its associated aggressive strategy were not preserved by other related species. Correspondingly, a previous study at the cellular level also proposed that the immune suppressive strategy is less adaptive than the immune evasive infection strategy^12^.

In conclusion, we have combined comparative genomics, parasitic efficiency assays, functional experiments, and evolutionary analyses to address the rapid speciation and adaptation of two *Leptopilina* species. The most prominent finding of our study is the demystification of the molecular bases and evolutionary scenario of two diverse parasite strategies adopted by the generalist Lh and the specialist Lb (Fig. 5c). To our knowledge, this is the first time that specific genes were characterized associated with divergent parasitic strategies. We also uncovered the evolutionary scenarios of these genes, which add to our understanding of both the rapid speciation across parasitoids and the parasitoid–host coevolution.

## Methods

### Animals

*L. boulardi* (strain G486)^13^ and *L. heterotoma* (strain Lh14)^12^ were reared on *D. melanogaster* (*W*^*1118*^ strain) as regular hosts at 25 °C with a relative humidity of 50% under a 16:8 light:dark cycle. The newly emerged Lb and Lh wasps were provided with apple juice agar medium until exposure to hosts. All Drosophila species, including *D. melanogaster*, *D. simulans*, *D. yakuba*, *D. pseudoobscura*, *D. virilis*, *D. erecta*, *D. santomea*, and *D. suzukii*, were maintained on standard cornmeal/molasses/agar medium at 25 °C in 6-ounce square bottom plastic fly bottles.

### Genome sequencing and de novo assembly

Each line used for sequencing was created from an individual mother and kept in the laboratory for >3 years without outcrossing with other lines. Initial survey of the Lh and Lb genomes was performed using ~24- and 9-Gb Illumina paired-end reads, respectively. 17-mer was counted using Jellyfish v2.2.3^60^. Basic features such as heterozygosity and duplication rate were estimated by GenomeScope v1.0.0 (github.com/schatzlab/genomescope). Both the Lh and Lb genomes showed relatively low levels of heterozygosity (0.364 and 0.475%, respectively; Supplementary Fig. 26).

The genome of Lh was sequenced using long-read sequencing technology (PacBio). Given the tiny body size of *Leptopilina* wasps and the need for a high amount of DNA by the technology, the genomic DNA samples were prepared from ~2000 male adults using a DNeasy Blood and Tissue Kit (Qiagen). We note that there are no sex chromosomes in *Leptopilina*. A 20-Kb genomic library was constructed and sequenced on the PacBio Sequel platform that yielded a total of 31-Gb high-quality long reads. This DNA sample was also sequenced using the Illumina platform to yield 24 Gb (~64× coverage) short reads for the purpose of error correction. Library preparation and sequencing were conducted by Berry Genomics Co. Ltd according to the standard protocols. Information on all Lh sequencing data is presented in Supplementary Table 1. The PacBio long reads were initially corrected to remove potential errors using Canu v1.8^61^. According to the optimal approach previously described^62^, a subset of the ~30× longest corrected reads was used to assemble the genome. SMARTdenovo v2018-02-19 (github.com/ruanjue/smartdenovo) was used to generate the initial contigs with the default parameters. Contigs were further processed with error correction and polished using pilon v1.2.3^63^ with the default parameters.

The genome of Lb was de novo assembled based on a classic shotgun approach that used deep coverage and Illumina sequencing reads of long-insert libraries. DNA was extracted from ~2000 male adults using a standard phenol and chloroform method. To assemble initial contigs, a paired-end library of 450-bp inserts was constructed and sequenced on the long-read mode of the Illumina platform (HiSeq 2500), i.e., each end was sequenced up to 250 bp to enable the bridging of overlapping pair. Libraries of stepwise-increased inserts, including 180-bp, 300-bp, 2-Kb, 3-Kb, 5-Kb, 8-Kb, and 13-Kb inserts, were further generated to join the contigs into scaffolds. These libraries were sequenced in paired-end or mate-pair mode on an Illumina HiSeq X Ten. All the libraries were constructed and sequenced according to the standard Illumina protocols by Berry Genomics Co. Ltd. Information on all Lb sequencing data is presented in Supplementary Table 3. All reads were trimmed to remove low-quality bases by platanus_trim (for paired-end reads) or platanus_internal_trim (for mate-pair reads). DiscovarDeNovo (v52488; software.broadinstitute.org/software/discovar/) was used to assemble 250-bp long reads of 450-bp inserts to generate initial contigs following the default parameters. Redundans^64^ was used to preprocess contigs using paired-end reads from two short-insert libraries (180 and 300 bp). Scaffolding was performed by a stepwise process that employed a mate-pair long-insert library (from 2 to 13 Kb) each time. For each step, ScaffMatch v0.9^65^ was used to join the scaffolds, and GapCloser v1.12^66^ was used to fill the gaps based on paired-end sequence information from short-insert libraries (180 and 300 bp). Rabbit v2.6.18 (github.com/gigascience/rabbit-genome-assembler) was finally used to remove any redundancy that was introduced by the scaffolding processes.

We assessed the completeness of the de novo assemblies using both CEGMA v2.4^67^ and BUSCO v3^68^. The statistics of both assemblies are presented in Supplementary Table 2. We note that there were no released genomes available for *Leptopilina* when this study started in 2016, and no annotated genes are available to date. We also note that both the Lh and Lb genome assemblies generated by this study are of higher quality than any other recently deposited genome assemblies of *Leptopilina* (Supplementary Data 10).

### Transcriptome sequencing

Wasps from critical stages of development were sampled to ensure a good representation of transcripts (Supplementary Fig. 25). Briefly, eight stages of Lh were collected, including Egg, L1 (days 1–3 larvae; early larval stage), L2 (days 4–9 larvae; late larval stage), P1 (days 1–3 pupae; early pupal stage), P2 (days 4–7 pupae; middle pupal stage), P3 (days 8–10 pupae; late pupal stage), AFs, and AM (male adults), and nine stages of Lb were collected, including Egg, L1 (days 1–3 larvae; early larval stage), L2 (days 4–6 larvae; middle larval stage), L3 (days 7–9 larvae; late larval stage), P1 (days 1–3 pupae; early pupal stage), P2 (days 4–7 pupae; middle pupal stage), P3 (days 8–10 pupae; late pupal stage), AFs, and AM (Supplementary Table 6). The VGs of 3-day-old AF wasps were also dissected in Ringer’s saline solution on an ice plate under a stereoscope (Nikon). Total RNA was independently extracted from each sample using the RNeasy Mini Kit (QIAGEN) and subsequently stored at −80 °C for further use. Construction of the cDNA library and paired-end RNAseq (Illumina) were carried out by Berry Genomics Co. Ltd. Statistics of transcriptome sequencing data are listed in Supplementary Table 6. We also sequenced full-length transcripts using the PacBio sequencing system (Pacific Biosciences). A total of 22.0 Gb transcriptome sequencing data of Lb were generated from three independent libraries, with inserts of 1–2, 2–3, and 3–6 Kb, for the messenger RNA (mRNA) pool of all stages of Lb development (Supplementary Table 6). A total of 8.6 Gb transcriptome sequencing data of Lh were generated from one SMRTbell library using the PacBio Sequel sequencing system, with an insert of 1–10 Kb, for the mRNA pool of all stages of Lh development (Supplementary Table 6).

### Genome annotation

Repeat contents were annotated using RepeatMasker v4.0.5 (www.repeatmasker.org) against both the public arthropod set of Repbase v20140131^69^ and a custom library for each species. The species-specific libraries were generated by RepeatModeler v1.0.7 (www.repeatmasker.org) for Lh and Lb (Supplementary Table 4).

The repeat-masked genome assemblies were used to annotate protein-coding genes. Gene prediction was mainly based on the Maker pipeline^70^, which integrated evidence from the transcriptome, homologs of related species, and signatures of gene architectures. The transcriptome evidence consisted of the full-length transcripts and high coverage RNAseq data across all developmental stages (as described above). The full-length transcripts were first mapped to the reference genome using Minimap2 v2.1^71^ with the parameters “-ax splice -uf–secondary=no -C5” and then processed by the submodule “collapse_isoforms_by_sam” of cDNA_Cupcake v6.4 (https://github.com/Magdoll/cDNA_Cupcake). The paired-end Illumina RNAseq reads were aligned to the reference genome using HISAT2 v2.1.0^72^ with the parameter “–dta”, sorted by coordinates using SAMTOOLS v1.9^73^, and assembled into spliced transcripts using StringTie v2.0^74^ with the default parameters. Each sample was assembled independently and merged into a consensus GTF file using the merge mode of StringTie. Homolog evidence was obtained from UniProt and gene sets of the model insect *D. melanogaster* (http://flybase.org/) and five hymenopteran species (*Apis mellifera*, *Fopius arisanus*, *N. vitripennis*, *Polistes dominula*, and *Trichogramma pretiosum*)^10,75–78^. Two predictors, AUGUSTUS v3.3.2^79^ and SNAP v2006-07-28^80^, were used to provide ab initio signatures for Maker; both were trained separately for Lh and Lb using the corresponding full-length transcripts. Maker v2.31.10 was used to integrate the above evidence and generate an automated set of protein-coding genes for Lh and Lb. Predicted genes supported by either expression evidence or homology evidence were retained. We noted that the wasp genome likely encodes a considerable proportion of novel genes, particularly in the VGs, and that such genes were likely to be missed by a single method due to a lack of sufficient evidence. To recover missed novel genes and control the false-positive rate, the final gene set was supplemented with gene loci that were (1) predicted by AUGUSTUS, (2) not overlapping with Maker models, (3) supported by full-length transcripts, and (4) supported by the assembled transcripts of all developmental stages. Statistics of the two official gene sets are presented in Supplementary Table 5.

Each gene was annotated based on the best hit by BLASTP v2.2.26 (*e* < 10^−5^) against the NCBI RefSeq, UniProt, and FlyBase databases. Functional domains were predicted using NCBI’s conserved domain database^81^. Local InterProScan v5.13^82^ was also run with 15 implemented databases to assign IPR domains, Pfam domains, Gene Ontology terms, and KO terms to each gene. Involved databases include CDD, Coils, Gene3D, Hamap, MobiDBLite, Pfam, PIRSF, PRINTS, ProDom, ProSitePatterns, ProSiteProfiles, SFLD, SMART, SUPERFAMILY, and TIGRFAM. *Lar* homologs were further annotated based on the predicted 3D modeling structure using Phyre2^83^.

### Annotation of specific gene families

Protein sequences from *D. melanogaster*, *A. mellifera*, and *N. vitripennis* were used as seeds to annotate known gene families in Lh and Lb. Most gene families were mainly annotated based on reciprocal best BLASTP results (*E* < 1e−5) and subjected to further manual curation. Some gene families, such as chemosensory receptor genes, which have either undergone rapid evolution or contain multiple large introns, are usually difficult to identify by automated predictions. Thus an iterative “TBLASTN-GeneWise” approach was applied to identify these genes in the genome. Briefly, seed sequences were used to identify potential genomic loci using TBLASTN v2.2.26; GeneWise v2.2.0^84^, with the parameters “gb_cutoff=0.3 gw_minlen=50 gw_maxlen=2000”, was used to predict gene structures based on the loci with significant hits (*E* < 10^−5^, TBLASTN). This process was iteratively performed until no new genes were added.

### Comparative genomics

Comparative genomes were performed across representative hymenopteran species with published genomes. OrthoFinder v2.3.3^85^ was used to identify orthologs and paralogs across these species with the default parameters. A total of 2401 single-copy universal genes were used to infer the phylogeny across species. MUSCLE v3.8.3^86^ was used to perform multiple alignments for each ortholog group. Gblocks v0.91b^87^ was used to retrieve conserved blocks within alignments. Conserved blocks from each orthogroup were concatenated to produce a “supergene” with 1,031,968 aas for each species. These supergenes were used to infer a ML phylogenetic tree using GAMMA and the JTT model by RAxML v8.2.10^88^ for 100 replicates of bootstrapping (parameters were set as -f a -x 12345 -p 12345 -# 100 -m PROTGAMMAJTT -s fasta -n tre -k -T 20). The divergence time of each branch was estimated using R8s v1.81^89^ with the parameters “divtime method=lp algorithm=tn”; calibration points were based on a previous study^17^, including *Harpegnathos saltator–A. mellifera* (162), *N. vitripennis*–*A. mellifera* (236), *Neodiprion lecontei*–*A. mellifera* (281), *P. dominula*–*A. mellifera* (179), and *N. vitripennis*–*F. arisanus* (228).

Given the substantial divergence between *Leptopilina* and other species, *d*N/*d*S analysis was not performed outside *Leptopilina* due to substitution saturation. The ratio of synonymous (*d*S) to nonsynonymous (*d*N) sites was calculated for each pair of Lh–Lb orthologs as follows: based on the multiple alignment of protein sequences by MUSCLE v3.8.3^86^, Pal2Nal v13 was used to retrieve the corresponding CDS data; the CodeML module of the PAML package v4.3^90^ was used to calculate *d*N/*d*S with the F3X4 codon frequency. After filtering of *d*S saturation (*d*S > 2), remaining genes were mapped to KEGG pathway based on the annotated KO term. The overall *d*N/*d*S ratio for each pathway was based on the median value of all the involved genes. Pathways including at least three mapped genes were analyzed. Enrichment analyses were performed using the KEGG pathway enrichment analysis module via the online platform “OMICSHARE” (www.omicshare.com). Human disease-related pathways were not considered.

### Expression profiles

Expression profiles across developmental stages were determined as normalized transcripts per million (TPM) for each gene using Salmon v0.12.0^91^ with the parameter “–validateMappings”. Genes with TPM values higher than the N99 value of the corresponding sample were defined as expressed genes, while those with significantly higher TPM values (*Z*-test, *P* < 0.01) were defined as highly expressed genes. Relationships among the developmental stages were calculated based on the overall pattern using the R module “prcomp.” The expression heatmap was based on hierarchical clustering of both samples and genes (the complete method) using the R module “pheatmap.” Enrichment analysis was based on the hypergeometric test using the “phyper” module of R v4.0.0 and corrected with FDR for multiple tests.

### Proteome of the VGs based on liquid chromatography–tandem mass spectrometry (LC-MS/MS)

Approximately 300 VRs of 3-day-old Lh female wasps were dissected in Ringer’s saline solution on an ice plate, washed at least three times in Ringer’s buffer, and then pierced with fine forceps in a cell culture dish to collect the crude venom. After centrifugation at 13,000 × *g* at 4 °C for 1 min, the supernatant (pure venom) was collected and dissolved in 100 μl SDT lysis buffer (4% sodium dodecyl sulfate (SDS), 100 mM Tris-HCl, 1 mM dithiothreitol (DTT), pH 7.6). The sample was boiled for 15 min and then centrifuged at 3,000 × *g* at 4 °C for 40 min. The detergent and DTT of the supernatant were removed with repeated ultrafiltration (Microcon units) using UA buffer (8 M urea, 150 mM Tris-HCl, pH 8.0), followed by adding 100 µl iodoacetamide (100 mM) to block reduced cysteine residues, and the samples were incubated for 30 min in the dark. Then the protein suspensions were digested with 3 µg trypsin (Promega) in 40 µl 100 mM NH_4_HCO_3_ buffer overnight at 37 °C. The resulting peptide mixture was desalted on C18 cartridges (Sigma), concentrated by vacuum centrifugation, and reconstituted in 40 µl of 0.1% (v/v) formic acid for further use.

LC-MS/MS analysis was performed on a Q Exactive mass spectrometer (Thermo Fisher Scientific) coupled to an Easy nLC (Thermo Fisher Scientific). Briefly, a 6-µl aliquot of the peptide mixture was loaded onto a reverse-phase trap column (Thermo Fisher Scientific Acclaim PepMap 100, 100 µm × 2 cm, nanoViper C18) connected to the C18 reversed-phase analytical column (Thermo Fisher Scientific Easy Column, 10 cm long, 75-µm inner diameter, 3 µm resin) in buffer A (0.1% formic acid) and separated with a linear gradient of buffer B (84% acetonitrile and 0.1% formic acid) at a flow rate of 300 nl/min. The eluted peptides were ionized, and the full MS spectrum (from *m*/*z* 300 to 1800) was acquired by precursor ion scan using the Orbitrap analyzer with a resolution of *r* = 70,000 at *m*/*z* 200, followed by 20 MS/MS events in Orbitrap analysis with a resolution of *r* = 17,500 at *m*/*z* 200. The raw data of Lb VPs are downloaded from PeptideAtlas (PASS01481), which was generated in a previous study using the same approach.

### Identification of VPs

VPs were determined by combining the transcriptome and proteome data, i.e., a VG-expressed gene (see above) that completely matched more than three proteomic peptides was defined as a venom protein (Supplementary Data 4).

### Analysis of genomic origin of Lar

Genomic synteny around the locus was analyzed using the promer method implemented in MUMmer v3.23^92^. The TBLASTN-GeneWise iterative approach as described above was used to search for all putative homologs of *Lar* in the genome. This process was first performed in the genomes of Lh and Lb and identified 94 and 6 loci, respectively (Supplementary Data 5). MultAlin^93^ was used to perform multiple alignments across all the identified protein sequences in Lh and Lb. The core region of 170 aathat were relatively conserved across most sequences (Supplementary Fig. 20) was retrieved as a seed to further search for putative homologs in other Hexapoda genomes. To investigate as completely as possible, we independently performed the TBLASTN-GeneWise iterative approach in the 236 Hexapoda genomes that were available by the end of 2018 (Supplementary Data 11). For species outside Hexapoda, we directly used sequences that were deposited in NCBI NR. All identified homologs of *Lar* (340) were used to infer the gene tree. The protein sequences were first multiply aligned by MUSCLE v3.8.3 and manually trimmed to yield the conserved blocks. During this step, sequences without G motifs were further discarded. The retained 211 aa of each sequence were used to infer the ML phylogenetic tree using RAxML^88^ as described above. The tree was rooted using bacterial sequences.

To infer the divergence across Lh and Lb homologs, all involved homolog pairs were aligned using MUSCLE v3.8.3. The pairwise distance (*K*) was calculated with the Kimura two-parameter model^94^ using the distmat program implemented in the EMBOSS package v6.6.0.0 (emboss.sourceforge.net). As control, we identified 2492 orthologous genes between Lh and Lb that were additionally located in macro-synteny regions. The macro-syteny region was called based on the gene order of orthologous genes.

### Analysis of genomic origin of Warm

An alignment revealed no consistent architecture between *Warm* and its homologs in Lb, except for the putative mucin-binding domain (mucin-bd). Based on the documented information in InterPro (IPR004954) and Pfam (PF03272), this domain shows substantial sequence divergence among species and might occur multiple times in a single gene. Thus we traced the origin of *Warm* based only on the documented information of mucin-bd in InterPro and Pfam. The information available as of Feb. 29, 2020 was used in this study (Supplementary Data 7 and 8).

### Sequencing of Lb microbiota

Lb larvae and adults were washed in 75% ethanol for 2 min and were washed with sterilized 1× phosphate-buffered saline (PBS) for three times before sampling. The guts of Lb larvae were carefully dissected in 1× PBS solution on the ice plate under a stereoscope (Nikon). In total, 30 Lb adults and 150 Lb midguts for each group (two replicates in this study) were collected for metagenomic sequencing. The DNA was extracted by using the Qiagen DNAEasy Extraction Kit. Library preparation and sequencing on an Illumina HiSeq X Ten platform were conducted by Tiny Gene Bio-Tech Co. Ltd according to the standard protocols. The average size of metagenomic data for each sample was approximately 6.0 G. Sequences were then introduced into SeqPrep (https://github.com/jstjohn/SeqPrep) to remove the adapters and were trimmed by Sickle (https://github.com/najoshi/sickle) with the default parameters to obtain clean reads. The clean reads were compared with Lb genome to remove host DNA sequences by using BWA^95^. The remaining clean reads were then assembled by Megahit (https://github.com/voutcn/megahit). CD-HIT^96^ was used to cluster the assembled reads (parameters: identity = 95%, coverage = 90%) to construct a non-redundant gene set, in which the longest reads served as the representative sequences of the specific microbial species. Salmon^90^ was used to do the alignments (95% identity) with the assembled reads to the non-redundant gene set to obtain the abundance of each microbial species in each sample. The taxonomies annotation was performed against NR database with e-value of 1e−5 by the BLASTP.

### Parasitic efficiency assay and wasp egg attachment rate

Three-day-old female wasps were allowed to parasitize second instar *Drosophila* larvae at parasite/host ratios of 1:10 (Lb) and 1:20 (Lh) for 1 h. Then, 4 h later, some of the parasitized host larvae were dissected under a microscope to detect the status of the wasp eggs. The attachment rate was calculated as the number of attached wasp eggs divided by the total number of eggs multiplied by 100. Next, the remaining parasitized hosts were maintained at 25 °C for analysis of parasitism efficiency.

Similar approaches were conducted to calculate the parasitism efficiency of Lb and Lh when they were introduced to different *Drosophila* species. The parasitism rate and wasp emergence rate were calculated using the following formulas:

Parasitism rate = (1 − number of emerged *Drosophila* adults/number of total hosts) × 100%;

Wasp emergence rate = (number of emerged wasps/number of total hosts) × 100%.

### TUNEL (terminal deoxynucleotidyl transferase-mediated dUTP-fluorescein nick end labeling) assay

Apoptosis detection was carried out with Click-iT Plus TUNEL Alexa Fluor 594 Imaging Assay (Invitrogen) according to the manufacturer’s protocol. Briefly, host larval lymph glands were dissected 12 h after parasitization and then fixed in 4% paraformaldehyde in 1× PBS for 15 min at room temperature, followed by a permeabilization step with 0.25% Triton™ X-100 for 20 min, and washed twice with deionized water. The samples were then covered with TdT Reaction Buffer for 10 min at 37 °C and incubated with a TUNEL reaction mixture containing EdUTP and TdT enzyme for 1 h at 37 °C. After incubation, the samples were washed twice with 3% bovine serum albumin (BSA) in 1× PBS for 5 min and subsequently incubated with prepared fresh Click-iT™ Plus TUNEL reaction cocktail for 30 min at 37 °C in the dark. Finally, the samples were washed with 3% BSA in 1× PBS for 5 min and mounted in ProLong Gold Antifade Mountant with DAPI (Invitrogen). The images were captured using a Zeiss LSM 800 confocal microscope and processed using Photoshop (Adobe).

### RNAi in vivo

For RNAi, 400–600-bp fragments of the target genes were PCR amplified from the complementary DNA. Then T7 promoter sequence primers were used for PCR amplification of the dsRNA templates. The dsRNA was synthesized using a T7 RiboMAX Express RNAi Kit (Promega) according to the manufacturer’s instructions. A 438-bp coding sequence from green fluorescent protein (GFP) was used as a control dsRNA (dsGFP). The dsRNA was quantified using a Nanodrop 2000 spectrophotometer (Thermo Scientific). All the dsRNA synthesis primers used in this study are listed in Supplementary Data 12. A total of 20 nl of dsRNA (5 μg/μl) was injected into the abdomen of each fifth instar wasp larva using the Eppendorf FemtoJet 4i Microinjector with the following parameters: injection pressure = 900 hPa; injection time = 0.15 s. After the dsRNA-treated parasitoids eclosed, they were used to parasitize the hosts. Three biological replicates were performed. The RNAi efficiencies are listed in Supplementary Figs. 12 and 24.

### Quantitative real-time PCR

Total RNA was extracted using the RNeasy Mini Kit (Qiagen) and then reverse transcribed into cDNA using HiScript III RT SuperMix for qPCR (Vazyme) according to the manufacturer’s protocol. qRT-PCR was performed in the AriaMx real-time PCR system (Agilent Technologies) with the ChamQ SYBR qPCR Master Mix Kit (Vazyme). Reactions were carried out for 30 s at 95 °C, followed by 45 cycles of three-step PCR for 10 s at 95 °C, 20 s at 55 °C, and 20 s at 72 °C. The RNA levels of the target genes were normalized to that of tubulin mRNA, and the relative concentration was determined using the 2^−ΔΔCt^ method. All the primers used for qRT-PCR in this study are listed in Supplementary Data 12.

### Immunohistochemistry

VGs of Lh and lymph glands of *D. melanogaster* host larvae were dissected separately in 1× PBS and fixed in 4% paraformaldehyde in PBS for 30 min, rinsed three times with 1× PBST (PBS containing 0.1% Triton X-100 and 0.05% Tween 20), blocked with 1% bovine serum albumin in 1× PBST, and stained overnight at 4 °C with anti-Lar primary antibody (1:1000). After three 1× PBST washes, the VGs or lymph glands were incubated with Alexa Fluor 488 or 594 secondary antibodies (1:1000; Molecular Probes) for 2 h at room temperature, respectively. Samples were mounted in ProLong Gold Antifade Mountant with DAPI (Invitrogen). Fluorescence images were captured on a Zeiss LSM 800 confocal microscope and processed using Photoshop (Adobe).

### Western blot analysis

An anti-Lar antibody was generated in rabbits against a peptide (CLEDINYLLSKKEAKEE) derived from the Lh Lar protein. Total proteins from the wasp venom apparatus and carcass were isolated with protein extraction buffer (Sangon Biotech). After centrifugation at 12,000 × *g* at 4 °C for 10 min, the supernatant was quantified with the BCA Protein Assay Kit (Invitrogen), and the same amount of proteins was reserved for SDS–polyacrylamide gel electrophoresis analysis, and the proteins were transferred to polyvinylidene difluoride membranes (Millipore). The membranes were incubated in a blocking solution (Tris-buffered saline containing 0.1% Tween 20, 2% BSA) for 3 h and probed overnight at 4 °C with anti-Lar primary antibody (1:2000 dilution). The horseradish peroxidase-conjugated anti-rabbit IgG secondary antibody (Solarbio) was used at a dilution of 1:2000.

### Data analysis and statistics

Data were analyzed for statistical significance using Student’s *t* tests. All statistical analyses were performed in GraphPad Prism version 8.0 (GraphPad Software). Error bars indicate the standard deviation (SD), and the data are expressed as the mean ± SD. Throughout the paper, significance values are indicated as *P < 0.05, **P < 0.01, ***P < 0.005, and ****P < 0.001.

### Reporting summary

Further information on research design is available in the Nature Research Reporting Summary linked to this article.

## Supplementary information

Supplementary Information

Peer Review File

Reporting Summary

Description of Additional Supplementary Files

Supplementary Data 1–12

## Data Availability

The transcript sequences of *Lar* and *Warm* were deposited in GenBank with the accession numbers MT431620 (*Lar*) and MT439843 (*Warm*). Genomic data and associated transcriptome data are available in NCBI GenBank under BioProject numbers PRJNA624738 (Lh) and PRJNA624743 (Lb). The proteome data of the venom fluids were deposited in PeptideAtlas under the accession number PASS01574. The microbiota sequencing data were deposited in NCBI SRA under the accession number SRP259519. The authors declare that all data supporting the findings of this study are available within the paper and its supplementary information files or from the corresponding author upon request. Source data are provided with this paper.

## Source data

Source Data

## Code availability

All analyses were performed using the public software, which have been listed in the relevant sections in “Methods.”
