# Supplementary material for: Two novel venom proteins underlie divergent parasitic strategies between a generalist and a specialist parasite
Source: Nat Commun. 2021 Jan 11;12:234. doi: 10.1038/s41467-020-20332-8 (PMC7801585; doi:10.1038/s41467-020-20332-8)
Supplement: Supplementary file 1 — Supplementary Information [file 41467_2020_20332_MOESM1_ESM.pdf]

Supplementary information for

Two novel venom proteins underlie divergent parasitic strategies between a generalist and a specialist parasite

Supplementary Figure 1 -------------------------------- 2
Supplementary Figure 2--------------------------------- 3
Supplementary Figure 3--------------------------------- 4
Supplementary Figure 4--------------------------------- 5
Supplementary Figure 5--------------------------------- 6
Supplementary Figure 6--------------------------------- 7
Supplementary Figure 7--------------------------------- 8
Supplementary Figure 8--------------------------------- 9
Supplementary Figure 9-------------------------------- 10
Supplementary Figure 10------------------------------- 11
Supplementary Figure 11------------------------------- 12
Supplementary Figure 12------------------------------- 13
Supplementary Figure 13------------------------------- 14
Supplementary Figure 14------------------------------- 16
Supplementary Figure 15------------------------------- 17
Supplementary Figure 16------------------------------- 18
Supplementary Figure 17------------------------------- 20
Supplementary Figure 18------------------------------- 21
Supplementary Figure 19------------------------------- 22
Supplementary Figure 20------------------------------- 23
Supplementary Figure 21------------------------------- 24
Supplementary Figure 22------------------------------- 25
Supplementary Figure 23------------------------------- 26
Supplementary Figure 24------------------------------- 27
Supplementary Figure 25------------------------------- 28
Supplementary Figure 26------------------------------- 29
Supplementary Table 1--------------------------------- 30
Supplementary Table 2--------------------------------- 31
Supplementary Table 3--------------------------------- 32
Supplementary Table 4--------------------------------- 33
Supplementary Table 5--------------------------------- 34
Supplementary Table 6--------------------------------- 35
Supplementary Table 7--------------------------------- 36
Supplementary References------------------------------ 37

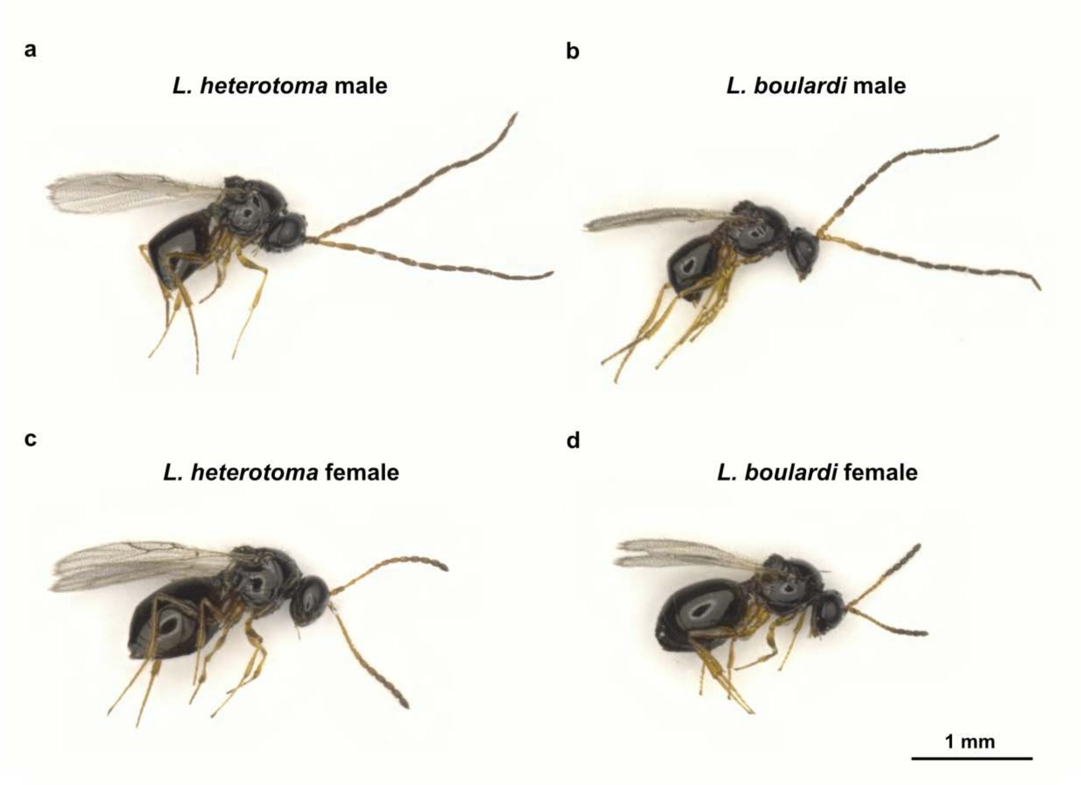

Supplementary Figure 1 ***Leptopilina heterotoma and L. boulardi***

Representative images of *L. heterotoma* (Lh) adult male (a) and female (c), and *L. boulardi* (Lb) adult male (b) and female (d). Scale bar: 1 mm.

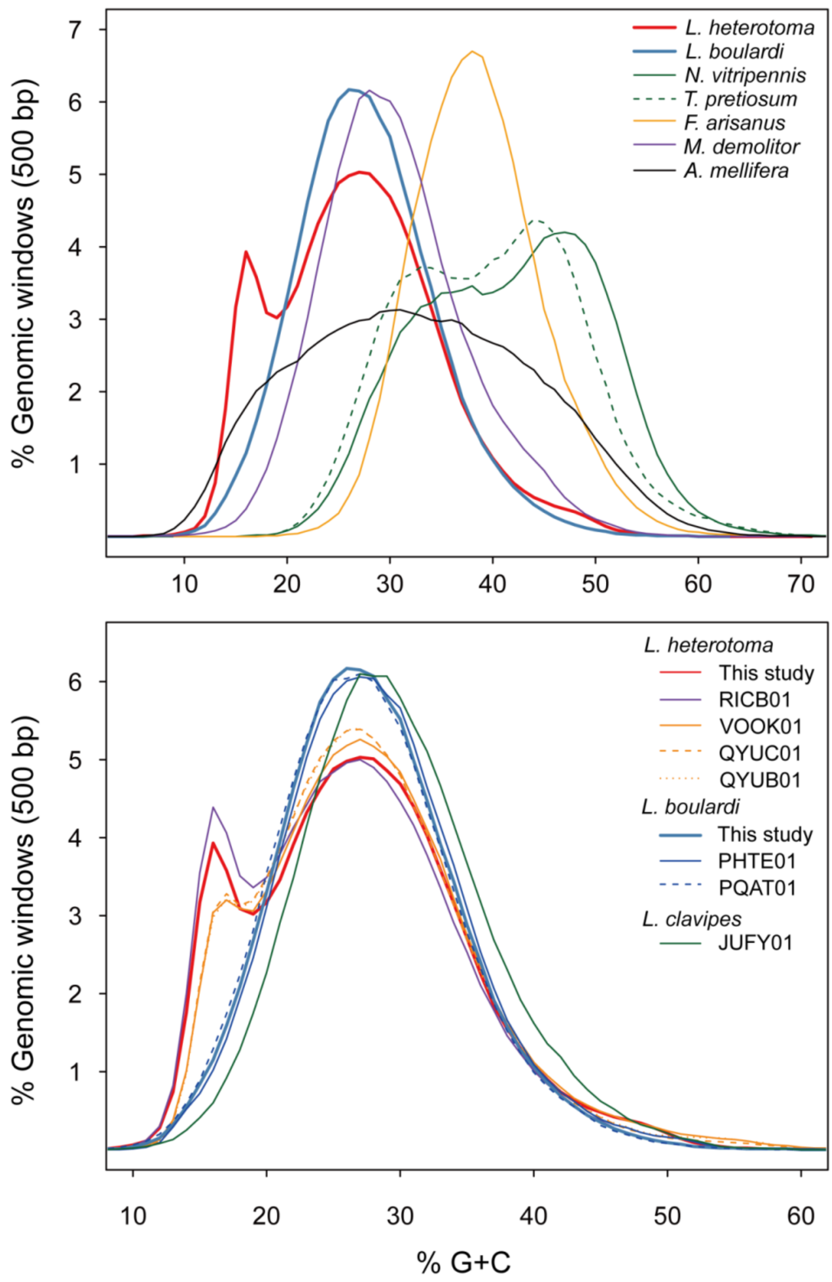

Supplementary Figure 2 Distribution of GC content across representative Parasitoida species.

RICB01, VOOK01, QYUC01, QYUB01, PHTE01, PQAT01, JUFY01 indicate NCBI accession numbers of other available *Leptopilina* genomes.

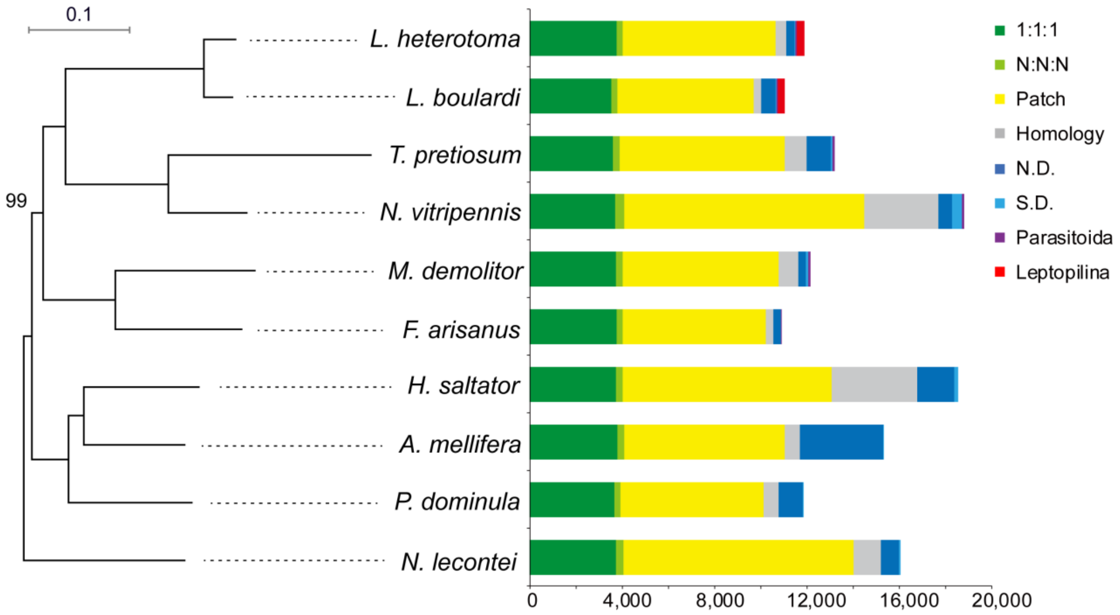

Supplementary Figure 3 Phylogenomics and orthology across representative hymenopteran species.

Orthology was analyzed across 10 representative hymenopteran species as follows. Gene repertoire of each species was divided to a given orthology type as indicated: "1:1:1", universal single-copy gene families across all examined species allowing absence or duplication in one genome; "N:N:N", other universal genes; "Parasitoida", orthologs specific to Parasitoida; "Leptopilina", orthologs specific to *Leptopilina*; "Patchy", all other orthologs across species; "S.D.", species-specific duplication; "Homology", genes with partial homology detected with $E<10^{-5}$ but no orthology assigned; "N.D.", species-specific genes. The maximum-likelihood (ML) phylogenomic tree was calculated based on the concatenated alignments of 2704 exactly single-copy proteins in each species, rooted using *N. lecontei*. Bootstrap values are equal to 100 (out of 100 replicates) unless a number is labeled.

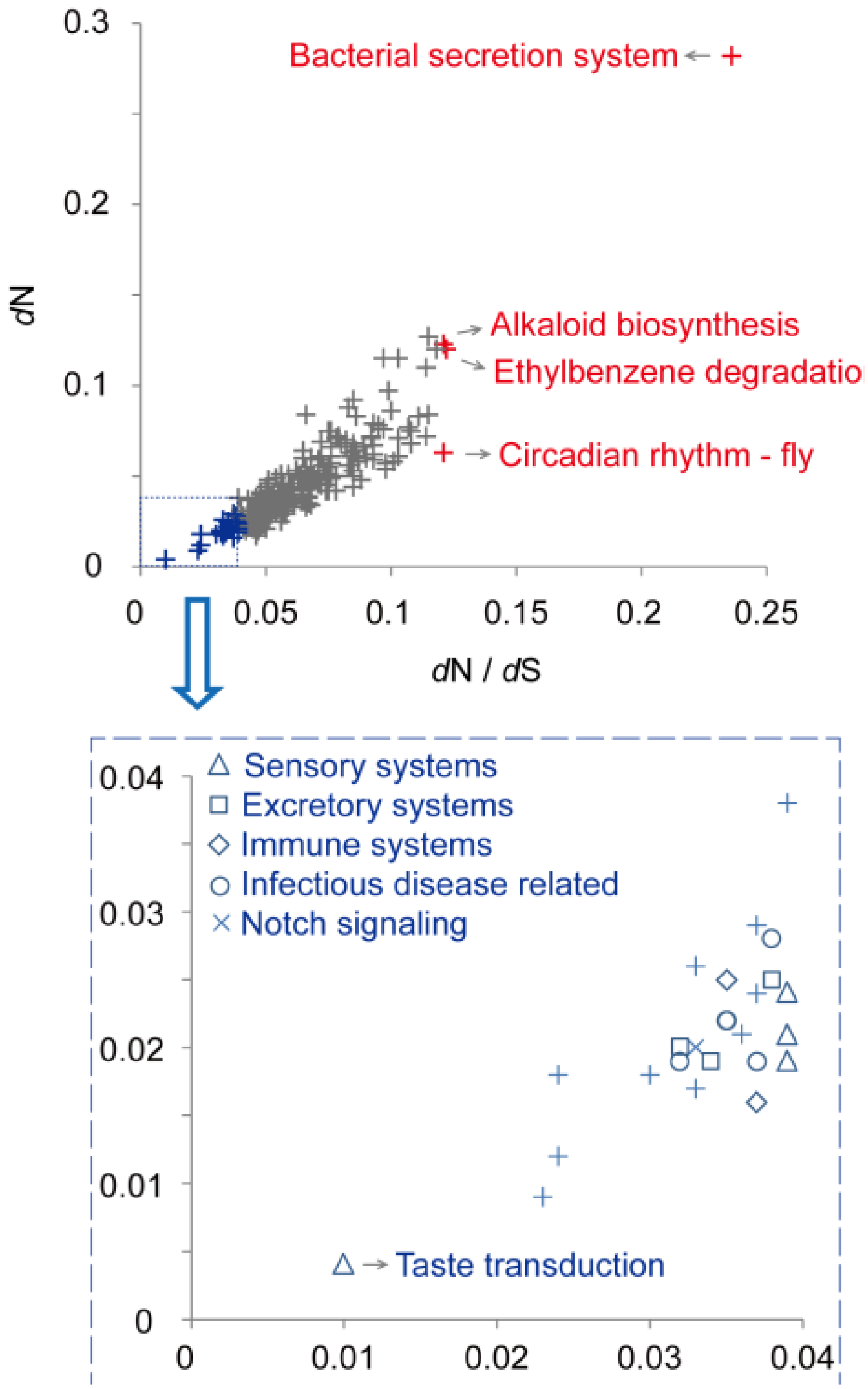

Supplementary Figure 4 Adaptive divergence between *L. heterotoma and L. boulardi* based on the nonsynonymous-to-synonymous substitution ratios (d_N/d_S).

KEGG pathways to which all orthologous genes between *L. heterotoma* and *L. boulardi* were mapped are indicated by median dN/dS ratios and dN. Red dots indicate outlier pathways of potential signature of rapid evolution, while blue dots, as shown enlarged below, indicate pathways that are extremely conserved between Lh and Lb. See detailed information in Supplementary Data 1.

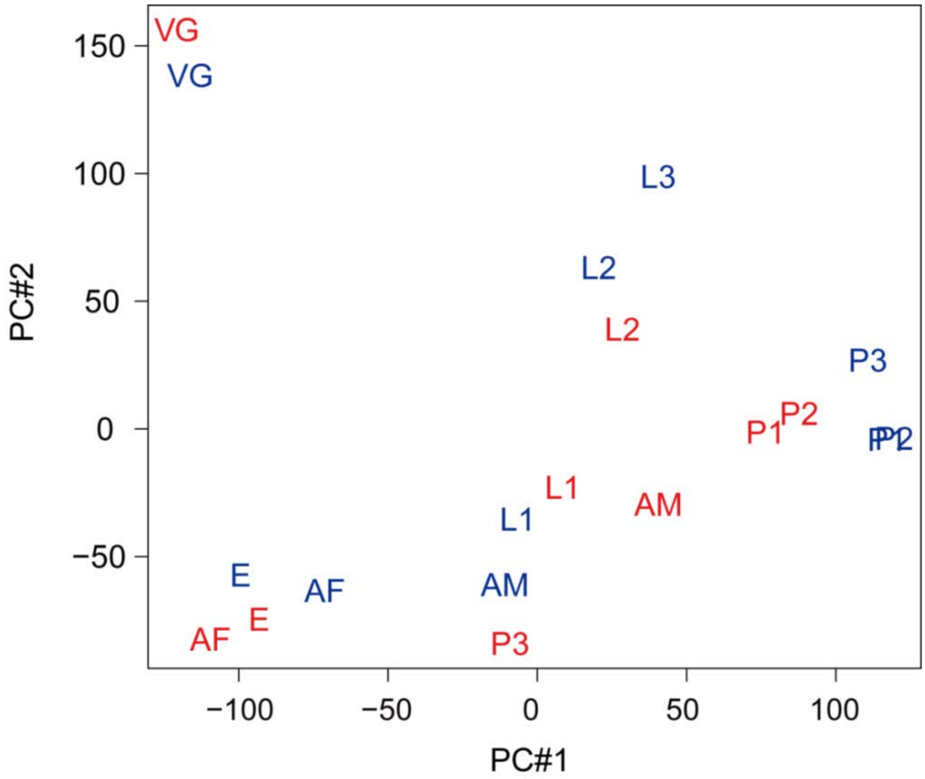

Supplementary Figure 5 Principle component analysis of Lh and Lb samples across different developmental stages and venom glands.

Analysis was based on overall expression of each sample. E, eggs; L1, days 1-3 larvae; L2, days 4-9 larvae for Lh while days 4-6 larvae for Lb; L3, days 7-9 larvae for Lb; P1, days 1-3 pupae; P2, days 4-7 pupae; P3, days 8-10 pupae; AF, female adults; AM, male adults; VG, venom glands. Names in red indicate Lh samples, while those in blue indicate Lb samples.

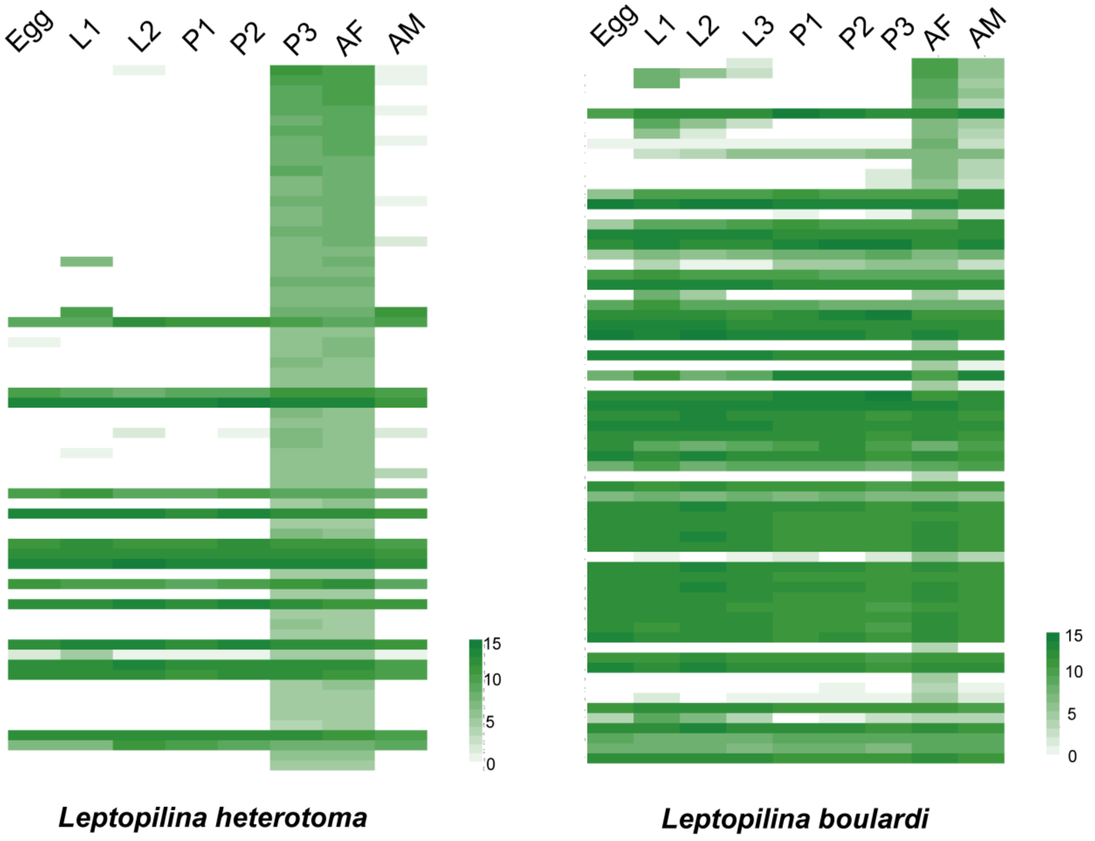

Supplementary Figure 6 Expression heatmap of VG-highly expressed genes across developmental stages.

Expression is presented in log2 scale. Samples correspond to those in Supplementary Fig. 5.

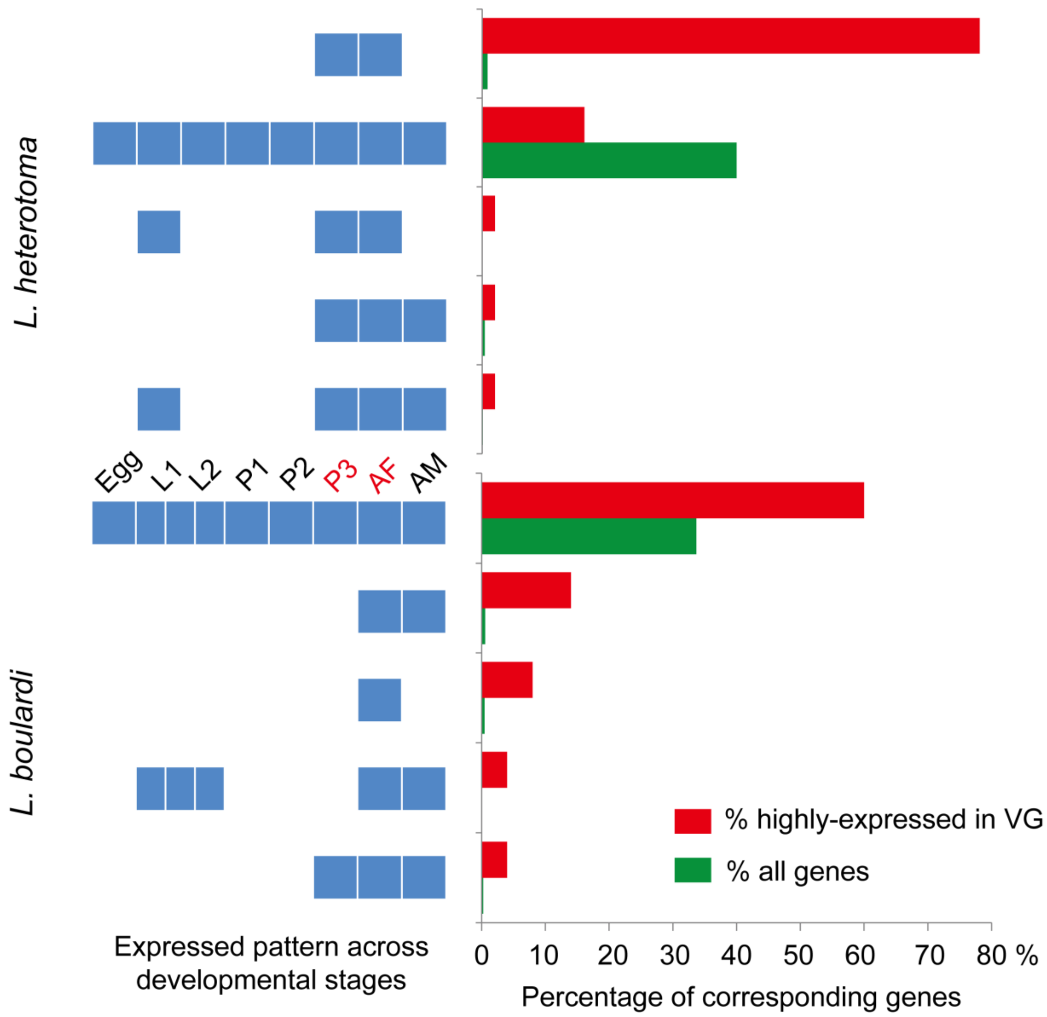

Supplementary Figure 7 Sample distribution comparison between VG-highly expressed genes and all predicted genes.

Blue cells indicate expression in the corresponding developmental stage. Samples correspond to those in Supplementary Fig. 5.

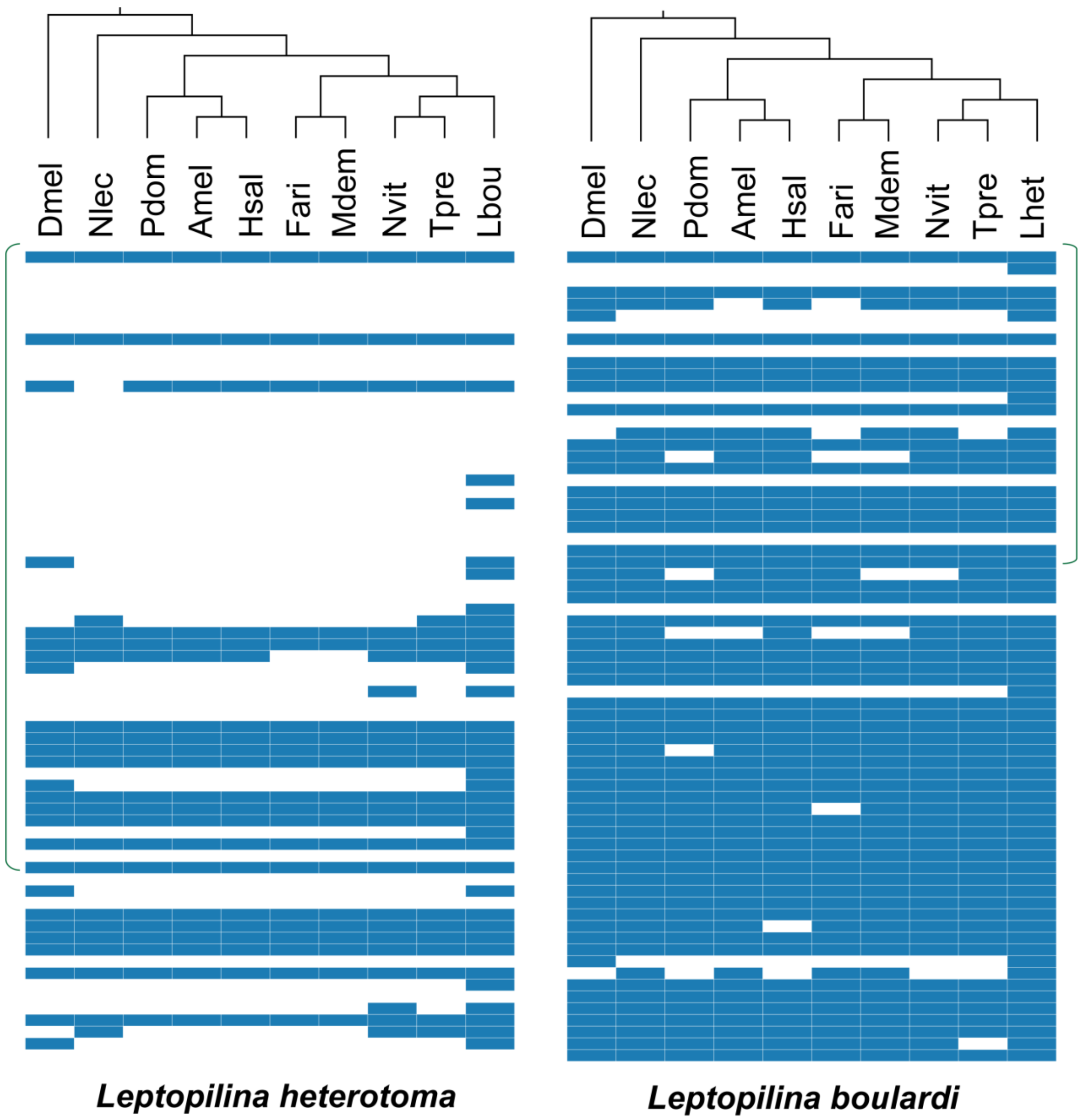

Supplementary Figure 8 Heatmap of presented homolog of VG-highly expressed genes in other hymenopterans.

Blue cells indicate the presence of homology in the corresponding species. Lhet, *L. heterotoma*; Lbou, *L. boulardi*; Tpre, *T. pretiosum*; Nvit, *N. vitripennis*; Mdem, *M. demolitor*; Fari, *F. arisanus*; Hsal, *H. saltator*; Amel, *A. mellifera*; Pdom, *P. dominula*; Nlec, *N. lecontei*; Dmel, *D. melanogaster*.

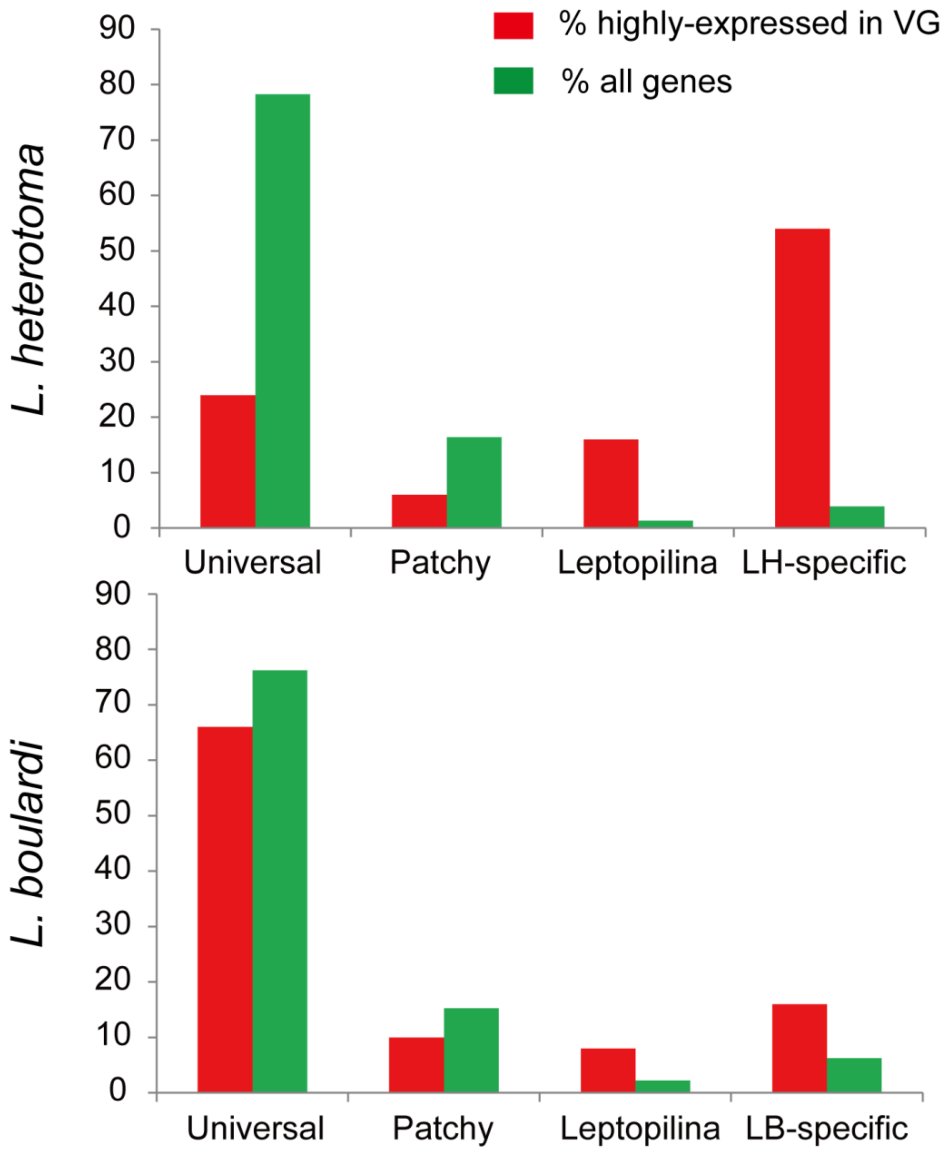

Supplementary Figure 9 Origin distribution comparison between VG-highly expressed genes and all predicted genes.

Universal, genes presenting homology in at least eight out of the other nine hymenopteran species (see Supplementary Fig. 3 for species information); Patchy, genes presenting homology in several hymenopteran species; Leptopilina, genes presenting homologs only in Lh and Lb; -specific, species specific genes.

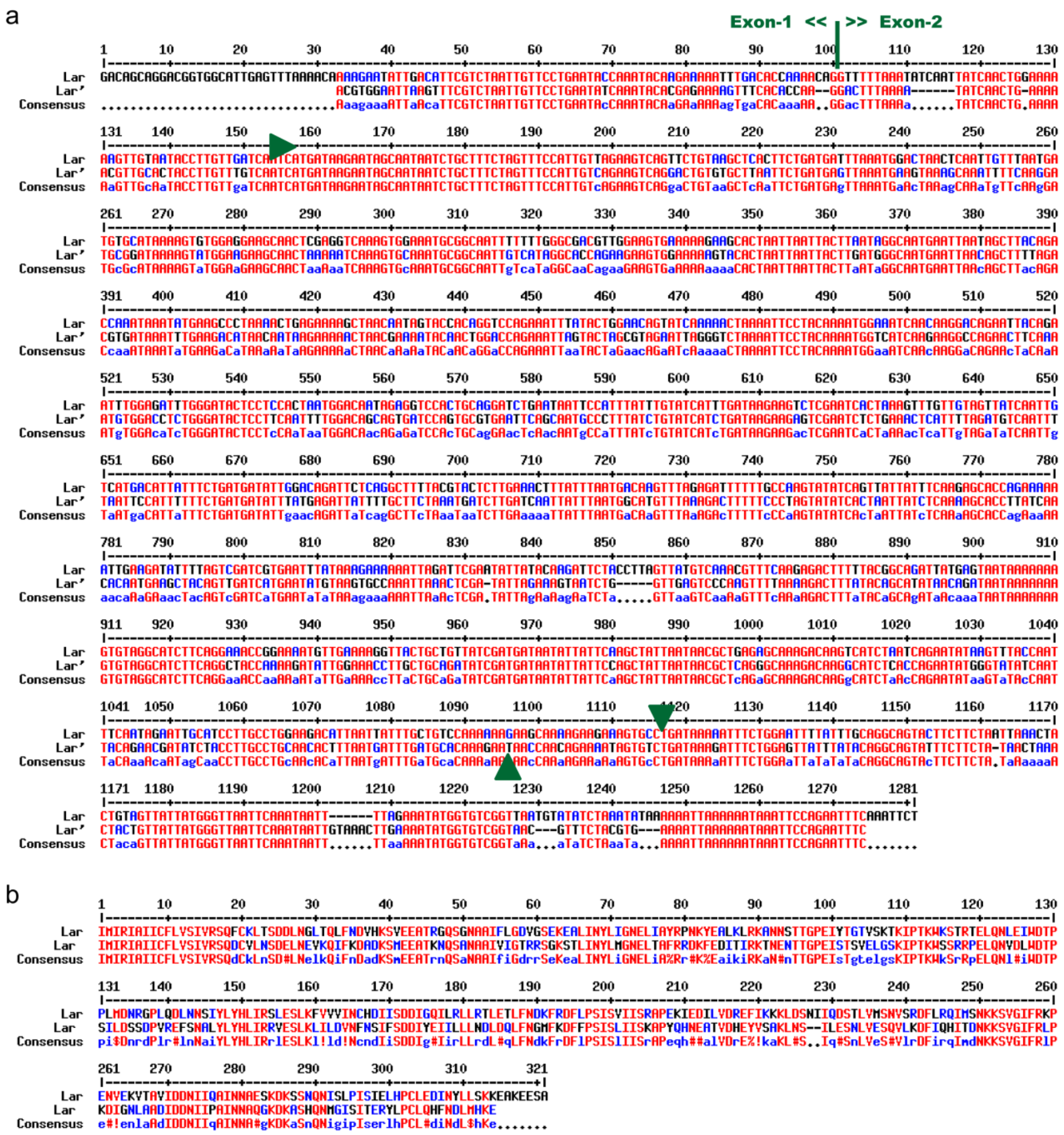

Supplementary Figure 10 Sequence alignment between *Lar* and *Lar'*.

a Alignment at the nucleotide level. Vertical line indicates the splicing boundary between the first and the second exon. The forward triangle indicates the translation start site; the downward triangle indicates the stop site of *Lar*, while the upward triangle inidicates that of *Lar'*. **b** Alignment at the amino acid level. Alignment plots were generated using MultAlin[1]. Note that the overall sequence identity of amino acids is 54%, which is even lower than that of nucleotides (73%), suggesting a signature of rapid evolution.

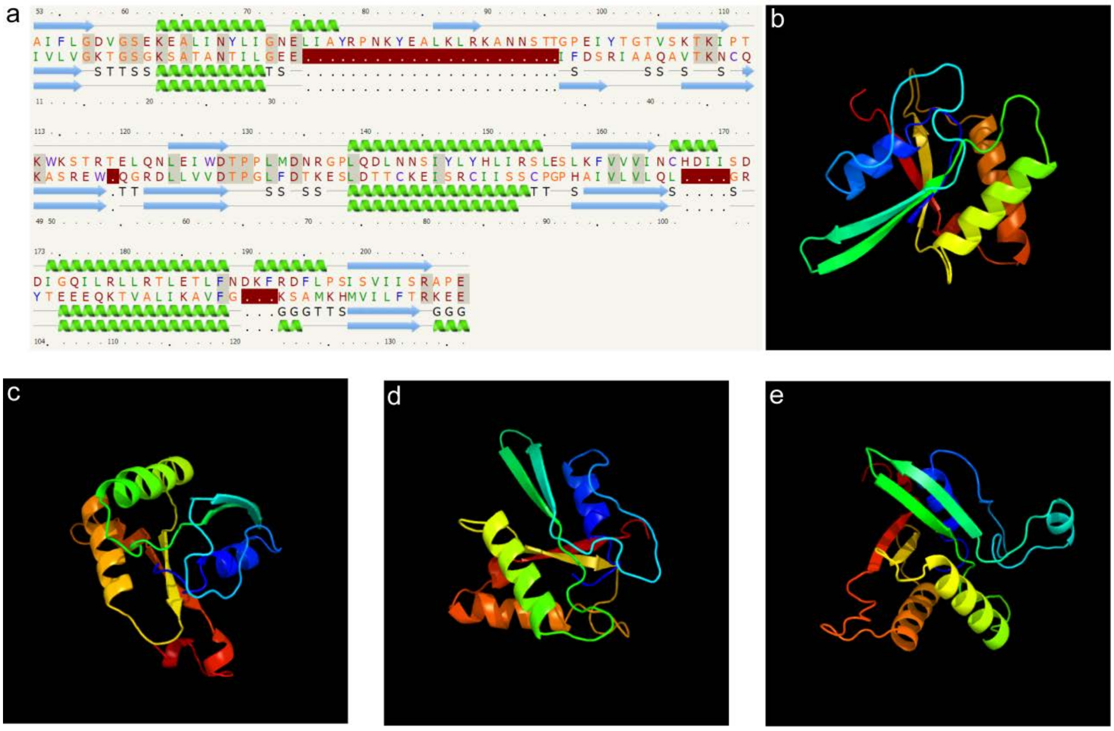

Supplementary Figure 11 Putative modeled structures of *Lar* and its representative homologs.

a Alignment of Lar with the highest scoring template (c3zjcC, 20% identity and 99% confidence). For each set of alignment predicted by Phyre2[2], rows from top to bottom indicate the predicted secondary structure of Lar, amino acid sequence of Lar, amino acid sequence of the template, the known secondary structure of the template, and the predicted secondary structure of the template, respectively. The 3D model structure is shown in **b**, along with modeled structures of its representative homologs: **c**, a copy of Sericostoma (group II); **d**, a copy of *N. vitripennis* (group I); **e**, a fungal copy of *Rhizophagus clarus* (outgroup).

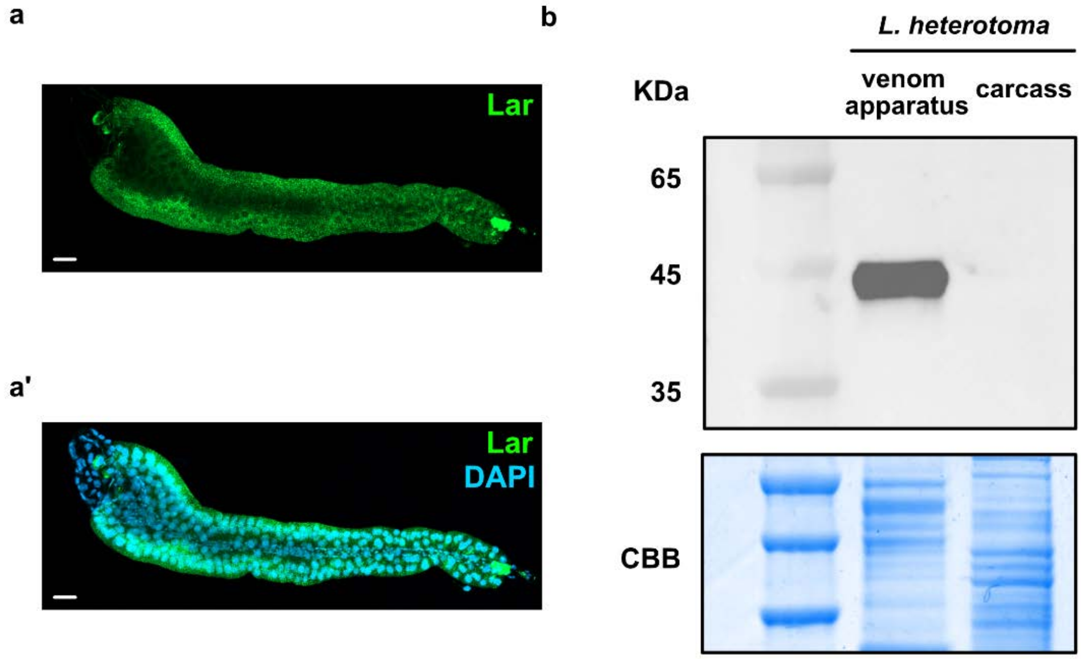

Supplementary Figure 12 Lar is specifically expressed in the VG of *L. heterotoma*.
a Immunolocalization of Lar (green) in Lh venom gland (n=3 replicates, at least 20 lymph glands were examined for each individual). **a’** Merged image of Lar staining (green) and nuclei stained with DAPI (blue). Scale bars: 20 µm. **b** Western blot analysis of Lar in parasitoid venom apparatus and carcass (n=3 replicates). CBB: Coomassie Brilliant Blue. The same amount of proteins was loaded after quantification with BCA Protein Assay kit, and CBB is the indicator of loading samples.

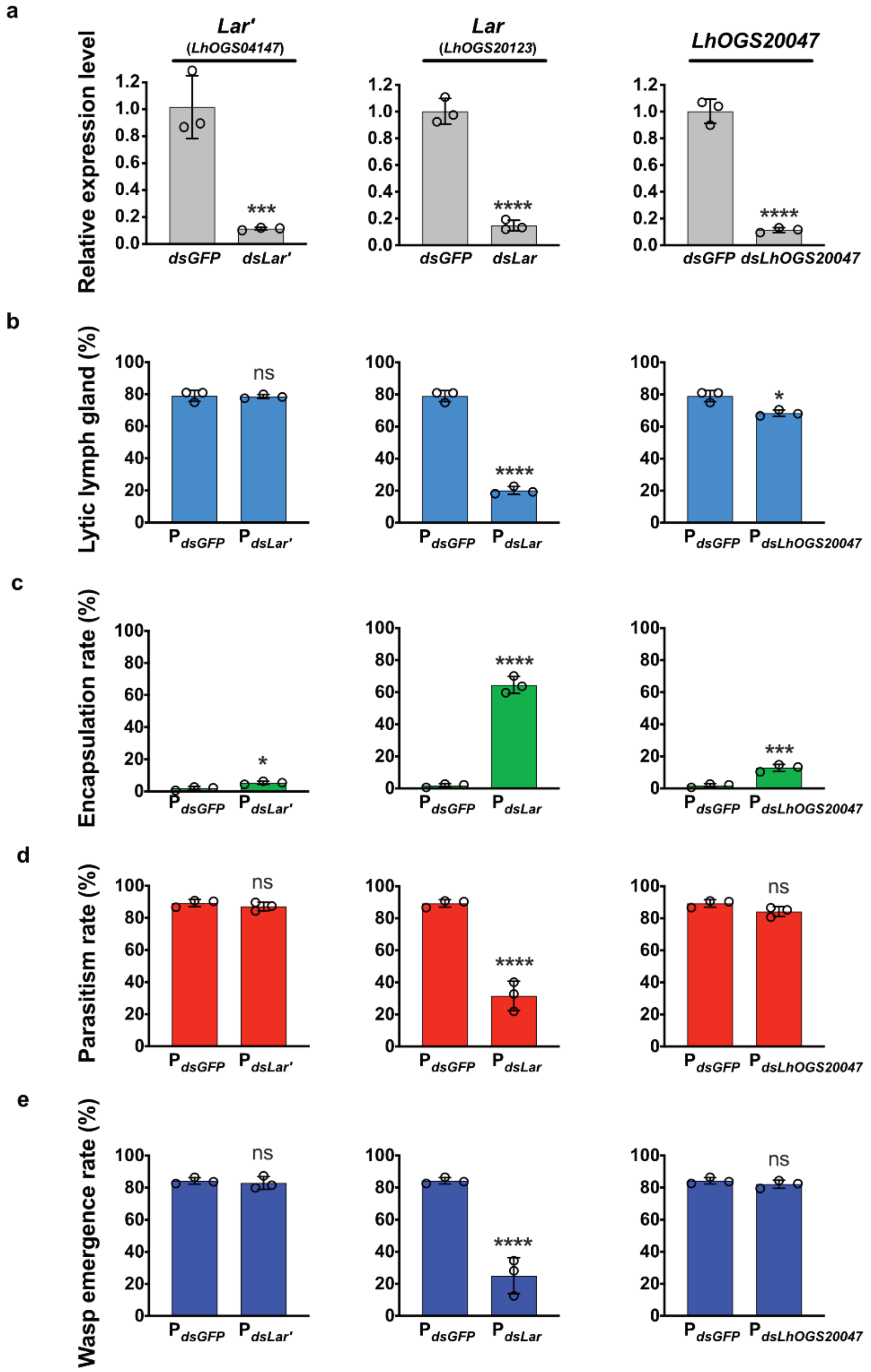

Supplementary Figure 13 Parasitic efficacy assays for different dsRNA-treated Lh.
a Relative mRNA levels of *Lar*, *Lar'* and *LhOGS20047* in Lh after RNAi treatments. Three biological replicates were performed. Data are means±SD; Significance was determined by two-tailed unpaired Student's t-test (*dsLar'*: *P*=0.0026; *dsLar*: *P*=0.0001; *dsLhOGS20047*: *P*=7.7e-5). **b** Percentage of host larvae exhibiting lytic lymph gland 24 h after parasitization

by *dsGFP*-treated Lh (P_{dsGFP}, n=123), *dsLar'*-treated Lh ($P_{dsLar'}$, n=116), *dsLar*-treated Lh (P_{dsLar}, n=120), and *dsLhOGS20047*-treated Lh ($P_{dsLhOGS20047}$, n=127). Three biological replicates were performed. Data are means±SD; Significance was determined by two-tailed unpaired Student's t-test ($P_{dsLar'}$: *P*=0.8527; P_{dsLar}: *P*=1.8e-5; $P_{dsLhOGS20047}$: *P*=0.011). **c** Percentage of host larvae containing encapsulation capsules after parasitization by the above dsRNA-treated Lh wasps (P_{dsGFP}, n=893; $P_{dsLar'}$, n=951; P_{dsLar}, n=693; $P_{dsLhOGS20047}$, n=1082). Three biological replicates were performed. Data are means±SD; Significance was determined by two-tailed unpaired Student's t-test ($P_{dsLar'}$: *P*=0.0134; P_{dsLar}: *P*=4.1e-5; $P_{dsLhOGS20047}$: *P*=0.0016). **d** Parasitism rate in host larvae after parasitization by dsRNA-treated wasps in c. Three biological replicates were performed. Data are means±SD; Significance was determined by two-tailed unpaired Student's t-test ($P_{dsLar'}$: *P*=0.3505; P_{dsLar}: *P*=0005; $P_{dsLhOGS20047}$: *P*=0.0809). **e** Wasp emergence rate in host larvae after parasitization by dsRNA-treated wasps in c. Three biological replicates were performed. Data are means±SD; Significance was determined by two-tailed unpaired Student's t-test ($P_{dsLar'}$: *P*=0.639; P_{dsLar}: *P*=0008; $P_{dsLhOGS20047}$: *P*=0.318). *:$P < 0.05$;***: $P < 0.005$; ****: $P < 0.001$; ns: not significant.

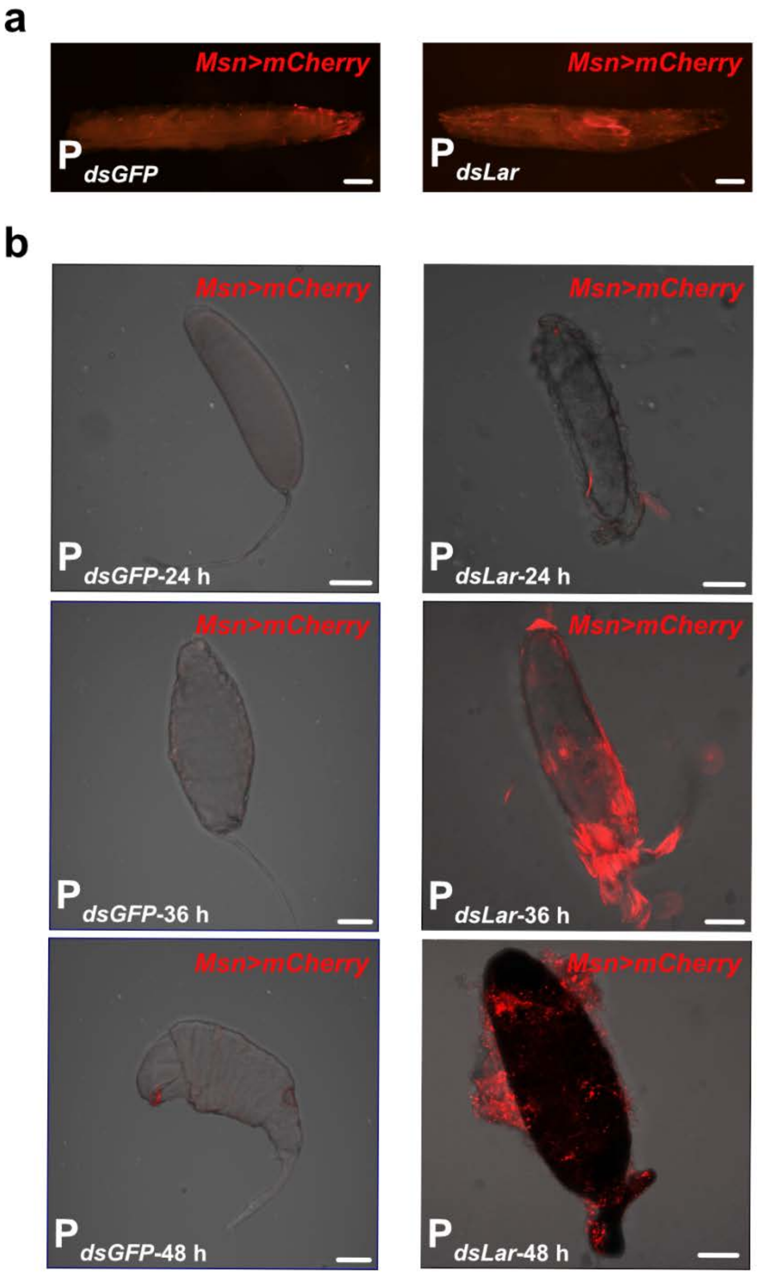

Supplementary Figure 14 Lar contributes to suppress the lamellocyte differentiation and encapsulation immune responses.

a Lamellocytes, labeled by the Msn>mCherry marker (Red) were rarely found in host post-infection by *dsGFP*-treated Lh (P_{dsGFP}). However, lamellocytes were largely induced upon *dsLar*-treated Lh infection (P_{dsLar}) (n=3 replicates, at least 50 *Drosophila* larvae were examined for each individual). Scale bars: 1 mm. **b** There was no encapsulation responses due to the lack of lamellocytes in P_{dsGFP} infected host larvae. Then, the wasp egg successfully developed to larval stage 48 h later. The triggered lamellocytes initiated to adhere to the wasp egg at 24 h, encapsulated the wasp egg at 36 h, and completely encapsulated and melanized the wasp egg at 48 h in host larvae after infection by P_{dsLar} (n=3 replicates, at least 50 wasps were examined for each individual). Scale bars: 20 μm.

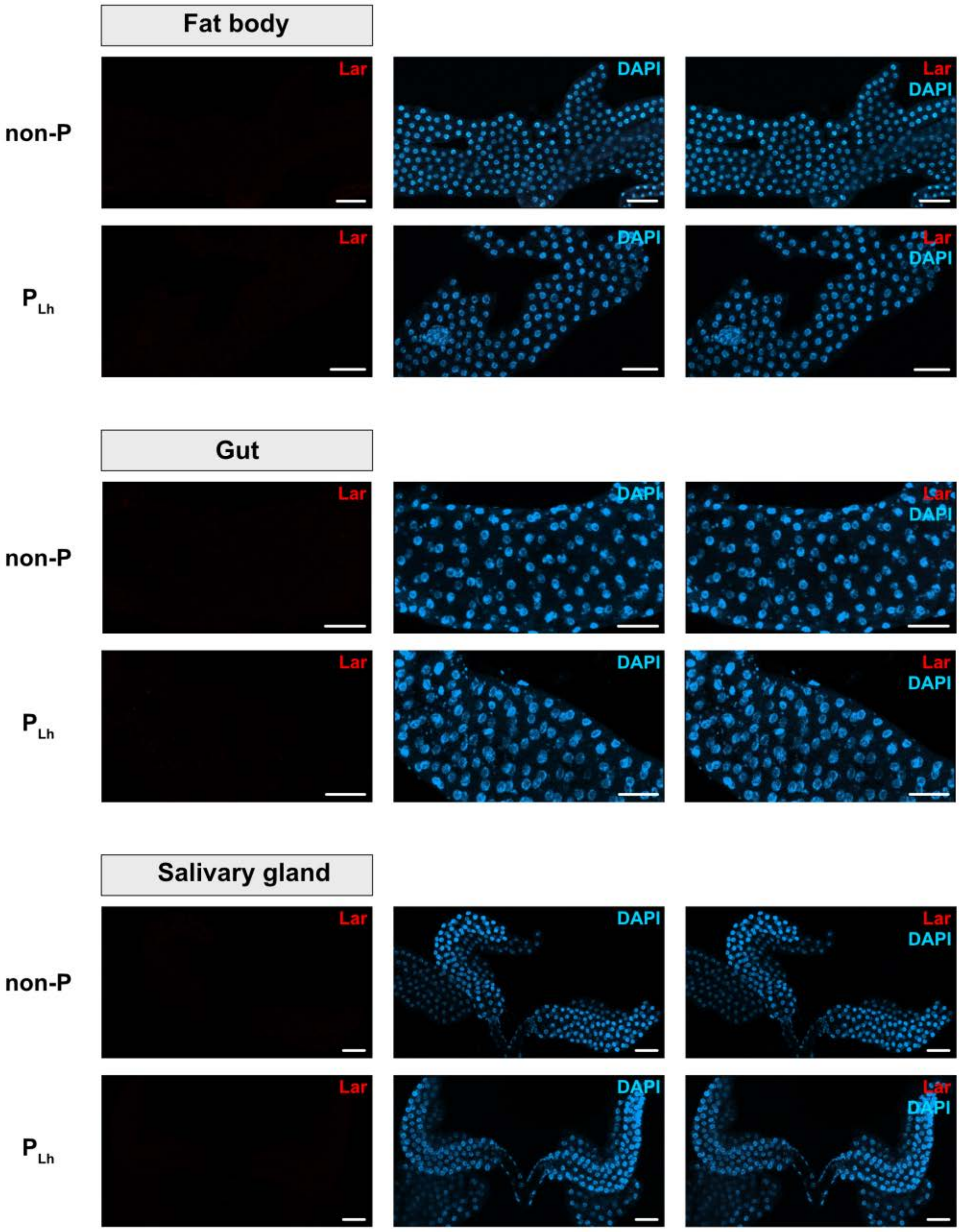

Supplementary Figure 15 The distribution of Lar in different host tissues.
Fluorescent images of fat body, gut and salivary gland from unparasitized host (non-P) and parasitized host (P_{Lh}). Tissues were dissected 24 h after parasitization by *L. heterotoma* females, and stained with anti-Lar (red) and DAPI (blue) (n=3 replicates, at least 20 *Drosophila* tissues were examined for each individual). Scale bars: 50 µm.

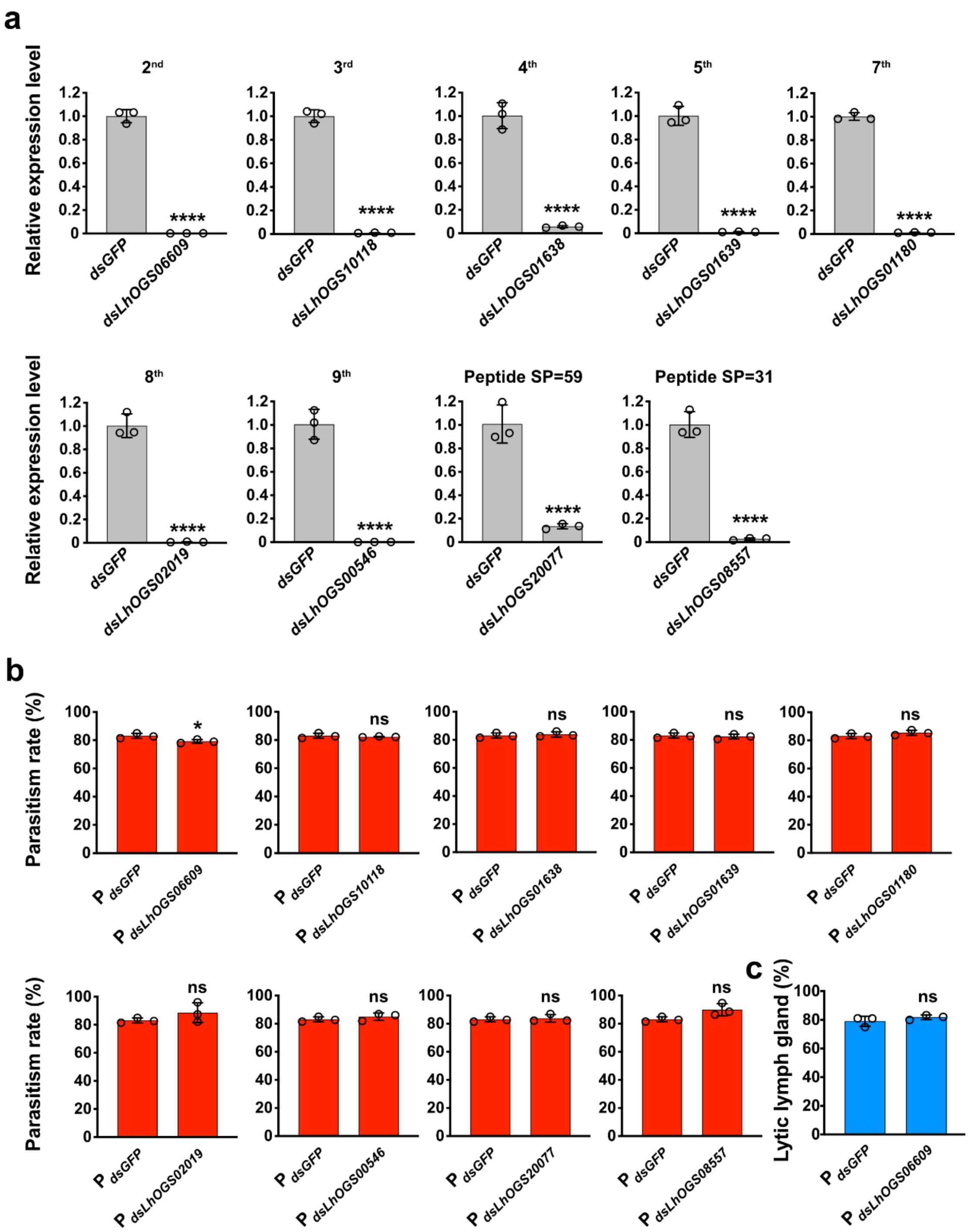

Supplementary Figure 16 RNAi and parasitic efficacy assays for other candidate venom proteins of high expression.

a Relative mRNA levels of *LhOGS06609*, *LhOGS10118*, *LhOGS01638*, *LhOGS01639*, *LhOGS01180*, *LhOGS02019*, *LhOGS00546*, *LhOGS20077*, and *LhOGS08557* in Lh after RNAi treatments. Three biological replicates were performed. Data are means ± SD; Significance was determined by two-tailed unpaired Student's t-test (*dsLhOGS06609*: *P*=6.2e-6; *dsLhOGS10118*: *P*=6.0e-6; *dsLhOGS01638*: *P*=0.0001; *dsLhOGS01639*: *P*=2.9e-5; *dsLhOGS01180*: *P*=7.7e-7; *dsLhOGS02019*: *P*=6.9e-5; *dsLhOGS00546*: *P*=0.0002; *dsLhOGS20077*: *P*=0.0007; *dsLhOGS08557*: *P*=0.0001). **b** Parasitism rate in host larvae after

parasitization by dsRNA-treated wasps in a. Three biological replicates were performed. Data are means ± SD; Significance was determined by two-tailed unpaired Student's t-test ($P_{dsLhOGS06609}$: P=0.0388; $P_{dsLhOGS10118}$: P=0.4854; $P_{dsLhOGS01638}$: P=0.604; $P_{dsLhOGS01639}$: P=0.633; $P_{dsLhOGS01180}$: P=0.1825; $P_{dsLhOGS02019}$: P=0.256; $P_{dsLhOGS00546}$: P=0.3684; $P_{dsLhOGS20077}$: P=0.7134; $P_{dsLhOGS08557}$: P=0.0651). **c** Percentage of host larvae exhibiting lytic lymph gland 24 h after parasitization by *dsGFP*-treated Lh (P_{dsGFP}, n=123), *dsLhOGS06609*-treated Lh ($P_{dsLhOGS06609}$, n=83). Three biological replicates were performed. Data are means ± SD; Significance was determined by two-tailed unpaired Student's t-test ($P_{dsLhOGS06609}$: P=0.2782). *:P < 0.05; ****: P < 0.001; ns: not significant.

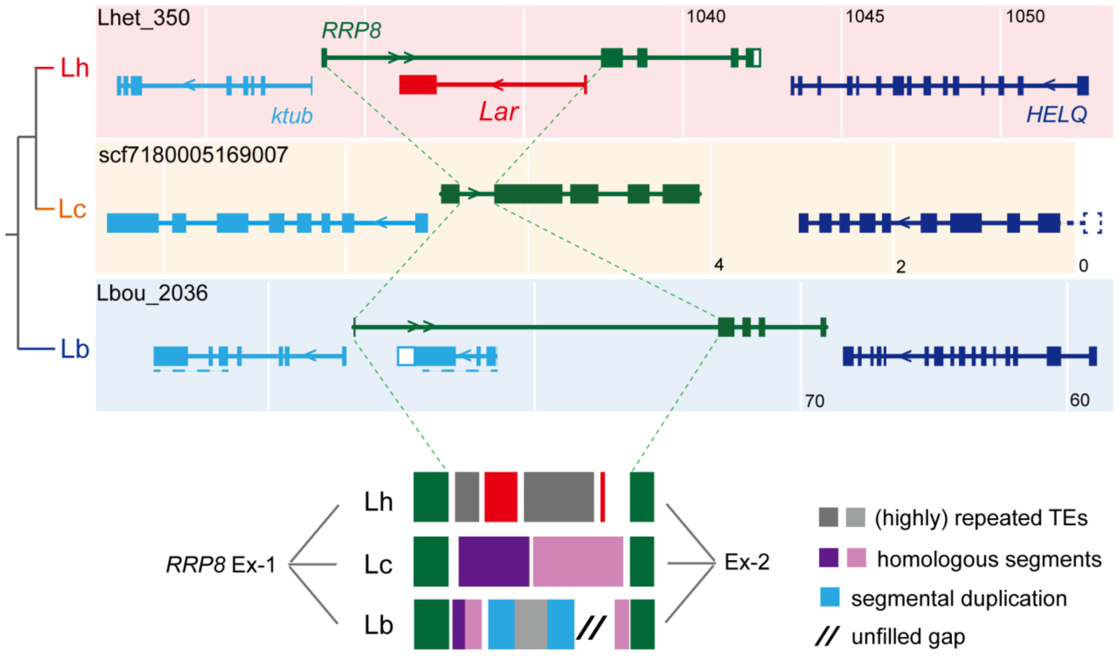

Supplementary Figure 17 Gene arrangement around the *Lar* locus across three *Leptopilina* species.

Orthologous genes were shown in the same color. Note that the first intron of HELQ (shown in the dashed box) is presented in another scaffold of the Lc genome. Numbers shown below the scale line (white) indicate genomic coordinates in Kb. *Lar* was reversely inserted into the first intron of *RRP8*, whose detailed information is shown below across three species. Note that the length of box is scaled to the proportion to the total length but the actual length. Red boxes indicate *Lar*, while others correspond to those noted on the right.

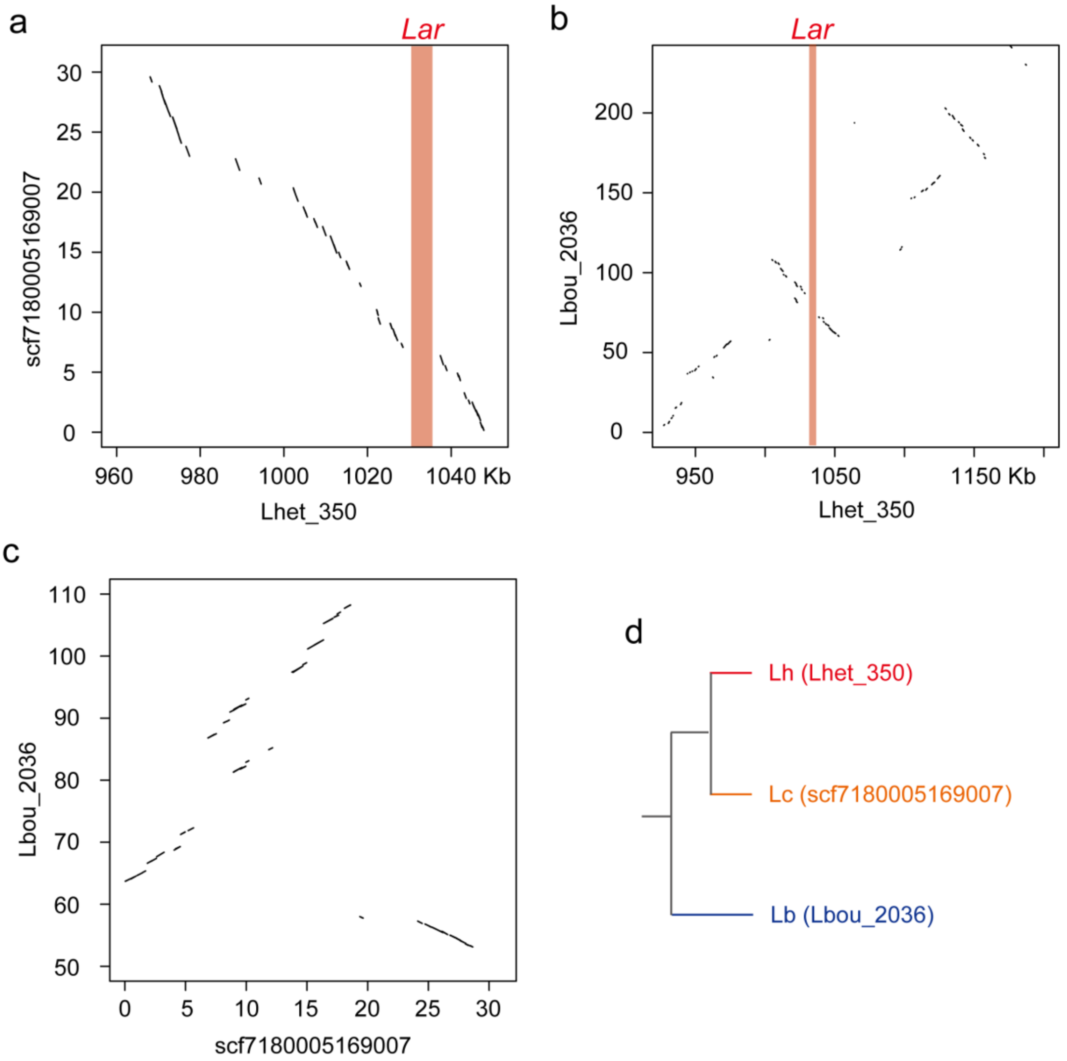

Supplementary Figure 18 Local synteny around the *Lar* locus across three *Leptopilina* species.

a Genome alignment between the genomes of Lh and Lc; **b** that between Lh and Lb; **c** that between Lc and Lb. **d** Phylogeny relationship among the three species. Shadows in red indicate the locus of Lar.

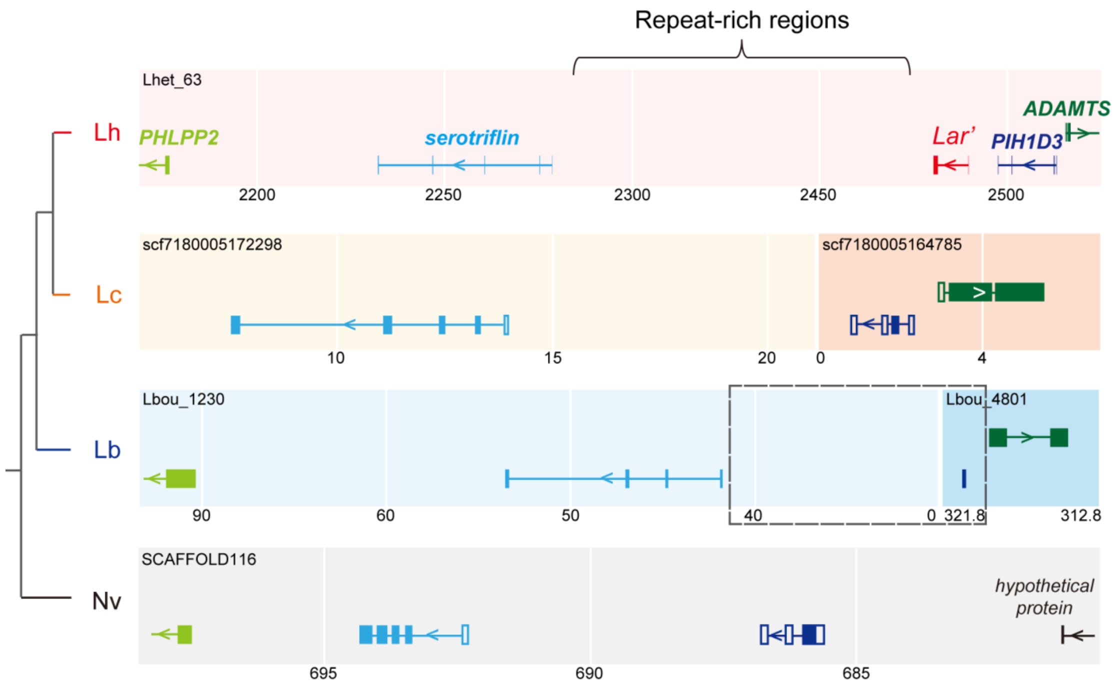

Supplementary Figure 19 Local synteny around the *Lar'* locus across three *Leptopilina* species and *N. vitripennis*.

Orthologous genes were shown in the same color. Unfilled exons in the same color indicate absence in the Lb genome. The grey dashed box outlined in places of Lh indicates that exons were massively lost in the first 42-Kb of Lbou_1230 and the last 4-Kb of Lbou_4801, which is homologous to the location of *Lar'* in Lh. The large region between serotriflin and *Lar'* is full of repeats. Note that some regions without any genic features were scaled up to fit the overall presentation; see numbers labeled below, indicating genomic coordinates in Kb, for detailed information.

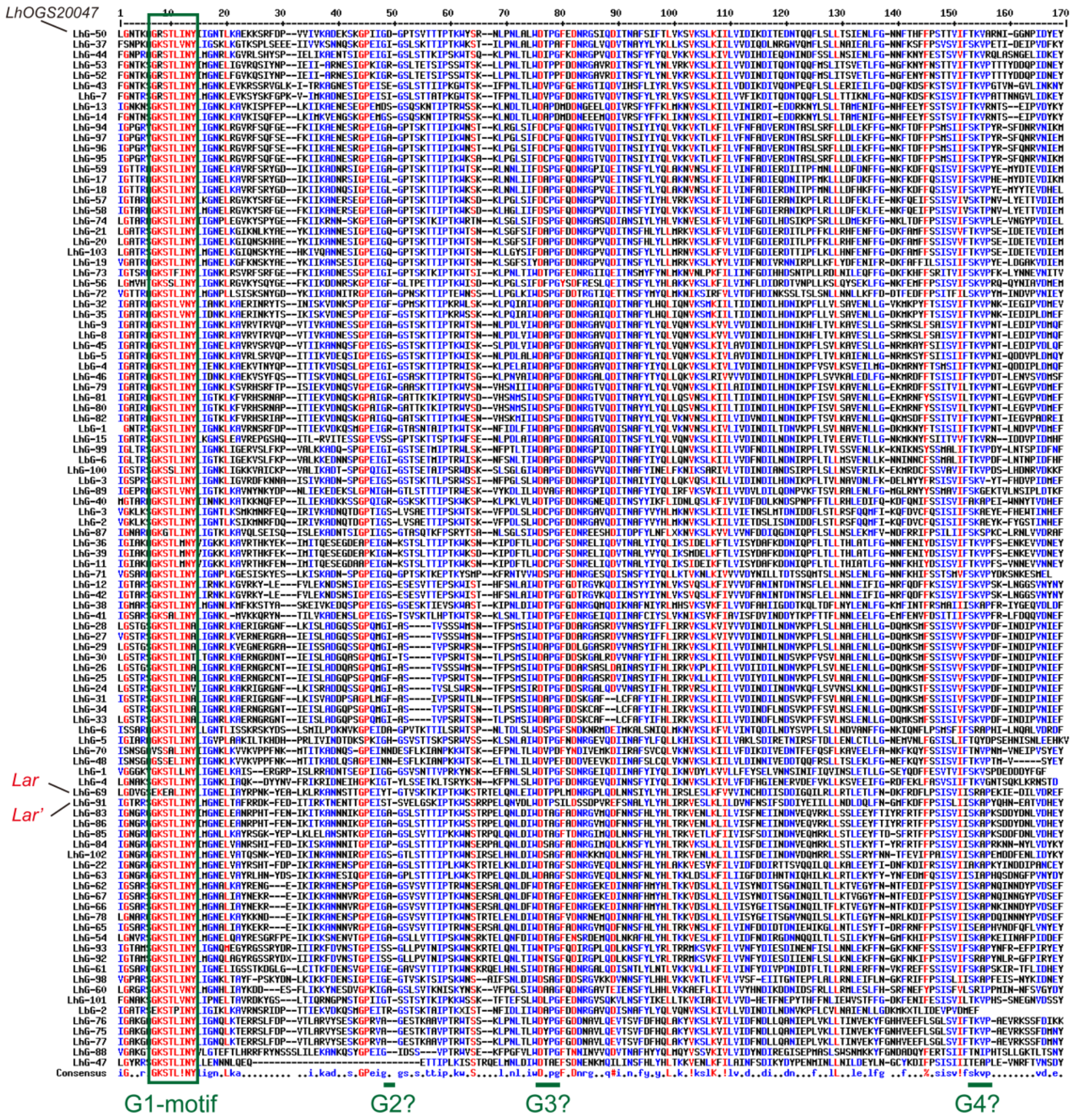

Supplementary Figure 20 Multiple alignment of all further homologs of *Lar* in the Lh and Lb genomes.

Only the 170-aa conserved region is shown. The four G motifs (in green) are referred to the previous study[3]. However, only G1-motif is conserved across homologs, as shown in a green box, and agrees with the previous prediction (GxxxGKS/T), while the other three G motifs are all poorly conserved and do not agree with the previous prediction[3] (G2: T; G3: DxxG; G4: T/SKVP).

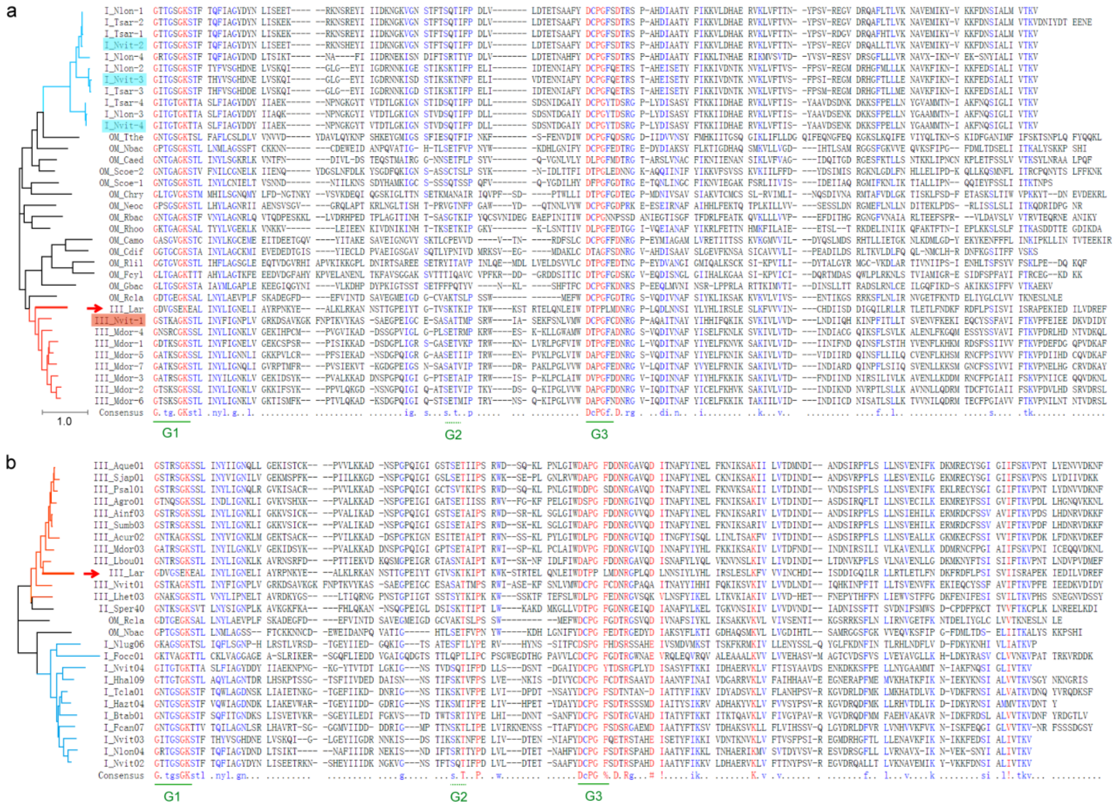

Supplementary Figure 21 Multiple alignment of representative homologs of *Lar*.
a Multiple alignment of *Lar* homologs in Chalcidoidea. **b** Multiple alignment of representative *Lar* homologs across different subgroups. The phylogenetic trees were independently calculated based on the multiple alignment using MEGA7[4] under the JTT model. G-motif sites correspond to those in Supplementary Fig. 20. Species initiated with I, II, III, and OM indicate copies from groups I, II, and III and outgroup as shown in Fig. 3. Species in shadow indicate copies of *N. vitripennis*. Note that *N. vitripennis* is the only species that simultaneously encodes copies of clades I and III.

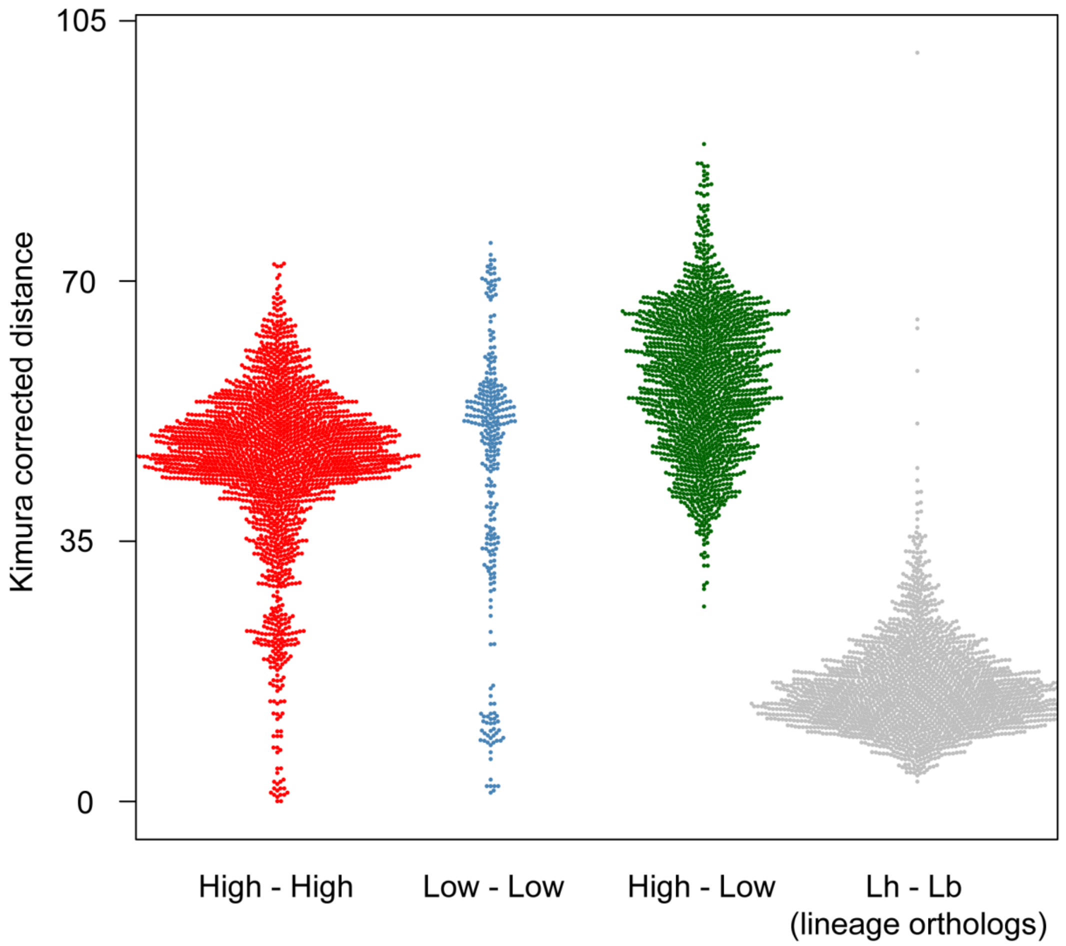

Supplementary Figure 22 Pairwise divergence across all *Lar* homologs.
Each dot indicates the genetic distance (*K* distance) between two homologs from the respective group. All homologs were subdivided into two groups based on the expression level in VG. As control, a total of 2492 co-lineage orthologous gene pairs between Lh and Lb were identified.

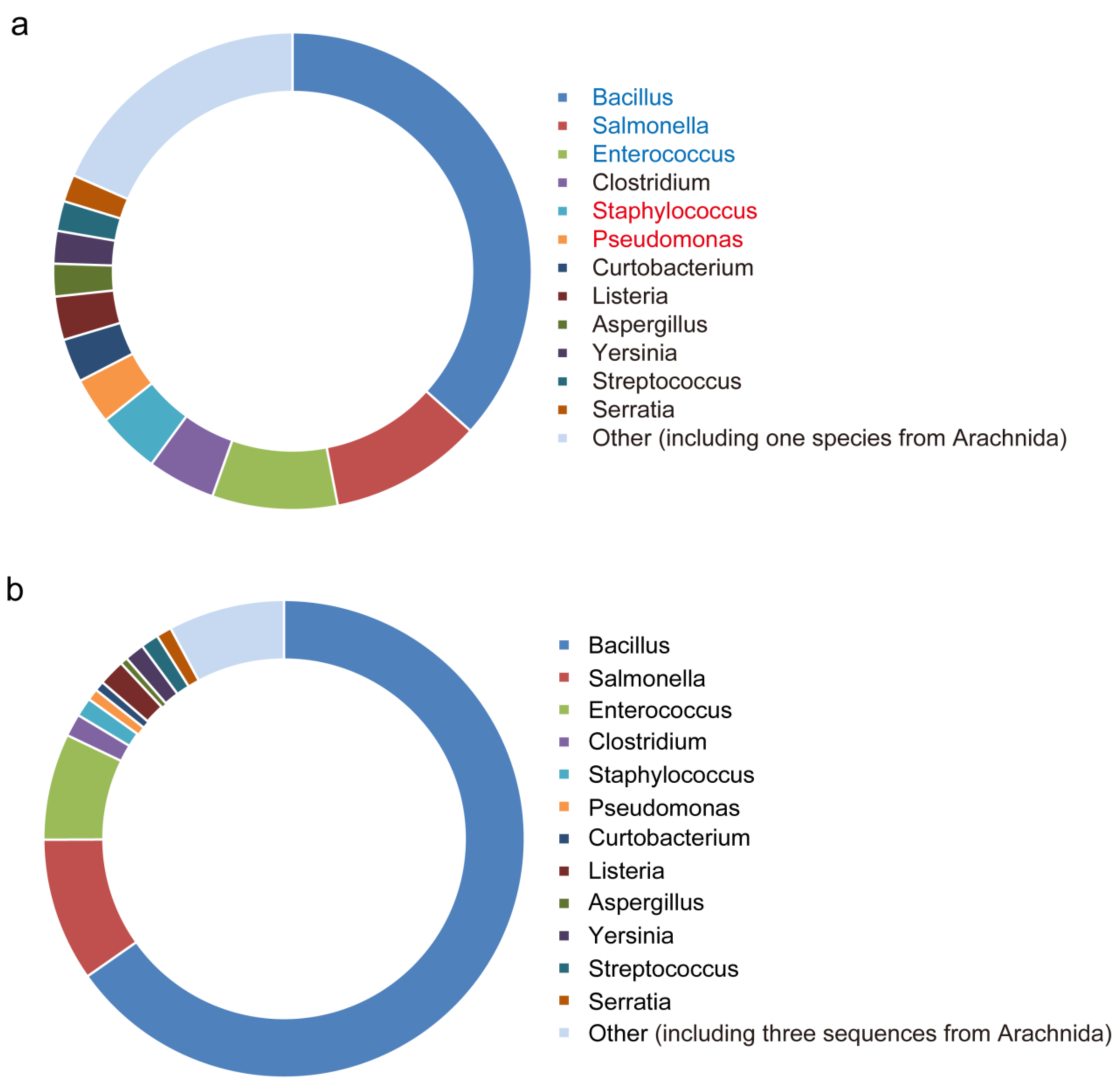

Supplementary Figure 23 Distribution of taxa documented with IPR004954 in the InterPro database.

a The pie chart showing proportion of accumulated species within each genus being documented with IPR004954. Genus names in red indicate considerable presence in the microbiota sequencing of Lb, while those in blue indicate a little presence (Supplementary Data 9). **b** The pie chart showing proportion of accumulated sequences within each genus being documented with IPR004954.

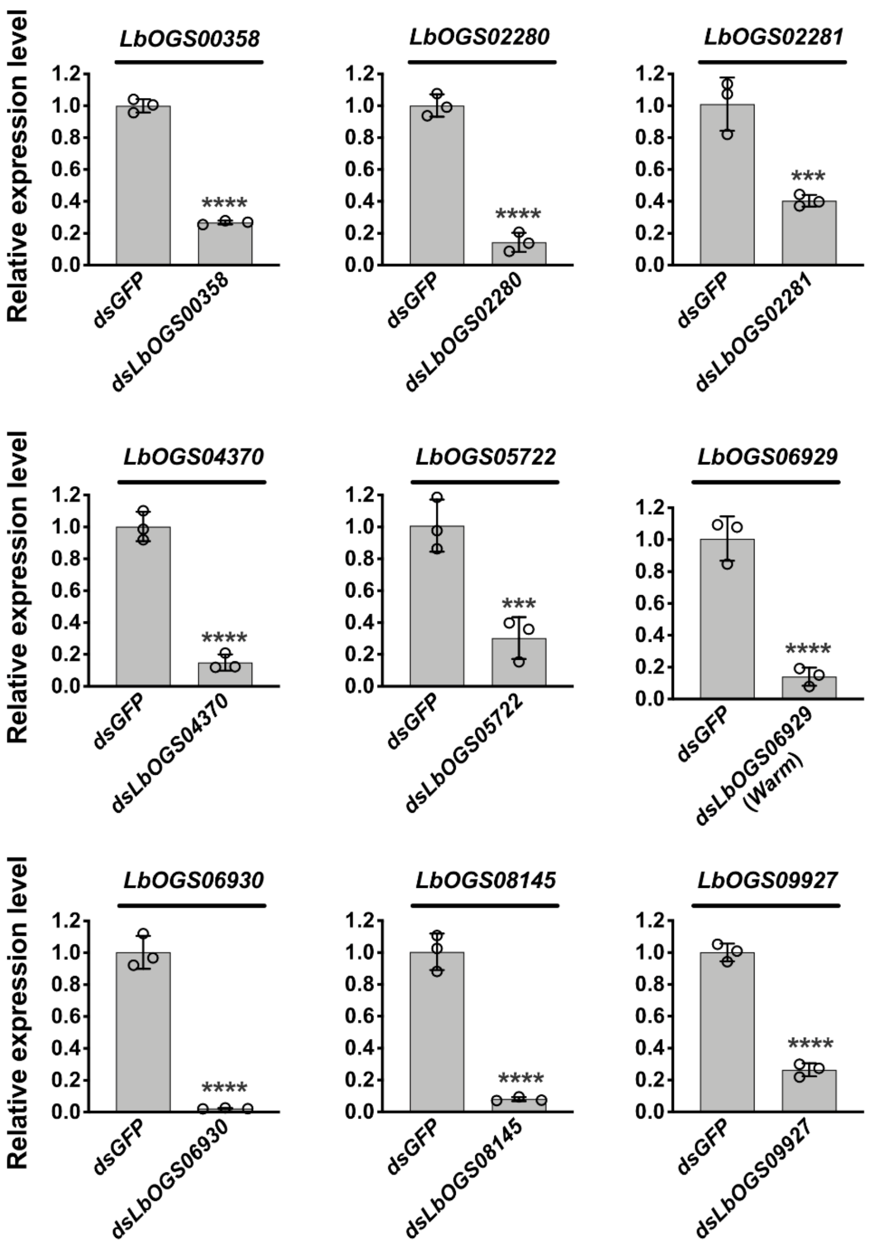

Supplementary Figure 24 RNAi efficacy for 9 mucin-binding domain-containing genes in Lb.

Relative mRNA levels of *LbOGS00358, LbOGS02280*, *LbOGS02281*, *LbOGS04370*, *LbOGS05722*, *LbOGS06929* (*Warm*), *LbOGS06930*, *LbOGS08145* and *LbOGS09927* in *L. boulardi* after RNAi treatments. Three biological replicates were performed. Data are means ± SD; Significance was determined by two-tailed unpaired Student's t-test (*dsLbOGS00358*: *P*=7.9e-6; *dsLbOGS02280*: *P*=7.5e-5; *dsLbOGS02281*: *P*=0.0048; *dsLbOGS04370*: *P*=0.0001; *dsLbOGS05722*: *P*=0.003; *dsLbOGS06929*: *P*=0.0007; *dsLbOGS06930*: *P*=8.1e-5; *dsLbOGS08145*: *P*=0.0002; *dsLbOGS09927*: *P*=3.3e-5). ***: $P < 0.005$; ****: $P < 0.001$.

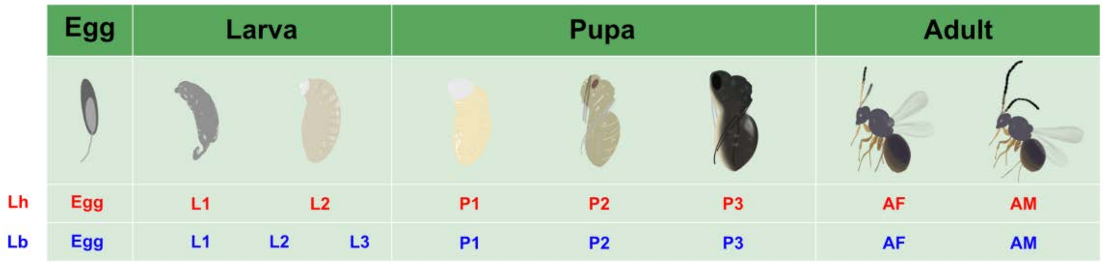

Supplementary Figure 25 Different developmental stages of *Leptopilina* species for transcriptome analysis.

Eight samples of Lh and nine samples of Lb were collected for the transcriptome sequence. E, eggs; L1, days 1-3 larvae; L2, days 4-9 larvae for Lh while days 4-6 larvae for Lb; L3, days 7-9 larvae for Lb; P1, days 1-3 pupae; P2, days 4-7 pupae; P3, days 8-10 pupae; AF, female adults; AM, male adults.

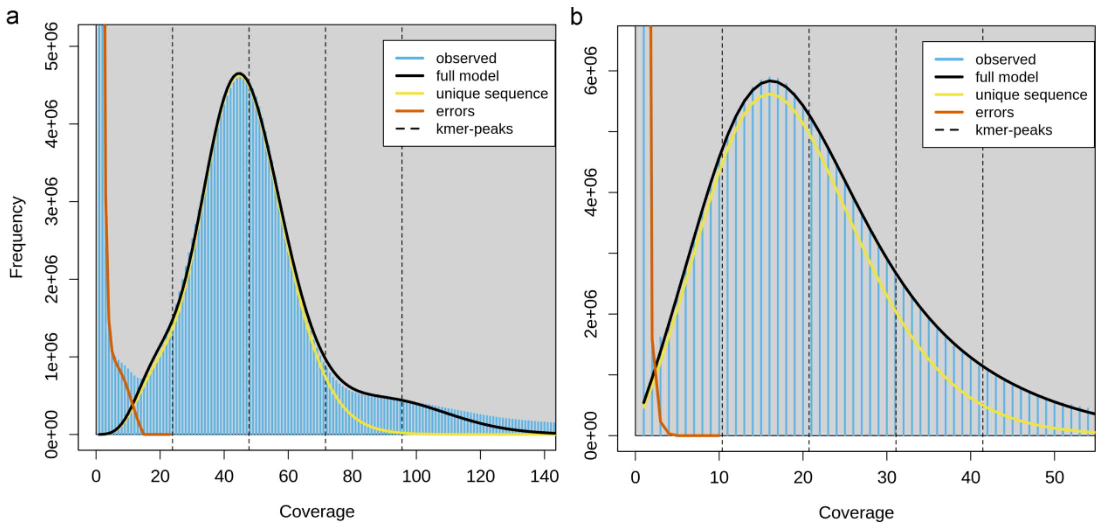

Supplementary Figure 26 K-mer distribution across the Lh and Lb genomes.
a the 17-mer distribution across the Lh genome. **b** the 17-mer distribution across the Lb genome. The plot was generated using GenomeScope (github.com/schatzlab/genomescope).

Supplementary Table 1 Sequencing statistics of the Lh genome

Sequencing mode	Insert size	Read length (N50)	Mean read length	#Reads	Total length (Gb)	Coverage (x)
Pacbio Sequel	20 Kb	15,813	9,144	3,395,220	31.05	63.8
Illumina Paired-end		150	150	80,048,286×2	24.01	49.3

Supplementary Table 2 Basic features of the assembled genomes of Lh and Lb

	Leptopilina heterotoma	*Leptopilina boulardi*
Genome assemble		
Assembly size (bp)	487,015,184	323,668,388
#Scaffolds	411	9,872
Scaffold N_{50} (bp)	2,183,205	458,625
Contig size (bp)	487,015,184	299,861,858
#Contigs	411	38,756
Contig N_{50} (kp)	2,183,205	14,385
Gene annotation		
Protein-coding	11,881	11,054
Genomic features		
GC (%)	26.94	25.77
Coding (%)	4.9	8.8
Quality control		
BUSCO partial (%)	98.7	97.2
BUSCO complete (%)	98.5	95.1
CEGMA partial (%)	98.4	97.6
CEGMA complete (%)	90.7	94.4

Supplementary Table 3 Sequencing statistics of the Lb genome

Library mode	Insert size	Read length (bp)	#Read pairs	Total length (Gb)	Coverage (x)
Paired-end	180 bp	125	25,564,639	6.39	21.3
Paired-end	300 bp	125	37,145,732	9.29	31.0
Paired-end	450 bp	250	22,375,641	11.19	37.3
Mate-pair	2 Kb	125	26,047,574	6.51	21.7
Mate-pair	3 Kb	125	13,822,559	3.46	11.5
Mate-pair	5 Kb	125	14,178,383	3.54	11.8
Mate-pair	8 Kb	125	12,480,118	3.12	10.4
Mate-pair	13 Kb	125	30,459,170	7.61	25.4

Supplementary Table 4 Statistics of repeat content in the Lh and Lb genomes

Species	*Leptopilina heterotoma*		*Leptopilina boulardi*	
Class	Length (bp)	% Genome	Length (bp)	% Genome
Retroelements	23,063,394	4.74%	11,006,358	3.40%
SINEs:	2,064,383	0.42%	284,727	0.09%
Penelope	520,623	0.11%	173,681	0.05%
LINEs:	11,050,478	2.27%	4,910,145	1.52%
L2/CR1/Rex	587,532	0.12%	533,853	0.16%
R1/LOA/Jockey	2,916,696	0.60%	559,000	0.17%
R2/R4/NeSL	40,952	0.01%	26,216	0.01%
RTE/Bov-B	50,075	0.01%	16,120	0.00%
L1/CIN4	15,487	0.00%	7,606	0.00%
LINE1	0	0.00%	58,153	0.02%
LINE2	1,214,680	0.25%	201,338	0.06%
L3/CR1	253,782	0.05%	1,921,501	0.59%
LTR elements:	9,948,533	2.04%	5,811,486	1.80%
BEL/Pao	1,099,859	0.23%	728,983	0.23%
ERVL	15,284	0.00%	0	0.00%
ERV_classI	19,889	0.00%	0	0.00%
ERV_classII	46,697	0.01%	0	0.00%
Ty1/Copia	1,069,378	0.22%	791,645	0.24%
Gypsy/DIRS1	4,952,234	1.02%	2,649,768	0.82%
DNA transposons	37,457,347	7.69%	15,184,067	4.69%
Hat-Charlie	37,481	0.01%	293,139	0.09%
TcMar-Tigger	32,129	0.01%	35,390	0.01%
hobo-Activator	169,427	0.03%	80,763	0.02%
Tc1-IS630-Pogo	165,140	0.03%	113,204	0.03%
PiggyBac	8,634	0.00%	11,204	0.00%
Tourist/Harbinger	15,502	0.00%	19,605	0.01%
Other	42,426	0.01%	30,013	0.01%
Unclassified:	190,157,436	39.05%	83,172,397	25.70%
Total	250,678,177	51.47%	109,362,822	33.79%

Supplementary Table 5 Official gene sets of Lh and Lb

	Lh	Lb
Approach for gene prediction		
Maker (Consensus) #genes	11,864	11,013
Braker #genes	38,798	22,202
StringTie #genes	13,641	13,645
ToFU #genes	7246	9815
OGS (further filtered based on Maker)	11,881	11,054
Mean exon per gene	6.2	6.6
Median exon per gene	5	5
Mean exon length (bp)	289	352
Mean intron length (bp)	2356	1841
Genes w/ annotations		
Transcriptome evidence	11,079	10,574
Hymenopteran homology	11,259	10,087
Drosophila homology	8973	8094
KEGG KO	7697	6891
Gene Ontology	6626	5910
InterPro domain	9473	8472
Pfam domain	8884	7947
NCBI RefSeq (invertebrate)	11,323	10,158
UniProt (Hymenoptera)	11,271	10,079

Supplementary Table 6 Statistics of RNAseq data in this study

Sample ID	Sample information	RNAseq data (Gb)	#Expressed genes	#Highly expressed genes
Lh				
E	Egg	7	6245	143
L1	Larva (day 1-3)	6.8	6485	138
L2	Larva (day 4-9)	6.4	5795	98
P1	Pupa (day 1-3)	7	6602	69
P2	Pupa (day 4-7)	6.7	6375	94
P3	Pupa (day 8-10)	6.8	7028	138
AF	Female adult	7.3	6113	170
AM	Male adult	6.9	6819	71
VG	Venom glands	7.3	4274	39
	Pooled[a]	8.6	6184	-
Lb				
Egg	Egg	7.4	5511	103
L1	Larva (day 1-3)	7.7	6374	126
L2	Larva (day 4-6)	7.4	4975	102
L3	Larva (day 7-9)	8.6	4593	92
P1	Pupa (day 1-3)	9.1	6287	111
P2	Pupa (day 4-7)	7	6372	108
P3	Pupa (day 8-10)	6.9	5898	79
AF	Female adult	7.2	5602	128
AM	Male adult	7.2	6229	84
VG	Venom glands	7.3	4538	28
	Pooled[a]	22	8459	-

[a]This sample was pooled from independent samples above equally and subject for full-length RNAseq using the PacBio platform. We mainly used for gene prediction but expression quantification.

Supplementary Table 7 BLASTP of Warm homologs

Gene ID	E-value	Query cover	Score	Best BLASTP hit (Accession ID)	Best BLASTP hit genus	IPR004954 (InterProScan)
LbOGS02281	4e-20	86%	107	WP_142464257	Klebsiella	504-613(1.5e-9)
LbOGS04370	3e-16	80%	95.5	WP_142466376	Klebsiella	551-660 (6.7e-11)
LbOGS09927	6e-7	38%	66.2	WP_079940407	Paenibacillus	542-650 (4.2e-8)<br>670-776 (1.9e-6)<br>798-908 (1.9e-8)
LbOGS06929	1e-5	13%	60.8	WP_050103721	Yersinia	861-970 (1.6e-9)
LbOGS02280	0.003	16%	53.5	WP_039946932	Anaerostipes	693-802 (7.8e-10)
LbOGS05722	0.017	57%	46.2	TCW59216	Bacillus	57-165 (3.5e-11)
LbOGS00358	0.051	31%	45.4	VEA39472	Salmonella	176-286 (7.4e-10)
LbOGS06930	0.13	35%	48.9	WP_051492772	Listeria	717-824 (3e-7)<br>868-970 (5.8e-7)
LbOGS08145	0.1	10%	48.9	ECQ2803623	Salmonella	542-650 (9.4e-8)<br>670-776 (1.9e-6)<br>798-906 (1.1e-8)

Supplementary References

[1] Corpet, F. Multiple sequence alignment with hierarchical clustering. *Nucleic Acids Res.* **16**, 10881-10890 (1988).

[2] Kelley, L., Mezulis, S., Yates, C. et al. The Phyre2 web portal for protein modeling, prediction and analysis. *Nat. Protoc.* **10**, 845-858 (2015).

[3] Heavner, M. E. et al. Novel organelles with elements of bacterial and eukaryotic secretion systems weaponize parasites of *Drosophila*. *Curr. Biol.* **27**, 2869-2877 (2017).

[4] Kumar, S., Stecher, G. & Tamura, K. MEGA7: molecular evolutionary genetics analysis version 7.0 for bigger datasets. *Mol. Biol. Evol.* **33**, 1870-1874 (2016).
