# Supplementary material for: Two novel venom proteins underlie divergent parasitic strategies between a generalist and a specialist parasite
Source: Nat Commun. 2021 Jan 11;12:234. doi: 10.1038/s41467-020-20332-8 (PMC7801585; doi:10.1038/s41467-020-20332-8)
Supplement: Supplementary file 3 — Reporting Summary [file 41467_2020_20332_MOESM3_ESM.pdf]

nature research

Corresponding author(s): Jianhua Huang; Xuexin Chen; Shuai Zhan

Last updated by author(s): Nov 9, 2020

Reporting Summary

Nature Research wishes to improve the reproducibility of the work that we publish. This form provides structure for consistency and transparency in reporting. For further information on Nature Research policies, see our Editorial Policies and the Editorial Policy Checklist.

Statistics

For all statistical analyses, confirm that the following items are present in the figure legend, table legend, main text, or Methods section.

n/a	Confirmed	
☐	☒	The exact sample size (*n*) for each experimental group/condition, given as a discrete number and unit of measurement
☐	☒	A statement on whether measurements were taken from distinct samples or whether the same sample was measured repeatedly
☐	☒	The statistical test(s) used AND whether they are one- or two-sided<br>*Only common tests should be described solely by name; describe more complex techniques in the Methods section.*
☒	☐	A description of all covariates tested
☐	☒	A description of any assumptions or corrections, such as tests of normality and adjustment for multiple comparisons
☐	☒	A full description of the statistical parameters including central tendency (e.g. means) or other basic estimates (e.g. regression coefficient) AND variation (e.g. standard deviation) or associated estimates of uncertainty (e.g. confidence intervals)
☐	☒	For null hypothesis testing, the test statistic (e.g. *F*, *t*, *r*) with confidence intervals, effect sizes, degrees of freedom and *P* value noted<br>*Give P values as exact values whenever suitable.*
☒	☐	For Bayesian analysis, information on the choice of priors and Markov chain Monte Carlo settings
☒	☐	For hierarchical and complex designs, identification of the appropriate level for tests and full reporting of outcomes
☒	☐	Estimates of effect sizes (e.g. Cohen's *d*, Pearson's *r*), indicating how they were calculated

Our web collection on statistics for biologists contains articles on many of the points above.

Software and code

Policy information about availability of computer code

Data collection	Zeiss Zen was used to collect the confocal images.
Data analysis	k-mer analysis: Jellyfish;<br>Genome assembly (long reads): Canu (removing errors), SMARTdenovo (assembling), pilon (polish);<br>Genome assembly (Illumina reads): Platanus (reads trimming), DiscovarDeNovo (assembling contigs), ScaffMatch (scaffolding), GapCloser (gap filling), Rabbit (removing redundancy);<br>Assembly assessment: CEGMA, BUSCO;<br>Repeat annotation: RepeatModeler (generating species-specific library), RepeatMasker;<br>Gene prediction: Maker (combining signatures), AUGUSTUS (providing ab initio signatures), SNAP (providing ab initio signatures), cDNA_Cupcake (processing PacBio RNAseq data), StringTie (processing Illumina RNAseq data);<br>Gene annotation: BLASTP (alignming genes to other gene sets or databases), InterProScan (mapping genes with functional terms);<br>Gene prediction of specific gene families (such as chemoreception receptor genes): TBLASTN (mapping homologs to the genome), GeneWise (predicting genes based on homology);<br>3D modeling: Phyre2;<br>Orthology analysis: OrthoFinder (sorting orthology), MUSCLE (multiple alignment), Gblocks (retrieving conserved blocks);<br>Phylogenomic analysis: RAxML;<br>Phylogenetic analysis: RAxML, MEGA7;<br>Divergence timing: R8s;<br>Divergence rate: PAML (dN/dS);<br>Expression profiling: Salmon;<br>PCA analysis: prcomp of R;<br>Heatmap and clustering: pheatmap of R;

Enrichment of highly expressed genes: phyper of R;
Pathway enrichment analysis: OMICSHARE;
Local synteny: MUMmer;
Pairwise divergence between genes: distmat of EMBOSS;
Microbiota analyses: SeqPrep (removal of adapters), Sickle (trimming reads), BWA (mapping reads to remove host DNA), Megahit (assembling reads), CD-HIT (cluster assembled reads), BLASTP (mapping reads to NR).

For manuscripts utilizing custom algorithms or software that are central to the research but not yet described in published literature, software must be made available to editors and reviewers. We strongly encourage code deposition in a community repository (e.g. GitHub). See the Nature Research guidelines for submitting code & software for further information.

Data

Policy information about availability of data

All manuscripts must include a data availability statement. This statement should provide the following information, where applicable:

- Accession codes, unique identifiers, or web links for publicly available datasets
- A list of figures that have associated raw data
- A description of any restrictions on data availability

The transcript sequences of Lar and Warm were deposited in GenBank with the accession numbers MT431620 (Lar) and MT439843 (Warm). Genomes and transcriptome sequencing data was deposited in GenBank BioProject under accession numbers PRJNA624738 (Lh) and PRJNA624743 (Lb). The proteome data of the venom fluids was deposited in PeptideAtlas under the accession number PASS01574. The microbiota sequencing data was deposited in GenBank BioProject under the accession number PRJNA629859. The authors declare that all data supporting the findings of this study are available within the paper and its supplementary information files or from the corresponding author upon request. Raw data of Figures 1, 2, 4 and Supplementary Figures 9, 12,13, 16, 24 are provided as a Source Data file. Source data are provided with this paper.

Field-specific reporting

Please select the one below that is the best fit for your research. If you are not sure, read the appropriate sections before making your selection.

☒ Life sciences ☐ Behavioural & social sciences ☐ Ecological, evolutionary & environmental sciences

For a reference copy of the document with all sections, see nature.com/documents/nr-reporting-summary-flat.pdf

Life sciences study design

All studies must disclose on these points even when the disclosure is negative.

Sample size	DNA of at least 2000 male wasp adults was extracted for each group. Transcriptional levels of wasp samples were measured per development stage. Approximately 300 VRs of 3-d-old Lh female wasps were collected for LC-MS/MS analysis. Total of 30 wasp adults and 150 wasp midguts were dissected for microbiota sequencing per group. More than 100 host larvae were used to test the wasp oviposition rate for each group. More than 500 hosts were used to test the encapsulation rate, parasitism rate and wasp emergence rate for each group. More than 100 host lymph gland were used to test the lytic percentage for each group. More than 75 host larvae were used to test the wasp egg attaching rate for each group. At least 50 wasps were micro-injected with dsRNA for each gene. 20 animals were used to do the qRT-PCR experiments per group. Apoptosis detection was carried out using at least 30 host larval lymph glands. 40 wasp venom apparatus and the carcass were collected for western blot analysis. At least 20 host larval lymph glands were used for immunohistochemistry per group. These numbers of samples were sufficient to perform a confident data analysis. All the n values are provided in legends and source data.
Data exclusions	No data were excluded.
Replication	The experiments for detecting the wasp oviposition rate, parasitism rate and egg attaching rate were done at least for three times. The qRT-PCR experiment for detecting RNA interference effeciency of each gene was repeated at least for three times.
Randomization	The samples for DNA sequencing, transcriptome sequencing, LC-MS, microbiota analysis, western blot and qRT-PCR were all randomly picked. The host larvae were also randomly picked to test the wasp oviposition rate, encapsulation rate, parasitism rate, wasp emergence rate, and wasp egg attaching rate. The Lymph Glands dissected from a certain number of randomly-selected parasitized host larvae for detect the lytic percentage and antibody staining.
Blinding	Investigators were blinded to group allocation for the quantification of the wasp egg attaching rate.

Reporting for specific materials, systems and methods

We require information from authors about some types of materials, experimental systems and methods used in many studies. Here, indicate whether each material, system or method listed is relevant to your study. If you are not sure if a list item applies to your research, read the appropriate section before selecting a response.

Materials & experimental systems

n/a	Involved in the study
☐	☒ Antibodies
☒	☐ Eukaryotic cell lines
☒	☐ Palaeontology and archaeology
☐	☒ Animals and other organisms
☒	☐ Human research participants
☒	☐ Clinical data
☒	☐ Dual use research of concern

Methods

n/a	Involved in the study
☒	☐ ChIP-seq
☒	☐ Flow cytometry
☒	☐ MRI-based neuroimaging

Antibodies

Antibodies used

anti-Lar (ABclonal, rabbit polyclonal, 1:1000)
anti rabbit IgG, HRP (Solarbio, Cat#SE134, from goat serum, 1:2000)
Alexa Fluor 488 Goat anti-Rabbit second antibody (Invitrogen, Cat#A11008, 1:1000)
Alexa Fluor 594 Goat anti-Rabbit second antibody (Invitrogen, Cat#A11012, 1:1000)
ProLong Gold Antifade Mountant with DAPI (Invitrogen, Cat#P36935)

Validation

anti-Lar: reactivity validated by the company for generating antibodies
anti rabbit IgG, HRP: reactivity validated by the company for Drosophila
Alexa Fluor 488 Goat anti-Rabbit second antibody: reactivity validated by the company for Drosophila
Alexa Fluor 594 Goat anti-Rabbit second antibody: reactivity validated by the company for Drosophila
ProLong Gold Antifade Mountant with DAPI: reactivity validated by the company for Drosophila

Animals and other organisms

Policy information about studies involving animals; ARRIVE guidelines recommended for reporting animal research

Laboratory animals

Drosophila and wasps were obtained from non-profit stock centers or from other colleagues.

Wild animals

Not applicable in this study.

Field-collected samples

Not applicable in this study.

Ethics oversight

Not applicable in this study.

Note that full information on the approval of the study protocol must also be provided in the manuscript.
