# Supplementary material for: Two novel venom proteins underlie divergent parasitic strategies between a generalist and a specialist parasite
Source: Nat Commun. 2021 Jan 11;12:234. doi: 10.1038/s41467-020-20332-8 (PMC7801585; doi:10.1038/s41467-020-20332-8)
Supplement: Supplementary file 4 — Description of Additional Supplementary Files [file 41467_2020_20332_MOESM4_ESM.pdf]

Description of Additional Supplementary Files

Supplementary Data 1	Information of dN/dS in pathways
Supplementary Data 2	Lh specific genes
Supplementary Data 3	Lb specific genes
Supplementary Data 4	Lh venom protein-coding genes (VP)
Supplementary Data 5	Homologs of Lar across the Lh and Lb geomes
Supplementary Data 6	Annotated IPR domains across hymenopteran genomes
Supplementary Data 7	Documented PF03272 in Pfam
Supplementary Data 8	Documented IPR004954 in InterPro
Supplementary Data 9	Genus abundance of Lb microbiota sequencing
Supplementary Data 10	Assembly statistics of available Leptopilina genomes
Supplementary Data 11	Hexpoda genomes used in this study to search Lar homologs
Supplementary Data 12	Primer sequences used in this study
