# Supplementary material for: Two novel venom proteins underlie divergent parasitic strategies between a generalist and a specialist parasite
Source: Nat Commun. 2021 Jan 11;12:234. doi: 10.1038/s41467-020-20332-8 (PMC7801585; doi:10.1038/s41467-020-20332-8)
Supplement: Supplementary file 2 — Peer Review File [file 41467_2020_20332_MOESM2_ESM.pdf]

Reviewer #1 (Remarks to the Author):

The authors of “Two horizontal gene transfers underlie divergent parasitic strategies between a generalist and a specialist parasite” look at several venom protein genes in the parasitoid wasps Leptopilina heterotoma (Lh) and L. boulardi (Lb) to determine their function and origin. The paper determines the function of Lh Lar, which triggers the lysis of the Drosophila lymph gland leading to the inhibition of the host encapsulation response, and Lb Warm, which assists in the attachment of wasp eggs to the internal organs of the host, providing physical protection against the complete encapsulation that helps parasites escape from the host cellular immune response. How these genes contribute to the wasp’s host range it also assessed. The paper further discusses the possible LGT origin of Lar and another gene mucin-db.

This is a very interesting study and I really enjoyed reading the manuscript. The paper is very well written and flows nicely. The analyses are thorough, and the conclusions are well supported. The figures are well put together and clearly illustrate the data. The only issue I had with the paper is the section of the LGT origins of Lar.

You didn’t convince me that Lar is an LGT in this section. Lar is undoubtably something very strange in the Lh and Lb genomes, but it would be unlike any other reported LGT before. First, you report that all the initial hits were only 20-30% sequence ID, which with such a short sequence leaves me to wonder if they are in fact really homologs of each other or just spurious BLAST hits. The iterative BLASTN-GeneWise approach further makes me worried if these genes are truly homologs or it just a chain of weak support strung together. What is the percent sequence id between the chalcid clade I and III and cynipid clade III Lar sequences? Adding some representative sequences from the other groups to Sup. Fig. 18 would help add support to you claim (Also Sup. Fig. 18 is mislabeled Sup. table 18 on line 852). You can also compare the predicted structure of these proteins (in PhyreII or something similar) to further add support to your conclusions that these are true homologs.

The other oddity of this is that there is no clear origin for this gene. Most LGTs are common in their clade of origin, for example the prevalence of mucin-bd gene in bacteria. There is no indication of where Lar originated and how it was exposed to Lh and Lb genomes to make the transfer. Let alone how it did all the other jumps (which should be increased from 3 to at least 8 because of the amount of losses it would take to correlate the Lar clades to the species tree). The fact that Clades I-III aren’t nested within microorganisms needs to be addressed as well, does it suggests the gene is jumping directly from insect to insect or does the eukaryotic evolution of the gene make the appear more similar? For this story to be convincing you need to propose a hypothesis of how it moved from group to group. Do you think it has a viral origin? Is it a TE? Is parasitoid venom spreading it? Do all the genomes that have copies of Lar have extremely low GC content?

This paper is very interesting with very strong support for all your other conclusions, including that mucin-db gene is an LGT. I would suggest maybe not including the LGT story in the title of your paper and focusing more on your very solid other results, with the LGT an interesting but slightly confusing side story.

Minor comments:
-line 95: For the 16% of GC windows in Lh with extremely low GC content, are the windows scatter throughout the genome or grouped? Do they correspond to repetitive elements, centromeres, or any other genomic architecture?
Line 115: With an estimated 40 MY divergence time and only ~86% sequence identity, I would be very hesitant to call Lh and Lb sister species. For example, two sister species in the Nasonia clade are only separated by 0.5MY with 98% sequence identity. These two species seem to have a lot of evolution between them.

Lines 141-149: The PCA analysis in SupFig 5 does not support your conclusions. The PCA (which I

would trust more than the cluster next to your expression heat map) shows that the life stages of Lh and Lb are quite similar in their expression, with the exception of P3.

Line 214: Sorry if I missed it, but I didn't see what bioinformatic analyses were tried to predict functions and domains in Lar. Might be good just to list some analyses. Did you try determining a function from a predicted protein structure, for example PhyreII?

Fig 3a have an arrow to Lar-Lar' on phylogeny as well as expression map.

Missing or mislabeled supplemental tables

Reviewer #2 (Remarks to the Author):

The authors have combined multiomic data, evolutionary analyses, parasitic efficiency assays and functional experiments to identify two horizontal gene transfers underlying divergent parasitic strategies between a generalist (Lh) and a specialist (Lb) parasite. The Lh-specific protein, Lar, enables active immune suppression by lysing the Drosophila lymph glands, eventually leading to successful parasitism by Lh. Meanwhile, the Lb-specific protein, Warm, may contribute to a passive strategy by attaching the laid eggs to the gut and other internal organs of the host, leading to incomplete encapsulation and helping Lb escape the host cellular immune response. The results provide an important reference for the in-depth study of the molecular mechanisms of different parasitic strategies between the generalist Lh and the specialist Lb.

Major points:
1. LhOGS20047 has a high level of protein expression and evidence of RNAi, but does not affect the efficiency of parasitism of Lh. Among this gene family, which sequence feature of Lar play a key role for parasitism function?
2. In the proteome study of Lh, the genes with the highest protein expression level are not lar and lar'. I wonder if those highest expressed proteins also have an effect on parasitic efficiency and thus parasitic function is contributed by a variety of venom proteins?
3. The assembly quality of the genomes of these two species is very different. The annotated gene numbers differ by more than 800. These differences may affect the accuracy of certain gene categories of Supplementary Figure 3. It would be better to discuss this in the paper.
4. Line 392. The two genes (LbOGS00358 and LbOGS05722) were considered as "gene death", because of their domain architectures. But they had the highest expression in the results of Fig 4d. Besides, the pattern of each period of the two genes is similar to other not "dead" genes. Can you explain the definition of "gene death" in more detail?
5. Fig 1f and Line 809. The method description of enrichment analysis is too brief without specific parameters and the enrichment model used. There are many tools in the analysis website mentioned in the method. Can you describe in detail which tool you are using?
6. The mucin-binding domain in Lb is almost completely absent in other Metazoan species. This result was very unusual. In these species, sequencing reads could be mapping to verify again whether there was no trace of muchin-bd at all?

Minor points:
1. Some supplement tables are not shown, such as supplement table 1-5, 7, 15.
2. "the Lh genome shows a secondary peak enriched with genomic windows of extremely low GC content (16%) (Supplementary Fig. 2)." The "extremely" in this sentence is too strong. From the Supplementary Fig. 2, only the second peak in Lh genome, not to the extreme level.

3.“The genomes of Leptopilina encode more olfactory receptor genes (ORs) than those of other parasitoids except Nasonia vitripennis, while they encode the fewest gustatory receptor genes (GRs).” This description about figure 1c is not very accurate.

4. in the first part of result, according to the differences in the genomes and genes of the two parasitic wasps to suggest that the evolution of host ranges in Leptopilina was unlikely to be driven by host location. I don't understand the connection between the two parts.

5. There are some typos in some places. For example LhOGS04370 should be LbOGS04370.

7. In the legend of Supplementary Figure 3, S.D is written as N.D.
8. Line 211. Since the definition of Lar and Lar’ not mentioned above, it is recommended to use the original names (LhOGS04147 and LhOGS20123).
9. Line 599. The brief description of the Fig 3 is mainly for Lar, but Fig 3b is mainly about muchin-bd (more related to Warm). The legend should be modified.
10. Line 660. Many programs do not identify version numbers, for example: GenomeScope, Canu, SMARTdenovo, Pilon, ScaffMatch, GapCloser etc.

Reviewer #3 (Remarks to the Author):

The authors have investigated the molecular basis of a generalist-specialist differentiation between two parasitoid wasps (Leptopilina heterotoma and L. boulardi parasitoids of Drosophila larvae). Genomic analyses of parasitoid-host interactions are extremely important for understanding the rapid evolution of these intense arms races. The manuscript represents an incredible amount of work that is very well put together and written up, and supported by numerous well-designed figures and tables. Although I am not a genomicist/bioinformatician, I can judge the life history findings and their adaptive significance, as well as the coverage of existing knowledge and literature, as generally sound. The conclusions seem well supported by the extensive experimentation and analyses, and the Discussion is well in balance with the findings.
I have some minor comments about the text.
The boundary between the Results and Discussion sections is not clear, the last part of the Results reads like a Discussion.
Consider providing a definition of parasitoid at the start of the abstract. The term may not be widely known among a broad readership
Line 52: delete “Therefore”
Line 61, 175, 261, and onwards: the use of “while” and “since”, while = whereas and Line 91: since = as [while and since are time-related terms]
Line 131-132. I do not understand this conclusion from the preceding information, what is meant with host location?
Line 149: add “gene” to repertoires, as lifecycles do not have functional repertoires
Line 259: add “of” to “as part a chimeric”
Line 293: strange use of the term “genomic imprints”
Line 332: evolved = evolve
Line 421: offsprings = offspring [already plural]
Lines 497-498: check grammar
Lines 500-501: check grammar
Lines 533-535: check grammar

Reviewer #1 (Remarks to the Author):

The authors of “Two horizontal gene transfers underlie divergent parasitic strategies between a generalist and a specialist parasite” look at several venom protein genes in the parasitoid wasps Leptopilina heterotoma (Lh) and L. boulardi (Lb) to determine their function and origin. The paper determines the function of Lh Lar, which triggers the lysis of the Drosophila lymph gland leading to the inhibition of the host encapsulation response, and Lb Warm, which assists in the attachment of wasp eggs to the internal organs of the host, providing physical protection against the complete encapsulation that helps parasites escape from the host cellular immune response. How these genes contribute to the wasp’s host range it also assessed. The paper further discusses the possible LGT origin of Lar and another gene mucin-db.

This is a very interesting study and I really enjoyed reading the manuscript. The paper is very well written and flows nicely. The analyses are thorough, and the conclusions are well supported. The figures are well put together and clearly illustrate the data. The only issue I had with the paper is the section of the LGT origins of Lar.

We appreciate your high evaluation on our study. Your criticisms and suggestions help improve the manuscript.

You didn’t convince me that Lar is an LGT in this section. Lar is undoubtably something very strange in the Lh and Lb genomes, but it would be unlike any other reported LGT before. First, you report that all the initial hits were only 20-30% sequence ID, which with such a short sequence leaves me to wonder if they are in fact really homologs of each other or just spurious BLAST hits. The iterative BLASTN-GeneWise approach further makes me worried if these genes are truly homologs or it just a chain of weak support strung together. What is the percent sequence id between the chalcid clade I and III and cynipid clade III Lar sequences? Adding some representative sequences from the other groups to Sup. Fig. 18 would help add support to you claim (Also Sup. Fig. 18 is mislabeled Sup. table 18 on line 852). You can also compare the predicted structure of these proteins (in PhyreII or something similar) to further add support to your conclusions that these are true homologs.

These *Lar* homologs were indeed highly diverged with each other, e.g. the amino acid sequence identity between homologs of *N. vitripennis* clade I and III is ~25%. However, all sequences retailed in the phylogenetic analysis share consensus G-motifs with >90% identity (as shown in the original Supplementary Fig. 18; now, Supplementary Fig. 20) at three scattered loci, i.e., G1: GxxxGKS/T (conserved across all involved species), G2: SxT (conserved between representative Cynipoidea and Chalcidoidea species), and G3: DxPGF (conserved across species), although G2 and G3 became diverged along the Lh-specific lineage expansion. We have added a new supplementary figure (Supplementary Fig. 21) to show detailed multiple alignment

across sequences of these representative species in the context of inferred phylogeny.

As per your suggestion, we used Phyre2 to predict 3D modeling structure of representative sequences. Sequences from all groups were able to be modeled with >99% confidence by a hydrolase template (c3zjcC, GTPase imap family member 7). This evidence further supports that these highly diverged sequences are putative homologs. We have mentioned these results in the revised manuscript (lines 215 and 314-315) and a new supplementary Fig. 11.

Regarding to the label issue on the original line 852 (now 874), we meant to indicate a supplementary table that lists all Arthropod species we used to perform the TBLASTN-Genewise search. Instead, supplementary Fig. 18 (now supplementary Fig. 20) presents the multiple alignment of *Leptopilina* homologs of *Lar*.

The other oddity of this is that there is no clear origin for this gene. Most LGTs are common in their clade of origin, for example the prevalence of mucin-bd gene in bacteria. There is no indication of where Lar originated and how it was exposed to Lh and Lb genomes to make the transfer. Let alone how it did all the other jumps (which should be increased from 3 to at least 8 because of the amount of losses it would take to correlate the Lar clades to the species tree). The fact that Clades I-III aren't nested within microorganisms needs to be addressed as well, does it suggests the gene is jumping directly from insect to insect or does the eukaryotic evolution of the gene make the appear more similar? For this story to be convincing you need to propose a hypothesis of how it moved from group to group. Do you think it has a viral origin? Is it a TE? Is parasitoid venom spreading it? Do all the genomes that have copies of Lar have extremely low GC content?

This is an important point. Due to lacking of closely similar sequences, we indeed did not precisely indicate the donor of LGT of *Lar*. However, our analyses present lines of evidence supporting the LGT origin of *Lar* and its homologs (the relationship between *Lar* and its homologs were addressed above). First, the presence of *Lar* and its homologs is jumping in the context of organism evolution, i.e. they were only found in a few Arthropods, non-metazoans, and prokaryotic species. Second, our phylogenetic analyses clearly placed these Arthropod homologs in three distinct clusters (clade I-III). Deep split between these clusters and discontiguously phylogenetic relationships both suggest different origins of these Arthropod homologs. Particularly, the presence of clade III homologs was strictly limited in parasitoids and mainly distributed in Cynipoidea clade. Since there are both clades I and III homologs in Chalcidoidea, we further performed an independent phylogenetic analysis using Chalcidoidea sequences only. The resulted tree clearly showed that clades I and III were separated by basal species (new Supplementary Fig. 21), indicating the transfers of I and III were unlikely to originate from insect to insect. Instead, LGTs of these different homologs might occur multiple times, each of which

has different fates. LGT of clade I is much more ancient, which probably originated prior to the divergence among Arthropods and underwent massive losses along lineages. However, the clade III, which arose *Lar*, likely took place prior to the divergence between Cynipoidea and Chalcidoidea wasps and was largely retained in Cynipoidea lineage (as shown in Fig. 3b).

We hare reorganized the paragraph to clarify the basic evolutionary scenarios of these LGTs (see lines 322-335). However, we were still unable to find closely similar sequences of Lar outside parasitoids, either due to the rapid evolution of these genes or the missing of the actual donor species in NCBI. Thus, we did not talk about too much about this aspect but left a note in the revised manuscript.

Regarding the GC content, we have scanned the GC content across all genomes having Lar homologs as shown in Fig.3. It is clear that only Lh has a remarkably low GC content and the unique secondary peak (see below). This unique feature might allow the massive spread of *Lar* homologs, yielding a massive expansion of ~100 copies, although it seems unrelated with the presence of *Lar* homologs across Arthropod species.

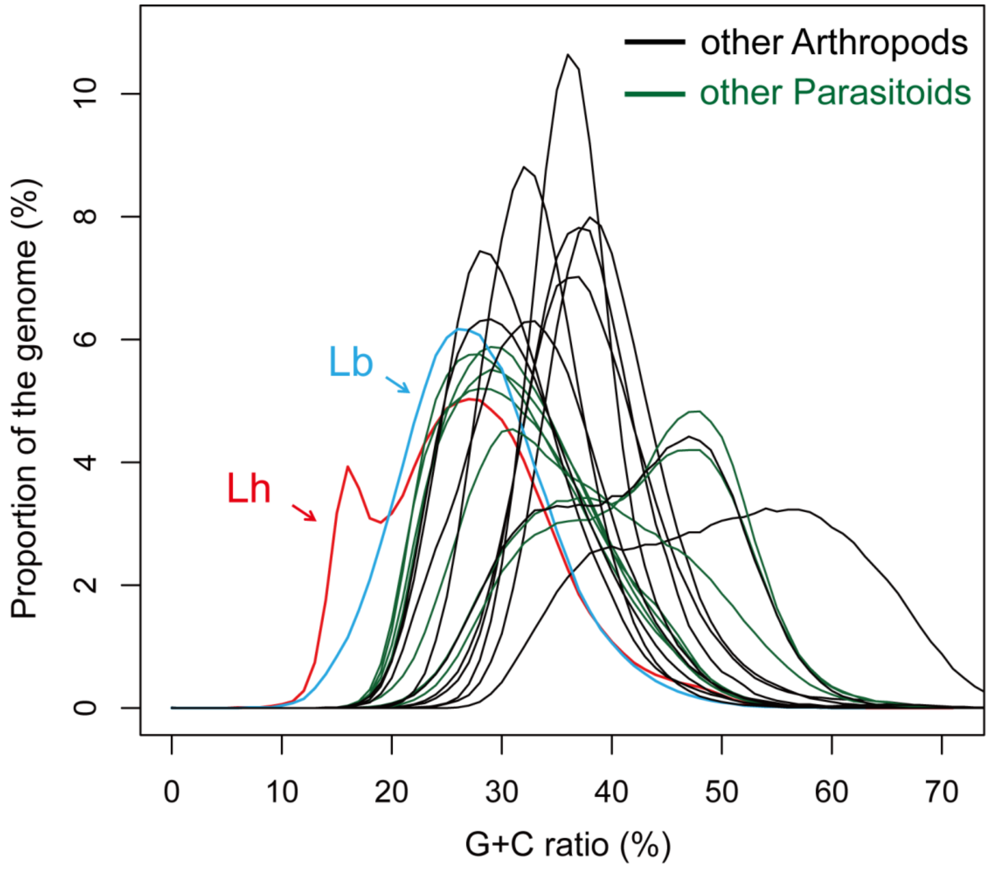

This paper is very interesting with very strong support for all your other conclusions, including that mucin-db gene is an LGT. I would suggest maybe not including the LGT story in the title of your paper and focusing more on your very solid other results,

with the LGT an interesting but slightly confusing side story.

Based on an overall consideration and further discussion with our colleagues, we feel better to change the title to “Two novel venom proteins underlie divergent parasitic strategies between a generalist and a specialist parasite”.

Minor comments:
-line 95: For the 16% of GC windows in Lh with extremely low GC content, are the windows scatter throughout the genome or grouped? Do they correspond to repetitive elements, centromeres, or any other genomic architecture?

These low GC windows are scattered throughout the genome. We found that a majority of assembled scaffolds (388 out of 411) have these low GC windows. Based on our annotations, these windows were found overlapping with different structures as follows: repetitive elements (50.6%), introns (24.7%), exons (2.2%), and intergenic regions (22.5%). These widespread low GC windows might allow the massive spread of *Lar* homologs.

Line 115: With an estimated 40 MY divergence time and only ~86% sequence identity, I would be very hesitant to call Lh and Lb sister species. For example, two sister species in the Nasonia clade are only separated by 0.5MY with 98% sequence identity. These two species seem to have a lot of evolution between them.

To avoid confusion, we have removed the term of “sister species” as suggested.

Lines 141-149: The PCA analysis in SupFig 5 does not support your conclusions. The PCA (which I would trust more than the cluster next to your expression heat map) shows that the life stages of Lh and Lb are quite similar in their expression, with the exception of P3.

We have modified the statement by highlighting the difference in P3 only (lines 143-144 and 148-149). P3 is actually an important stage which we discussed later, when Lh venom protein genes begin to massively express (see lines 164-171).

Line 214: Sorry if I missed it, but I didn’t see what bioinformatic analyses were tried to predict functions and domains in Lar. Might be good just to list some analyses. Did you try determining a function from a predicted protein structure, for example PhyreII?

We mainly ran local InterProScan with all integrated databases and performed online search against the NCBI’s conserved domain database to scan domains. Detailed information has been added in the revised manuscript (see lines 777-783).

As per your suggestion, we used Phyre2 to predict 3D structure of *Lar* and its

homologs. Related results have been described in the main text (lines 215 and 314-315) and shown in Supplementary Fig. 11.

Fig 3a have an arrow to Lar-Lar' on phylogeny as well as expression map.

We have added an arrow to indicate *Lar-Lar'* in Fig. 3a.

Missing or mislabeled supplemental tables

Since three supplementary figures were added, we have carefully rechecked all labeled supplementary materials.

Reviewer #2 (Remarks to the Author):

The authors have combined multiomic data, evolutionary analyses, parasitic efficiency assays and functional experiments to identify two horizontal gene transfers underlying divergent parasitic strategies between a generalist (Lh) and a specialist (Lb) parasite. The Lh-specific protein, Lar, enables active immune suppression by lysing the Drosophila lymph glands, eventually leading to successful parasitism by Lh. Meanwhile, the Lb-specific protein, Warm, may contribute to a passive strategy by attaching the laid eggs to the gut and other internal organs of the host, leading to incomplete encapsulation and helping Lb escape the host cellular immune response. The results provide an important reference for the in-depth study of the molecular mechanisms of different parasitic strategies between the generalist Lh and the specialist Lb.

We appreciate your overall positive comments on our study and further suggestions that help improve the manuscript.

Major points:
1. LhOGS20047 has a high level of protein expression and evidence of RNAi, but does not affect the efficiency of parasitism of Lh. Among this gene family, which sequence feature of Lar play a key role for parasitism function?

This is an important point but difficult to be resolved at present. *Lar* homologs evolved rapidly and greatly diverged with each other. As shown in the original supplementary Fig. 18 (now 20) and the new supplementary Fig. 21, *Lar* and its homologs showed diverged sequences at most loci, except the relatively conserved G-motif loci. We note that the sequence identity between *Lar* and *LhOGS20047* is only 35%, although they were both characterized as venom proteins and of highly specialized expression in venoms. Available public databases cannot predict any informative domains on the unique sequence of *Lar* that might confer its unique role in lysing the host lymph glands in parasitization. On the other hand, parasitoids are not model species, which lack of powerful techniques to study functions at specific sites as *Drosophila*. Unfortunately, classic transgenic and gene editing methods (such as CRISPR/Cas9) are impracticable for *Leptopilina* wasps, because this endoparasitoid species lay and hatch eggs in the body of host. In this study, we have developed an efficient RNAi system to knockdown target genes and used it to characterize functional relationships between a number of target genes and parasitism effects. We respectfully appreciate your point and agree that understanding of how sequences evolved and functionalized is important to further explore the diversified parasitism mechanisms in parasites. We expect to implement this upon the development of novel functional technologies and ideas in future. This point has been stated in the revised manuscript (see lines 353-354).

2. In the proteome study of Lh, the genes with the highest protein expression level are not lar and lar'. I wonder if those highest expressed proteins also have an effect on parasitic efficiency and thus parasitic function is contributed by a variety of venom proteins?

We mainly focused on *Lar* and its homologs, given their dominant roles across the top list (ranked as 1st, 6th, and 10th, highly expressed VPs, respectively), and indeed ignored other highly expressed VP genes. As per your suggestion, we have designed RNAi experiments for the remained seven VP genes of the top 10 list (i.e., *LhOGS06609*, *LhOGS10118*, *LhOGS01638*, *LhOGS01639*, *LhOGS01180*, *LhOGS02019*, and *LhOGS00546*), as well as two candidates with the highest peptide number (i.e., *LhOGS20077* and *LhOGS08557*). The qRT-PCR results showed that the expression levels of these nine genes were all significantly reduced in Lh adults upon the injection of corresponding dsRNA. However, the parasitism rate was not affected in the host parasitized by any of these dsRNA-treated wasp lines, except a significant reduction in the *dsLhOGS06609*-treated line. However, we note that, in comparison to *Lar*, this effect is marginal, and that knockdown of *LhOGS06609* cannot rescue the apoptosis of the host lymph glands. *LhOGS06609* is an Lh-specific gene without characterized function. Unlike the evident effect and the definite role of Lar in provoking cell death in the lymph gland, and hence, suppressing the host encapsulation response, this novel venom protein might play a minor role in leading to successful parasitization. It is uncertain that whether it involves in the active immune suppression of Lh. We have added these results in both main text (lines 250-261) and a new supplementary Fig. 16.

3. The assembly quality of the genomes of these two species is very different. The annotated gene numbers differ by more than 800. These differences may affect the accuracy of certain gene categories of Supplementary Figure 3. It would be better to discuss this in the paper.

Despite a different size of N50, both assembled genomes present a high level of completeness. Moreover, we indeed performed a completely consistent pipeline to predict genes for both species, including the identical lines of homolog inputs and closely similar sets of transcriptome data (Supplementary Table 7). The overall features between two yielded gene sets are quite similar, particularly in term of mean exon number per gene which is probably affected by the fragmented assembly. Regarding Supplementary Fig. 3, we found that the main difference between Lh and Lb sets lies in the content of “Patchy” orthologs. This class of orthologs includes genes with a jumping presence across unrelated species along with variable copy numbers, indicating they might experience rapid evolution and turnover across species.

We also note that Lh and Lb diverged with each other approximately 40 Mya (Fig. 1c). Within such evolutionary scale, the difference in gene numbers is not uncommon

between related insect species. For example, the genomes of 12 *Drosophila* species (divergence within 40 Mya) showed variable numbers among the gene sets, ranging from 13,733 to 17,325 (*Nature* 450: 203-218; see below).

Table 2 | A summary of annotated features across all 12 genomes

	Protein-coding gene annotations		Non-coding RNA annotations					Repeat coverage (%)*	Genome size (Mb; assembly†/flow cytometry‡)
	Total no. of protein- coding genes (per cent with *D. melanogaster* homologue)	Coding sequence/ intron (Mb)	tRNA (pseudo)	snoRNA	miRNA	rRNA (5.8S + 5S)	snRNA		
D. melanogaster	13,733 (100%)	38.9/21.8	297 (4)	250	78	101	28	5.35	118/200
D. simulans	15,983 (80.0%)	45.8/19.6	268 (2)	246	70	72	32	2.73	111/162
D. sechellia	16,884 (81.2%)	47.9/21.9	312 (13)	242	78	133	30	3.67	115/171
D. yakuba	16,423 (82.5%)	50.8/22.9	380 (52)	255	80	55	37	12.04	127/190
D. erecta	15,324 (86.4%)	49.1/22.0	286 (2)	252	81	101	38	6.97	134/135
D. ananassae	15,276 (83.0%)	57.3/22.3	472 (165)	194	76	134	29	24.93	176/217
D. pseudoobscura	16,363 (78.2%)	49.7/24.0	295 (1)	203	73	55	31	2.76	127/193
D. persimilis	17,325 (72.6%)	54.0/21.9	306 (1)	199	75	80	31	8.47	138/193
D. willistoni	15,816 (78.8%)	65.4/23.5	484 (164)	216	77	76	37	15.57	187/222
D. virilis	14,680 (82.7%)	57.9/21.7	279 (2)	165	74	294	31	13.96	172/364
D. mojavensis	14,849 (80.8%)	57.8/21.9	267 (3)	139	71	74	30	8.92	161/130
D. grimshawi	15,270 (81.3%)	54.9/22.5	261 (1)	154	82	70	32	2.84	138/231

4. Line 392. The two genes (LbOGS00358 and LbOGS05722) were considered as “gene death”, because of their domain architectures. But they had the highest expression in the results of Fig 4d. Besides, the pattern of each period of the two genes is similar to other not “dead” genes. Can you explain the definition of "gene death" in more detail?

We meant to claim potential gene death based on the substantial sequence degradation in these two genes. In comparison to other homologs, their transcript length are extremely short and lack of any additional homologous segments (as shown in yellow, orange, and purple lines in Fig. 4D), except the mucin-bd domain. Due to lacking of further evidence, we have removed the term of “gene death” to avoid confusion.

5. Fig 1f and Line 809. The method description of enrichment analysis is too brief without specific parameters and the enrichment model used. There are many tools in the analysis website mentioned in the method. Can you describe in detail which tool you are using?

We apologize for the inappropriate organization of methods regarding this part. Actually, the enrichment analysis of Fig. 1f was based on the hypergeometric test by comparing the highly expressed genes with the whole gene set (background). The calculation was performed using the “phyper” module of R. The original sentence on line 809 was actually about the pathway enrichment analysis of the dN/dS part. We have moved this sentence to the appropriate context and added complete information for both related parts (lines 819-821 and 831-832).

6. The mucin-binding domain in Lb is almost completely absent in other Metazoan species. This result was very unusual. In these species, sequencing reads could be mapping to verify again whether there was no trace of muchin-bd at all?

Our conclusion was made based on both BLASTP search against the complete NCBI

NR database (non-redundant protein sequences, without limiting organisms) and the full documented records in InterPro (www.ebi.ac.uk/interpro) and Pfam (pfam.xfam.org). These public databases broadly collect and receive data without selecting biases on organisms, so they are perhaps the best venues for investigating the presence of a given gene with the maximum coverage and without bias.

Unlike the scattered distribution across a few Arthropod species of *Lar*, searching of *Warm* against NR did not hit any eukaryotic proteins. Thus, we no longer concerned genomic traces. It is likely that a given gene was occasionally lost in a few species due to the incompleteness of gene prediction and/or genome assembly, but it is unlikely that this gene was lost in EVERY species due to bioinformatics issues, even including those model species being assembled and annotated with the best quality.

Nevertheless, we have performed TBLASTN (under the e-value of 0.05) against the full NT database (non-redundant nucleotide sequences, without limiting organisms) to check whether there is any untranslated transcripts (e.g. pseudo genes) being omitted in the protein databases, and further against the RefSeq genome database (limiting Metazoan genomes, including 570 databases). As a result, the former search only detected prokaryotic hits while the latter one detected no hit (see below).

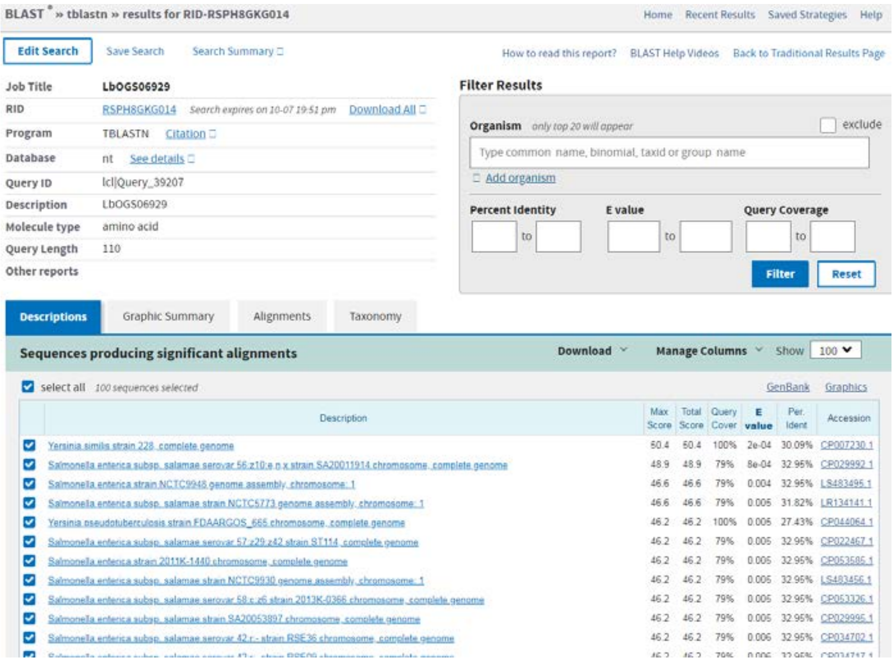

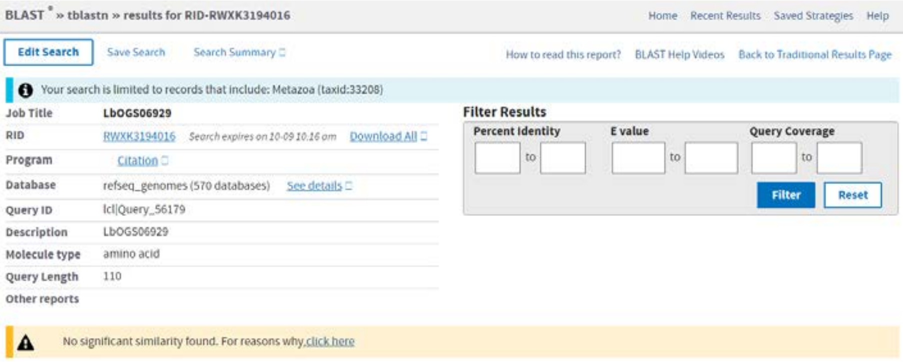

We did not directly search the domain on unassembled reads, which is uncommon for distantly related species and extremely time-consuming. There are probably thousands of metazoan species being sequenced, and our previous searches have shown no traces in hundreds of species. Again, it is unlikely that a given gene was lost in every deposited species due to the incomplete assembly. We believe the presented evidence should be solid enough to support an LGT origin of mucin-bd domain in Lb, which was probably transferred from prokaryotes to Leptopilina directly.

Minor points:

1. Some supplement tables are not shown, such as supplement table 1-5, 7, 15.

These tables were actually presented in the combined PDF file of supplementary material, following the supplementary figures. Long tables were separately included in the Excel file. We have renamed the Excel file as "Supplementary long tables" to avoid confusion.

2. "the Lh genome shows a secondary peak enriched with genomic windows of extremely low GC content (16%) (Supplementary Fig. 2)." The "extremely" in this sentence is too strong. From the Supplementary Fig. 2, only the second peak in Lh genome, not to the extreme level.

The 16% of GC content is indeed quite low for a higher animal species. To avoid confusion, we have changed "extremely" to "remarkably".

3."The genomes of Leptopilina encode more olfactory receptor genes (ORs) than those of other parasitoids except Nasonia vitripennis, while they encode the fewest gustatory receptor genes (GRs)." This description about figure 1c is not very accurate.

Here, we compared the gene family size of ORs and GRs between *Leptopilina* with other parasitoids species (only for those in the grey shadow, rather than all species in the tree). We have highlighted and extended the shadow to help avoid confusion (new Fig. 1c).

4. in the first part of result, according to the differences in the genomes and genes of the two parasitic wasps to suggest that the evolution of host ranges in Leptopilina was unlikely to be driven by host location. I don't understand the connection between the two parts.

We talked about the evolution of chemoreception and environment-sensing related gene families in the preceding paragraph. Unexpectedly, we found these host-seeking gene families either evolved conserved across species or showed inconsistent pattern in terms of gene family changes with the host range. Thus, we hypothesized that the host range between Lh and Lb was probably not to be driven

by the phase of host seeking. We have replaced the original statement with a more straightforward sentence ("These genomic signatures did not provide evidence to support a role of environment-sensing modules, e.g. host seeking, in driving the change of host ranges in Leptopilina.") and left further details in the Discussion part (lines 535-541).

5. There are some typos in some places. For example LhOGS04370 should be LbOGS04370.

We apologize for any typos in the manuscript. This erroneous gene ID has been corrected. We have further checked typos throughout the manuscript.

7. In the legend of Supplementary Figure 3, S.D is written as N.D.

The typo has been corrected.

8. Line 211. Since the definition of Lar and Lar' not mentioned above, it is recommended to use the original names (LhOGS04147 and LhOGS20123).

We agree with your point that the occurrence of names prior to the definition makes no sense. We have modified this place as "the between-sample dN/dS".

9. Line 599. The brief description of the Fig 3 is mainly for Lar, but Fig 3b is mainly about muchin-bd (more related to Warm). The legend should be modified.

In Fig. 3b, all red cells indicate *Lar* homologs while the blue cells indicate *Warm* homologs. We meant to borrow the species tree here to present the distribution of *Warm* in the context of species evolution. We have added related notes in this figure legend (line 630).

10. Line 660. Many programs do not identify version numbers, for example: GenomeScope, Canu, SMARTdenovo, Pilon, ScaffMatch, GapCloser etc.

We apologize for providing incomplete information for some softwares. We have checked this issue throughout the manuscript and added the version information correspondingly.

Reviewer #3 (Remarks to the Author):

The authors have investigated the molecular basis of a generalist-specialist differentiation between two parasitoid wasps (Leptopilina heterotoma and L. boulardi parasitoids of Drosophila larvae). Genomic analyses of parasitoid-host interactions are extremely important for understanding the rapid evolution of these intense arms races. The manuscript represents an incredible amount of work that is very well put together and written up, and supported by numerous well-designed figures and tables. Although I am not a genomicist/bioinformatician, I can judge the life history findings and their adaptive significance, as well as the coverage of existing knowledge and literature, as generally sound. The conclusions seem well supported by the extensive experimentation and analyses, and the Discussion is well in balance with the findings.

We appreciate your high rate on our study and detailed suggestions that help improve the manuscript.

I have some minor comments about the text.
The boundary between the Results and Discussion sections is not clear, the last part of the Results reads like a Discussion.

We organized this part to extend the ecological contexts of Lar and Warm. We designed parasitization assays and presented important experimental results in this part (corresponding to Fig. 5). Thus, we think it’s better to keep this section as a part of Results. We are also happy to hear further suggestions on how to reorganize the context.

Consider providing a definition of parasitoid at the start of the abstract. The term may not be widely known among a broad readership

This is a great idea. We have added a definition of parasitoid at the beginning of the abstract (lines 24-25).

Line 52: delete “Therefore”

It has been deleted as suggested.

Line 61, 175, 261, and onwards: the use of “while” and “since”, while = whereas and Line 91: since = as [while and since are time-related terms]

Thank you. They have been corrected as suggested.

Line 131-132. I do not understand this conclusion from the preceding information, what is meant with host location?

We apologize for the jumping statement here, which was also criticized by another referee. The preceding paragraph is talking about the evolution of chemoreception and environment-sensing related gene families. We found these host-seeking gene families either evolved conserved across species or showed inconsistent pattern in terms of gene family changes with the host range. Thus, we hypothesized that the host range between Lh and Lb was unlikely to be driven by the phase of host seeking.

To make it read more smooth, we have replaced the original statement with a more straightforward sentence ("These genomic signatures did not provide evidence to support a role of environment-sensing modules, e.g. host seeking, in driving the change of host ranges in Leptopilina.") and left further details in the Discussion part (see lines 535 to 541).

Line 149: add "gene" to repertoires, as lifecycles do not have functional repertoires

Thank you, but the related statement has been removed based on the comment of another referee.

Line 259: add "of" to "as part a chimeric"

It has been corrected as suggested.

Line 293: strange use of the term "genomic imprints"

We have changed this sentence as "To characterize the traces of Lar as completely as possible, we directly searched for its potential homologs in the genomic sequences of Lh and Lb."

Line 332: evolved = evolve

It has been corrected as suggested.

Line 421: offsprings = offspring [already plural]

It has been corrected as suggested.

Lines 497-498: check grammar

This sentence has been reworded as "The co-option of single-copy genes has been shown an important role in the evolution of new gene functions in chalcidoid wasps.

Lines 500-501: check grammar

This sentence has been reworded as “Previous studies reported that …”.

Lines 533-535: check grammar

This sentence has been reworded as “Despite the widespread presence in cynipoid parasitoids, all putative homologs of *Lar* outside Lh were evidently diverged with those specialized expressed in the VGs.”

Reviewer #1 (Remarks to the Author):

The authors have addressed all my previous comments, I recommend acceptance of this manuscript.

Reviewer #2 (Remarks to the Author):

no more comments

Reviewer #3 (Remarks to the Author):

The authors have dealt with my (minor) comments satisfactorily. This is a beautiful piece of (a lot of) work.

Reviewer #1 (Remarks to the Author):

The authors have addressed all my previous comments, I recommend acceptance of this manuscript.

We appreciate your input that helps improve the study.

Reviewer #2 (Remarks to the Author):

no more comments

Reviewer #3 (Remarks to the Author):

The authors have dealt with my (minor) comments satisfactorily. This is a beautiful piece of (a lot of) work.

We appreciate your high evaluation and input that helps improve the study.
